# Machine Learning Techniques Applied to Dose Prediction in Computed Tomography Tests

**DOI:** 10.3390/s19235116

**Published:** 2019-11-22

**Authors:** Antonio-Javier Garcia-Sanchez, Enrique Garcia Angosto, Jose Luis Llor, Alfredo Serna Berna, David Ramos

**Affiliations:** 1 Department of Information and Communication Technologies, Universidad Politécnica de Cartagena (UPCT), Campus Muralla del Mar, E-30202 Cartagena, Spain; joseluisllor@gmail.com; 2 General Electric Healthcare, E-28023 Madrid, Spain; enrique.garciaangosto@ge.com; 3 Hospital General Universitario Santa Lucía, E-30202 Cartagena, Spain; alfredo.serna@carm.es (A.S.B.); davidramosamores@hotmail.com (D.R.)

**Keywords:** machine learning, dose, computed axial tomography, patients

## Abstract

Increasingly more patients exposed to radiation from computed axial tomography (CT) will have a greater risk of developing tumors or cancer that are caused by cell mutation in the future. A minor dose level would decrease the number of these possible cases. However, this framework can result in medical specialists (radiologists) not being able to detect anomalies or lesions. This work explores a way of addressing these concerns, achieving the reduction of unnecessary radiation without compromising the diagnosis. We contribute with a novel methodology in the CT area to predict the precise radiation that a patient should be given to accomplish this goal. Specifically, from a real dataset composed of the dose data of over fifty thousand patients that have been classified into standardized protocols (skull, abdomen, thorax, pelvis, etc.), we eliminate atypical information (outliers), to later generate regression curves employing diverse well-known Machine Learning techniques. As a result, we have chosen the best analytical technique per protocol; a selection that was thoroughly carried out according to traditional dosimetry parameters to accurately quantify the dose level that the radiologist should apply in each CT test.

## 1. Introduction

Nowadays, one of the most frequently carried out medical tests is the so-called computed axial tomography (CT), which is used to obtain a precise human body image while using X-Rays [1]. Radiologists are able to observe anomalies or lesions in patients without performing other invasive techniques by means of this non-invasive technique. This is the reason why the number of CT-based tests has grown enormously in recent years [2]. However, an inadequate dose of X-rays delivered to the human body over time could result in serious diseases, even increasing the risk of cell mutation, which can lead to the proliferation of tumors. In addition, the younger the patient is, the greater the probability that he/she could develop cancer due to increased cellular activity [3].

Keeping the well-known risks of computed tomography-related radiation in mind, there is another factor to take into account; the clarity of the image. The higher the dose applied to the body, the higher the quality of the image, which therefore makes it easier for radiologists to make diagnoses. Thus, adjusting the dose that is received by a patient is mandatory for allowing the detection and accurate identification of lesions or diseases, even in their earliest stages, while minimizing the possible risks for patients. For instance, a well-known practice among radiologists is ALARA (As Low As Reasonably Achievable) [4]. This consists of reducing radiation exposure by computed tomography scanning, encouraging the use of alternative techniques, such as ultrasound and magnetic resonance, while also maintaining the efficiency and reliability of clinical diagnoses.

Guided by the current concern about patient healthcare, the scientific community establishes dose reference levels (DRL) [5], statistically calculated from the gathering of data on doses received by patients radiated by different CT machines at the regional and national level. The goal of dose reference levels is to determine an upper limit, while considering those values that are above these limits to be dangerous. The results of DRL differ from the protocol employed; that is, DRL values depend on the part of the human body studied in the computed tomography test. There are different protocols (skull, thorax, abdomen, pelvis, or combinations of them), where, if required, medical staff will administer a contrast medium to increase the accuracy of the diagnosis [6].

According the authors’ knowledge, the academic community, medical staff, or practitioners lack broad and detail-oriented studies that adjust the radiological doses to the patient’s morphology or size while considering the aforementioned protocols. The objective of this work is twofold to address this drawback. First, we accurately predict the doses of radiation that patients should receive by means of artificial intelligence systems; in particular, the subset denoted as Machine Learning (ML) techniques. Applied to CT protocols, they improve the results that were obtained by the current metrics used in conventional radiology (Computed Tomography Dose Index—CTDI_VOL_—[7] or Size-Specific Dose Estimate—SSDE—[8]). Second, predictive techniques will, in many cases, result in lower thresholds than standardized DRLs.

We determine the most appropriate ML technique for the top five most often used protocols from thousands of studies classified into their corresponding protocols to achieve these objectives. These cover nearly 92% of all CT examinations, thus comparing different regression schemes and providing a useful tool that allows for us to plan the dose that each patient should receive. This tool helps radiologists or medical staff in the decision-making process. They are the ones who should make the final decision based on their clinical experience or knowledge. Nevertheless, the system is already trained to learn and act automatically when radiologists consider this to be advisable. We will focus our study on eight large hospitals in the Region of Murcia, located in the Southeast of Spain, collecting all of the dose data from May 2015 to December 2017 for each protocol.

## 2. Terminology

The dosimetry measurements used in this work are defined in the following paragraphs:

• CTDI_VOL_ (Computed Tomography Dose Index):

This is a measurement of the absorbed dose in a slice of a standard volume, which is characterized by a cylinder or phantom. The most common measurements are 16 cm in diameter to simulate the head of the patient and 32 cm for the body. It is important to mention that this is not the real dose absorbed by the patient, but a measurement of the radiation output from the scanner. It is measured in mGy and its value is intrinsically related to other parameters set in the device, such as voltage or current.

Due to this, it is a good indicator for quantifying dosimetry changes when scanner parameters are modified, which helps to relatively identify high or low radiation values for a specific protocol. Furthermore, as CTDI_VOL_ is physically measured, it is also useful for detecting whether a CT machine is working properly or not.

• SSDE (Size-Specific Dose Estimate):

The SSDE parameter provides a better approach to the dose applied in each slice, while considering the real size of the patient, due to the fact that the morphology of a patient does not necessarily fit the standard sizes employed in the CTDI_VOL_ metric (16/32 cm). To this end, it is necessary to carry out a *scanogram* before performing the CT test on a patient. This technique determines the morphology of the patient to adjust the radiation in each slice. The importance of SSDE is clear. It is able to set personalized doses to patients, preventing, for instance, smaller patients from receiving more radiation than required.

• DRL (Dose Reference Level)

The DRL level indicates the dose that is received by a patient or the amount of radiopharmaceutics administered for a certain medical exam. This reference level determines whether a dose is unusually high or low for that exam.

• BMI (Body Mass Index)

Body mass index (BMI) is a metric that relates the mass and weight of an individual. BMI is defined as the mass of the individual (expressed in kg) divided by his/her height squared (expressed in m^2^), providing a value that helps us to interpret his/her morphology [9].

## 3. Related Work

In the scientific literature, we can find diverse works related to the radiologic dosimetry field and its associated technical parameters. Many of them compared the delivered dose in relation to patient morphology. Thus, in [10], the authors analyzed the relationship among patient size, the radiation delivered by the scanner (CTDI_VOL_), and the dose estimated for the size of the patient by means of the anteroposterior (AP) and lateral (LAT) lengths for thorax examinations. In this study, authors observed a high linear correlation between patient size and the CTDI_VOL_ metric. Unlike the results of previous studies, the authors in [10] demonstrated that the SSDE metric is independent of patient size. Following this line, the work [9] evaluated the use of both body mass index (BMI) and weight in thorocoabdominal tests, obtaining the SSDE metric from CT scans. BMI and weight showed a high correlation with the *effective diameter* in adults, making their use possible as a substitute in the calculation of SSDE. However, BMI demonstrated a greater correlation with the *effective diameter* than the weight of the patient in abdominal CT probes, while, in chest CTs, the results indicated that weight was more closely correlated with the *effective diameter* than the figure of merit BMI.

On the other hand, the scientific community is currently recognizing the value of using Big Data and Machine Learning techniques in the medical field. In this regard, work [11] highlighted the importance of data to improve clinical practices and diagnoses. Access to information regarding thousands of patients and its subsequent analysis allows for us to obtain models and patterns that can be used for personalized medical treatments. Furthermore, the application of ML techniques in the medical field is emerging as a technique that supports, among other things, image reconstruction in CT tests. Under these premises, in 2017, a model was proposed in [12] to enhance image clarity by reducing the number of projection views, achieving better edge reconstruction. In the work [13], the authors designed a solution to locating the limits of liver tumors in CT images, while employing a specific machine learning technique (AdaBoost learning). This algorithm learned how to classify border points as true or false. In [14], the authors aimed to develop a classification algorithm to segment seven biological tissues in the neck, chest, abdomen, and pelvis through CT images. To this end, tools such as Matlab and its ML Random Forest toolbox assured a clear segmentation of “shapes”, such as air, bone tissue, fat, or muscle.

Currently, another medical research area in which Machine Learning has had great impact is that related to radiological doses. In 2015, the work [15] extensively analyzed three ML models (*logistic regression*, *support vector machines*, and *neuronal networks*) to predict whether a patient would suffer from symptoms after receiving a certain radiotherapy dose. In 2018, the work [16] proposed radiotherapy plans in patients with oropharyngeal cancer. KBP (knowledge-based planning) was employed from a twofold perspective: the bagging query method (BQ) and the generalized principal component analysis-based method (gPCA). The main goal was to predict Dose Valued Histograms [17], which relate doses to tissue volume. The gPCA method showed results that were similar to the clinical plans; however, the BQ solution differed greatly. Finally, the work [18] dealt with the detection of lesions in CT from the image perspective, in particular to assisting the radiologist in the recognition of nodules in the lung while using a deep learning technique. Note that the analysis of CT images is out of the scope of this work. Authors in other research fields apply this know-how in ML techniques [19].

The aforementioned works applying ML techniques to dosimetry in CT tests contribute with spot advances, making specific improvements. We offer a step forward to achieve a broader solution considering the most remarkable protocols. We precisely predict the radiation level that a patient should receive by analyzing a set of well-known ML techniques, selecting the best for each protocol. Furthermore, as a second contribution, we compare these values to the DRLs that were obtained by the scientific community, suggesting recommendations and guidelines.

## 4. Materials and Methods

The Ethics Committee for Clinical Research, which accepted the waiver of the requirement to obtain patient informed consent, approved the study.

### 4.1. Data Collection

This work is supported by dosimetry data that were collected from thirteen CTs operating in eight hospitals in the Region of Murcia (Table 1), between May 2015 and December 2017.

The total number of analyzed exams was 58,571. In addition to the diagnosable image, the data attached to the exams include information regarding the gender, the type of protocol (listed in Table 2), the dose received by each patient in terms of CTDI_VOL_ and SSDE metrics (if applicable), the type of phantom, the CT employed, the age of the patient, and the BMI metric for those exams carried out from 2015 to 2017.

The DRL figure associated to CTDI_VOL_ metric is also included in this work. DRLs are obtained through studies of dosimetry that were carried out in different countries. A summary of DRL values per country/protocol can be found in [20,21], where we have selected the most representative ones: Belgium (2017), Denmark (2015), Finland (2013), France (2012), Germany (2010), Greece (2013), Ireland (2011) [22], Luxembourg (2001), Norway (2010), Poland (2011), Sweden (2002), Switzerland (2018) [23], the Netherlands (2012), and the United Kingdom (2011).

Matlab is the mathematical tool selected to manage and analyze the entire data, in its version R2017a, together with its Statistics and Machine Learning Toolbox libraries. The simulations were executed in a computer whose main features are an Intel Core i7-8700K processor and 16 GB of RAM memory.

### 4.2. Methodology

**Figure 1:**
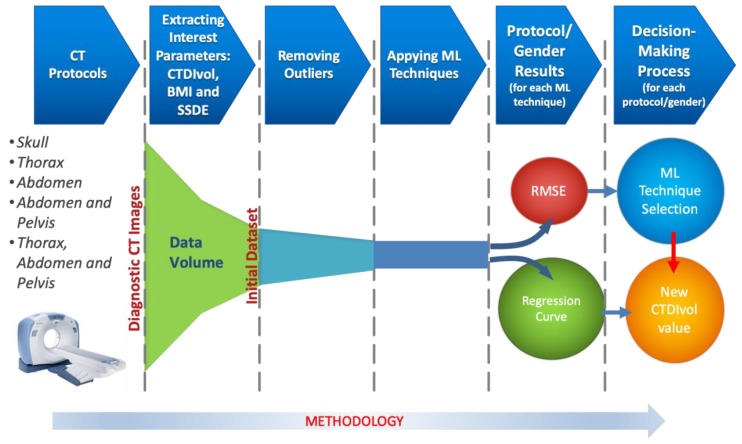
Methodology to predict future doses in patients radiated by CT.

To achieve an appropriate prediction of the dose to be radiated in patients, it is necessary to carry out the following steps (see Figure 1):

1. From all of the CT tests, the radiology team sets the diagnosable images by protocol. The remaining images, the non-diagnosable ones, are not considered in our work.
2. A CT test contains a large amount of information (sequence of images, patient’s data, physical magnitudes, etc.). From all of them, we extract the following interest parameters: CTDI_VOL_, BMI, and SSDE (if the CT has this feature). These parameters comprise the Dataset.
3. It is necessary to discard the data that can jeopardize the prediction of future results. This phase consists of removing this unrepresentative information, which is also called *outliers*.
4. Applying different well-known Machine Learning techniques to each protocol, we obtain the regression curve that best fits the data.
5. In the decision-making process, we select the best ML technique, while employing an objective metric as the *root of the quadratic mean error (RMSE),* along with the computational cost.
6. Once the ML technique is selected, a precise CTDI_VOL_ value is calculated from the regression curve, taking the BMI (or SSDE) as the input parameter. The CTDI_VOL_ value is, a priori, the new calculated dose to deliver to the patient.

#### 4.2.1. Removing Outliers

Once the data are gathered, those considered to be atypical (outliers); that is, values denoted as errors or unrepresentative are eliminated from the population (in this work, the sample values match the population). There are diverse techniques in order to do this [24], which are applied according to the distribution of the sample and the data percentage to be removed.

**Figure 2:**
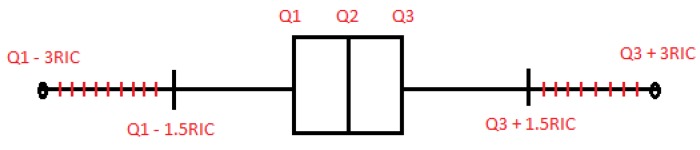
Boxplot.

If the data are treated as separated variables, outliers are eliminated while using the *univariate method*. Figure 2 shows the boxplot diagram that was proposed by Tukey in 1977 [25], in which the distribution of a set of data is observed, and different regions are identified from their statistical information.

As Figure 2 shows, we define the *Interquartile range* (RIC) as the difference between Q3 (third quartile or 75th percentile) and Q1 (first quartile or 25th percentile). In this way, if we want to remove extreme values, those greater than Q3 + 3 *RIC and those less than Q1 − 3 *RIC will be eliminated. Even if we intend to be more restrictive, data that are higher than Q3 + 1.5 *RIC and lower than Q1 − 1.5 *RIC can be eliminated from our samples. However, the *univariate method* has a clear drawback; the data removed are located at the ends of each variable and outside of these zones there could be undetected outliers.

Another way of eliminating outliers is to employ the *multivariate method*. This is ideal for internal areas with low data density; meaning the number of data is not very representative in the whole sample.

**Figure 3:**
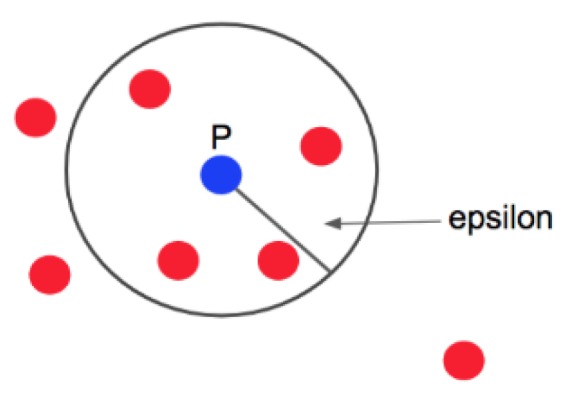
Density-based spatial clustering of applications with noise technique (DBSCAN) detection.

To detect and remove outliers, we apply the technique called the *density-based spatial clustering of applications with noise technique*, DBSCAN [26]. In this technique, the *epsilon* (euclidean distance) and *MinPts* (threshold) parameters must be previously defined, to later operate, as follows: for each particular data the number of neighbors in a certain *epsilon* must be quantified. If that number exceeds the established threshold (*MinPts*), the specific data and their neighbors are included in a cluster, as well as the neighbors of the previous data that fulfill the same condition. The iterative process continues until all of the data are checked and a cluster of connected data is established. On the other hand, if the number does not exceed the value of *MinPts*, the specific data will be considered to be noise, and will therefore be eliminated from the sample (see Figure 3).

The goal of both techniques is to eliminate the smallest number of data, trying not to exceed 5% of the total data of the sample. Once the atypical data have been removed, the regression techniques that are described in Section 4.2.2 will be applied to the remaining data.

The data are separated into two groups, called training and test, in order to obtain a good regression model [27]. The *cross-validation* technique will be used, which divides the sample into *k* groups of data. One of them will be used as a test and the rest for training.

This process is repeated during *k* iterations, with each time using a different group as test, without repetition. The value of *k* will vary depending on the number of available data. The value of 10 is the one that is selected for this work.

**Figure 4:**
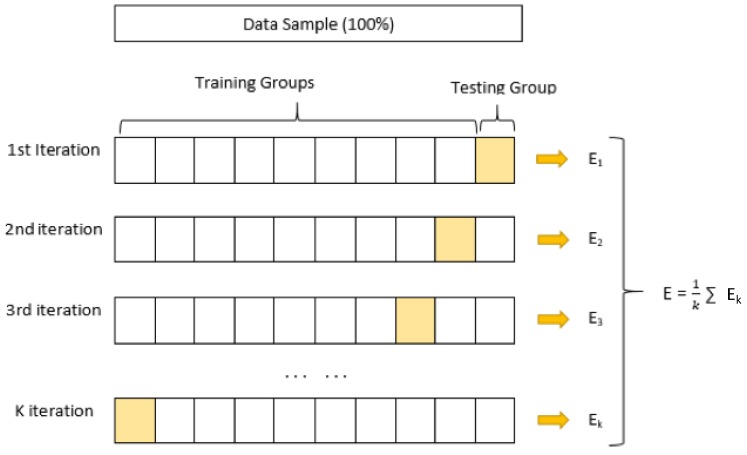
Cross Validation method.

The regression algorithm will be fed with the number of data allotted to training, which will fit a curve with these data, obtaining the mathematical model as a result. For each iteration, the error *E_k_* will be estimated; the mean of all the errors results in the total error *E*, as shown in Figure 4.

After training the algorithm and obtaining the model, we will verify its performance by means of new data that have not been employed in the training process. We use the test data for this purpose.

If the error from the test data is much greater than the error committed by the training data, the model is overfitting the training data, diminishing the generality for the test set. The reason is the following: the algorithm extracts a large amount of information from the dataset to generate the training data, deriving a complex model that is capable of precisely adjusting its predictions. This model can include noise or random fluctuations due to the great number of data. When assessing new data (test), a minor amount of them are selected, which implies a low probability of noise/random data. The result is a clear deterioration in the performance of the model. This issue has a negative effect on the precision of the predictions, to the point of making it unfeasible for the problem contemplated here.

#### 4.2.2. ML Techniques

Machine learning (ML) algorithms, as a subfield of artificial intelligence (AI), have been providing effective solutions in engineering applications and to scientific problems for many decades. The ML methods have the ability to adapt to new conditions and detect and estimate patterns. To this end, ML conceives self-learning algorithms to derive knowledge from data in order to carry out system predictions. ML provides a suitable solution that captures the awareness present in data and gradually enhances the performance of predictive models to build models that analyze a large amount of data. The main goal is to make the best decisions, or to take the best actions based on these predictions.

ML is divided into three categories: supervised learning, unsupervised learning, and reinforcement learning. In this work, we will focus our attention on supervised learning techniques, since they allow for a model to learn from training data to make predictions about unseen or future data. In supervised learning, the input data are defined by labels (such as, for instance, mail labels) or raw data. One of the subcategories of supervised learning is regression analysis, which addresses the prediction of continuous results from labels/raw data. Given a set of variables, *x*, named predictors, and their corresponding response variables, *y*, we can fit a curve graph (the simplest is a straight line) to these data that minimizes the distance between the sample points and the fitted linear/non-linear graph. The set unsupervised learning and regression analysis is adjusted to the requirements while observing the nature of the data to analyze in this work, allowing for us to predict the continuous outcomes of target variables.

In regression analysis techniques, the scientific literature presents different approaches that are useful in the massive analysis of data (Big Data). Furthermore, these techniques help in the forecasting of future doses to be received by patients, which is a distinctive objective of this work.

Regarding regression analysis models, we will concentrate our efforts on specifying (data selection), accommodating (eliminating outliers and anomalous points), and analyzing our large amount of CT exam data by using the following models: 

1. *Linear regression*.

This technique consists of finding a line that fits a data set following a certain criterion. The most common criterion, which will also be employed in this work, is least squares adjustment [28].

2. *Decision Tree Learning*.

This scheme breaks down our data by making decisions based on asking a series of questions. In particular, in the training phase, the decision tree model learns questions that are used to stamp class labels on the samples. As a tree model, the process starts at the root of the tree and then splits the data along its branches. The splitting procedure is repeated at each child node up to the leaves (of the tree). This means that the samples of each node belong to the same class. Note that the error is minimized if the tree is deep, but it can lead to overfitting. Thus, the usual procedure is to prune the tree, restricting its maximum depth. A better way to improve the results of the *Decision Tree Learning* algorithm is to employ a technique, called *Bagged Decision Tree,* which reduces the variance of a decision tree.

3. *Bagged Decision Tree*.

In this technique, multiple *regression trees* are constructed. In particular, several subsets of data are created from training samples, for each collection of them to be later used to train their own decision trees. The average derived from these different decision trees provides a more robust solution than a single decision tree. The use of several trees also reduces overfitting.

4. *Artificial Neural Networks* [29].

Our focus will be on analyzing the data for the training phase with a technique, called *Bayesian regularization* [30]. This algorithm allows for us to perform binary classification, and we will use the *Levenberg-Marquardt optimization* [31] to learn the weight coefficients of the model (in each iteration of the training phase, the coefficients are updated). Furthermore, it is possible to obtain the optimal weights employing cost functions, such as those called *Sum of Squared Errors* (SSE). To find the predicted values, the solution involves connecting multiple single neurons to a *multi–layer feedforward neural network*. This particular type of network is also called a *multi–layer perceptron (MLP)*, which consists of three layers (input, hidden, and output layers). Both techniques (*Bayesian regularization* and *Levenberg–Marquardt* optimization), together with an infrastructure MLP achieve an optimal model capable of generalizing the mathematical problem thanks to the minimization of a combination of weights and errors. This algorithm allows for overfitting to be reduced at the cost of longer execution time.

5. *Gaussian Process Regression* [32].

Parametric regression methods, for instance, linear/logistic regression, generate a line or a curve in the graph of inputs and outputs, replacing the training data. Accordingly, once the regression weights have been obtained, the original training data may be eliminated from the graph. On the other hand, non-parametric regression methods may retain the initial training data (also called latent variables) to be used as a significant element in generating a regressor function. To this end, test data are compared to the training data points; each output value of the test point is estimated via the distance of the test data input to the training data input. It is notable that non-parametric regression considers that data points with similar input values will be close in output space. The mathematical expressions include the covariance function formed of latent variables, which reflects the smoothness of the response. Covariance and mean functions were used in conjunction with a Gaussian likelihood for prediction, employing $f_{*}|X,y,X_{*}\;$as an initial expression. In it, $f_{*}\;$is a posterior distribution, *X* is a matrix of training inputs, *y* is a vector of training target, and $X_{*}\;$is a matrix of test inputs.

To maximize this expression, we have carefully studied the mathematical model that was derived in [33]. From this previous study, we opt as a useful equation for the problem here planned the following exponential function, which will, in turn, be employed as a kernel function:

(1) $$k_{exp}\left(x,x^{\prime }\right)\;=\;\sigma_{f}^{2}\;\exp\left(-\sqrt{\overset{d}{\underset{k\;=\;1}{\sum }}\frac{{\left(x_{i,k}\;-\;x_{j,k}\right)}^{2}}{l_{k}^{2}}}\right)$$

where the parameter $\sigma_{f}^{2}$ is the standard deviation, while l_k_ is the scalar dimension for each predictor, *k* is the number of evaluations to fulfill the maximization problem, and *x* and *x’* are two near values.

6. *Support Vector Regression* (*SVR*) [34].

In this case, we consider the following training data $\left\{\left(x_{i},y_{i}\right),\ldots ..,\left(x_{l},y_{l}\right)\right\}$, where $x_{i}\subset R^{n};\;y_{i}\subset R\;$indicate the input space of the sample and its corresponding target value, respectively, and *l* denotes the size of the training data. Our objective is to find a function f(x) that has, at most, ε deviation from the obtained targets $y_{i}$ for all of the training data, and at the same time is as flat as possible. In other words, we do not care about errors because they are less than ε. Additionally, the results must avoid senseless predictions to find a function f(x) that returns the best fit.

Regarding the relationship between x and y, it is approximately linear, which means that the model is represented as: $f\left(x\right)\;=\;\langle w,x\rangle \;+\;b;\;w\;\subset \;R^{n}\;;\;b\;\subset \;R$ (*w* represents coefficients and *b* is an intercept). Therefore, this problem can be formulated as a convex optimization problem:

$$minimize\;\frac{1}{2}\;\|w{\|}^{2}$$

(2) $$subject\;to\{\begin{array}{c} y_{i}\;-\;\langle w,x_{i}\rangle \;-\;b\;\leq \;\varepsilon \\ \langle w,x_{i}\rangle \;+\;b\;-\;y_{i}\;\leq \;\varepsilon \end{array}$$

Here, our optimization problem is planned as a non-linear case. Keeping this in mind and, thanks to the support of the work [35], the solution for (2) is the following Equation (3):

(3) $$max\{\frac{1}{2}\;\overset{l}{\underset{i\;=\;1}{\sum }}(\alpha_{i}\;-\;\alpha_{i}^{*})(\alpha_{j}\;-\;\alpha_{j}^{*})\langle \varphi (x_{i}),\varphi (x_{j})\rangle \;-\;\varepsilon \;\overset{l}{\underset{i\;=\;1}{\sum }}(\alpha_{i}\;+\;\alpha_{i}^{*})\;+\;\overset{l}{\underset{i\;=\;1}{\sum }}\;y_{i}(\alpha_{i}\;-\;\alpha_{i}^{*})\;$$

$$s.t.\;\overset{l}{\underset{i\;=\;1}{\sum }}(\alpha_{i}\;-\;\alpha_{i}^{*})\;=\;0\;;0\;\leq \;\alpha_{i},\alpha_{i}^{*}\;\leq \;C$$

The constant *C* > 0 determines the trade-off between the flatness (this means that one seeks a small *w* value) of *f* and the amount up to which deviations that are larger than ε are tolerated. On the other hand, $\alpha_{i}$, $\alpha_{i}^{*}$, $\alpha_{j},\;and\;\alpha_{j}^{*}$ are Lagrange multipliers. Finally, $\langle \varphi (x_{i}),\varphi (x_{j})\rangle \;=\;K\left(x_{i},y_{i}\right)$ is a Kernel function. A common kernel that is used for this model is the radial basis function: $K\left(x_{i},y_{i}\right)\;=\;\;e^{-\frac{\|xi\;-\;yi{\|}^{2}}{2\sigma^{2}}}.$

A more detailed study of these aforementioned techniques can be found in the Supplementary File 1 “Description of Machine Learning Techniques”. Finally, Table 3 shows the parameters used in the previously defined algorithms: 

#### 4.2.3. RMSE Metric

We employ the *root of the quadratic mean error (RMSE)* metric as a measure of error. The RMSE is a value that measures the standard deviation of the error. This is calculated by Equation (4) as the average of errors squared, with *n* being the number of samples, *y* the real value, and $\hat{y}$ the predicted value. The RMSE metric presents a range from zero to infinite, especially punishing those data that are far from the estimated value.

(4) $$\mathrm{RMSE}\;=\;\sqrt{\;\frac{1}{n}\;\overset{n}{\underset{j\;=\;1}{\sum }}{\left(y_{j}\;-\;{\hat{y}}_{j}\right)}^{2}}$$

When the relationship between two variables is obtained by means of *linear regression*, we can also employ the parameter *R* (correlation coefficient) in the analysis, which shows the degree of linear correlation between the variables. When *R* approaches 1 or −1, there is a high linear correlation. However, if its value is close to 0, both of the variables are said to be poorly correlated.

## 5. Results

In this section, two different comparisons are accomplished to predict the precise dose levels that a patient should receive to obtain a diagnosable image. Firstly, we analyze the radiation output of the CT set by the CTDI_VOL_ parameter versus BMI, which depends on the patient’s height and weight. Secondly, we compare the same figure of merit CTDI_VOL_ with respect to the SSDE metric, which returns his/her exact morphology. Although the SSDE parameter adjusts, a priori, the dose level better than BMI, the unavailability of collecting it in some of the CTs (see Table 1), together with the possibility of discerning gender, have motivated this twofold study. Finally, it is notable that a powerful tool for dose optimization during CT examinations is the analysis of outliers, as we have shown in a previous work [36].

### 5.1. Comparison between CTDIvol and BMI

The main objective is the prediction of the optimal dose of radiation that a patient should receive, while taking into account his/her BMI and the type of protocol. The parameter predicted is CTDI_VOL_, so this will allow for technicians to adjust different parameters to achieve that radiation value in CT output. Additionally, gender-segmented regression data for the five protocols indicated in Table 2 will be calculated while using each one of the ML techniques described above. However, we have opted to only show the best ML technique to shorten the length of the paper and make the text more understandable to readers. The remaining studies can be found in the Supplementary File 2 “Results involving ML, Protocols, and dose metrics”.

Two figures are plotted for each protocol/gender. The first one illustrates the regression curve after removing the outliers, together with the European DRL reference values. The second shows an error histogram composed of 20 bars. This figure represents both training and test errors and it highlights the relationship between both. The *x*-axis indicates the error made; that is, the distance between the real data and the value predicted, while the *y*-axis points out the number of data that have an error of that magnitude.

#### 5.1.1. Skull Protocol

In this protocol, the data dispersion is clearly high, which demonstrates low linear correlation between BMI and CTDI_VOL_. In the men’s case, the correlation coefficient R^2^ reaches a value of 0.000908 when all of the samples are adjusted by means of a *linear regression*. A slight improvement is obtained if the anomalous data (outliers) are not computed; the value reached for R^2^ is 0.0467. As in the men’s case, for women, a very low linear correlation between BMI and CTDI_VOL_ is attained. By adjusting all of the samples by means of a *linear regression* the parameter R^2^ reaches a value of 0.000154. When outliers are eliminated there is a slight improvement; R^2^ increases to 0.0348. For both genders, when the total number of samples is analyzed, *Bagged regression trees* is the technique that best adjusts the training and test data. This conclusion comes from analyzing the results of Table 4 and Table 5.

The elimination of outliers was carried out in two phases. Firstly, from the set of samples, those values that were higher than Q3 + 3 *RIC, and those lower than Q1 - 3 *RIC were ruled out through the univariate method, as described in Section 4. Secondly, samples that were placed in areas with low data density, far from the usual values, and with significant influence on the error, were also removed. To this end, we employ the multivariate technique also mentioned in the previous section. Note that this procedure was carried out in a similar way in the rest of the protocols analyzed in this section.

After the elimination of outliers, *bagged regression trees* continues as the best predictive technique, obtaining the lowest RMSE of all the ML techniques analyzed. However, the same performance is not achieved with the test data, showing a big difference between both (training and test data).

The *Neural Networks* technique provides excellent RMSE results for the test data and, therefore, for the model presented. This technique also fits for the training data, with smooth transitions and avoidance of overfitting. Thus, *Neural Networks* is the most suitable solution for predicting future doses for the "Men’s/Women’s Skull" protocol and the CTDI_VOL_-BMI plots. *Gaussian processes* is another technique that produces results that are similar to those of *Neural Networks*. Nevertheless, the execution time of *Gaussian processes* is the longest, which is an inconvenience when the number of samples increases.

**Figure 5:**
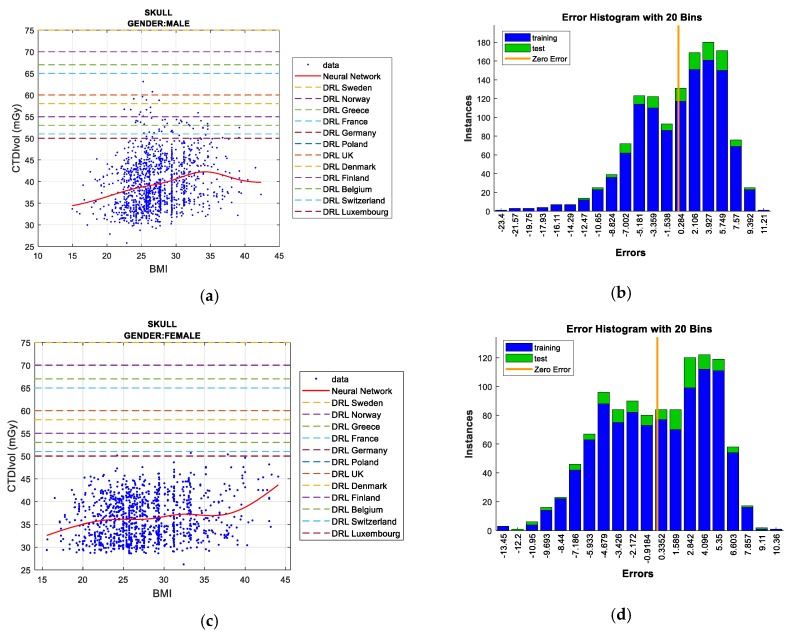
Radiation delivered by the scanner (CTDI_VOL_) prediction according to BMI including European DRLs for men’s/women’s skull protocol (**left**), together with error histograms (**right**), employing the *Neural Networks* technique: men (**a**,**b**), and women (**c**,**d**).

Finally, it should be noted that the obtained dose does not exceed the DRL value for any of the European countries comprised in this work, as shown in Figure 5.

#### 5.1.2. Thorax, Abdomen, and Pelvis Protocol

Regarding men, there is low linear correlation between BMI and CTDI_VOL_ (R^2^ = 0.00583), which is greatly enhanced when the outliers are eliminated (R^2^ = 0.283). In the case of women, the linear correlation between BMI and CTDI_VOL_ is low (R^2^ = 0.000355), which substantially increases when the outliers are eliminated from the computation (R^2^ = 0.304).

*Gaussian processes* obtains the best result (when 100% of the samples are computed), deriving the lowest value of RMSE in the test data, as shown in Table 6 and Table 7. *Bagged regression trees* achieves the least error in the training data (as occurred in previous cases). Removing outliers, *Gaussian processes* and *Neural Networks* are the techniques that accomplish the least error in the test data for men and women, respectively, and it can be said that both are the best predictive models for the “Thorax, Abdomen, & Pelvis” protocol.

However, as in the aforementioned protocol, there are no clear differences in terms of RMSE values if we compare all of the ML techniques. It should be noted that *GPR* is the technique with the highest computation demand.

**Figure 6:**
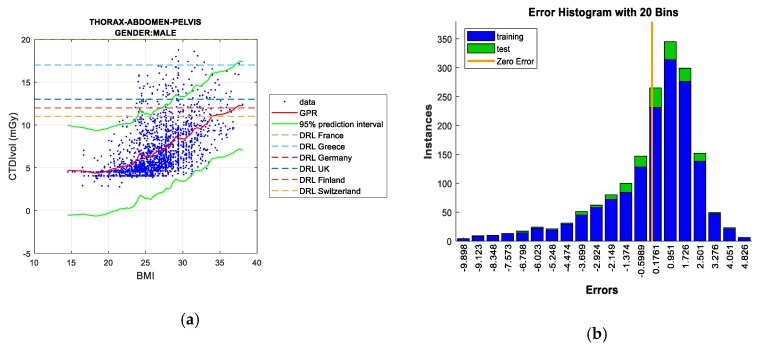

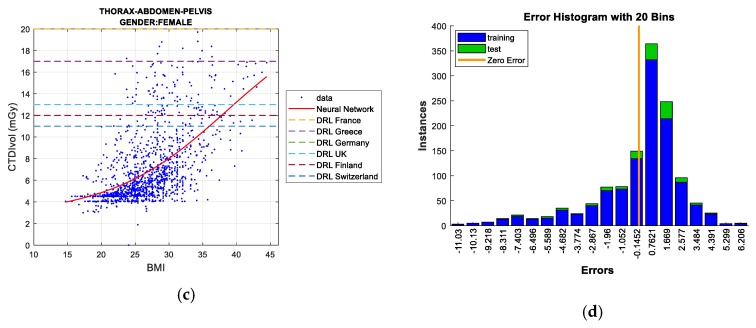
CTDI_VOL_ prediction according to BMI including European DRLs for men’s/women’s thorax, abdomen & pelvis protocol (**left**), together with error histograms (**right**), employing the *Gaussian Process Regression* (GPR) technique for men; (**a**,**b**), and the *Neural Networks* for women; (**c**,**d**).

Figure 6a,b, concerning men, show the *Gaussian processes* results always below most of the European DRLs, with the exception of only the most restrictive DRL (Switzerland), which has been exceeded by BMI values of about 35. In the case of women (Figure 6c,d), *Neural Networks* outcomes illustrate the surpassed DRLs are Switzerland and Finland when the BMI exceeds the value of 35. The following most restrictive DRL (UK) is exceeded with a BMI value close to 40.

#### 5.1.3. Abdomen and Pelvis Protocol

In the men’s case, there is a low linear correlation between BMI and CTDI_VOL_ (R^2^ = 0.00741), which sharply increases when the outliers are eliminated (R^2^ = 0.313). As shown in Table 8, *bagged regression trees* provides the best result (when all of the samples are computed), achieving the lowest RMSE in the test data. In contrast to the previous cases, *Gaussian Process Regression* (*GPR*) obtains the least error in the training data. However, the *GPR* technique implies a high computational cost. *Bagged regression trees* achieves the least error for the set of training and test data (with suppression of outliers) and therefore, it is the best predictive model for this protocol.

**Figure 7:**
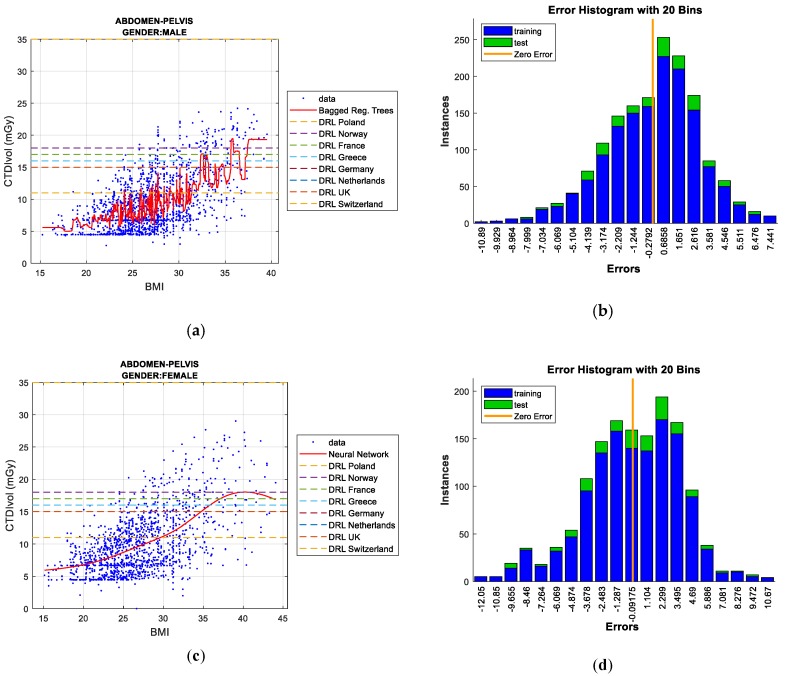
CTDI_VOL_ prediction according to BMI including European DRLs for men’s/women’s abdomen & pelvis protocol (**left**), together with error histograms (**right**), employing the ‘*bagged*’ *regression trees* (**a**,**b**) for men and the *Neural Networks* (**c**,**d**) for women.

Figure 7a,b illustrate how the *bagged regression trees* technique exceeds the most restrictive DRL (Switzerland) when the BMI reaches a value of around 32. A value greater than 35 is required to exceed the DRLs established by the rest of the European Union (EU) countries.

Regarding women, there is a low linear correlation between BMI and CTDI_VOL_ (R^2^ = 0.00329), which improves when outlier data are eliminated (R^2^ = 0.338). As observed in Table 9, *Neural Networks* and *bagged regression trees* achieve the best results for the test data and training data, respectively. When the outliers are removed, *Neural Networks* reach the best data adjustment, obtaining the least error in the test data. Regarding the DRL metric, Figure 7c,d indicate results that are similar to the men’s case.

#### 5.1.4. Thorax Protocol

Regarding men, the linear correlation between BMI and CTDI_VOL_ (R^2^ = 0.00729) is low. The R^2^ value increases to eliminate outliers (R^2^ = 0.283). As pointed out in Table 10, *Gaussian Process Regression* (*GPR*) provides the best results in the test data, since the lowest RMSE values are reached with this solution. In the case of the training data, the *bagged regression trees* technique is the most notable. When we eliminate outliers, *Neural Networks* satisfies the least error in the test data, so it attains a better prediction for the umbrella of these requirements (BMI-CTDI_VOL_, protocol, and gender). In this regard, *GPR* and *linear regression* also offer good results, although we choose *Neural Networks* as the best technique.

**Figure 8:**
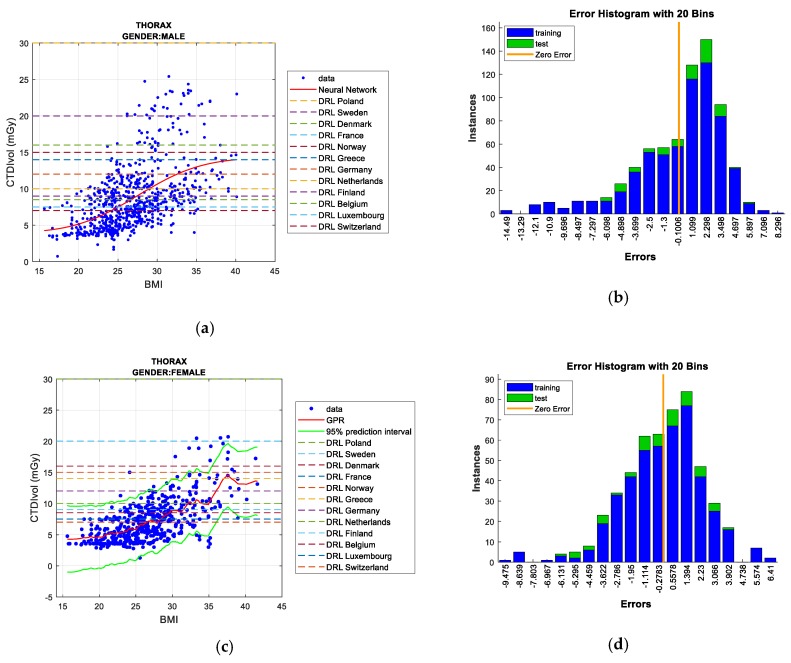
CTDI_VOL_ prediction according to BMI including European DRLs for men’s/women’s thorax protocol (**left**), together with error histograms (**right**), employing the *Neural Networks* (**a**,**b**) for men and the GPR (**c**,**d**) for women.

As shown in Figure 8a,b, some European DRLs are surpassed by *Neural Networks* plots for BMI values of about 25 (as occurs with the DRLs of countries, such as Switzerland or Luxembourg). However, our samples are mainly lower than the rest of standardized DRLs, such as those of Greece, Norway, or France.

In the women’s case, the R^2^ value obtained points to a low linear correlation between BMI and CTDI_VOL_ (R^2^ = 0.0261); this value is enhanced when outlier data are removed (R^2^ = 0.412). *Neural Networks* offers the best result for test data when the outliers are not eliminated. Under these conditions and as shown in Table 11, *Gaussian Process Regression* (*GPR*) reaches the least error in the training data. In the case of removing outliers, *GPR* is the best prediction technique, providing the least error during test data. However, while observing the RMSE values, note that most techniques provide good performance, although *GPR* and *Neural Networks* imply high computational cost. As illustrated in Figure 8c,d, the exceeding of several European DRLs starts from slightly higher BMI values in the case of women for this protocol than for men.

#### 5.1.5. Abdomen Protocol

Regarding the men’s case, a medium/high linear correlation is observed between BMI and CTDI_VOL_ (R^2^ = 0.555), which slightly increases as few outlier points are removed from all of the samples (R^2^ = 0.586). As shown in Table 12, *bagged regression trees* is that which obtains the least error in the training data process and computing 100% of the data. *Gaussian processes* and *Neural Networks* achieve the lowest RMSE in the test data when all of the samples are analyzed. *Neural Networks* and *Gaussian processes* are the most efficient models in removing outliers, and thus, both ML techniques behave better in terms of prediction functionality. However, the latter requires the highest computational cost.

In the case of women, there is a certain linear correlation between BMI and CTDI_VOL_ (R^2^ = 0.197), which substantially increases when outlier points are eliminated (R^2^ = 0.618). As can be observed in Table 13, and while considering outliers, *Gaussian processes* obtains the lowest RMSE for the test data. Under these same conditions, *bagged regression trees* reaches the least error for the training data. *Neural Networks* and *Gaussian processes* both offer the best adjustments in removing outliers, and therefore, they are the best solutions for predicting future doses in patients according to their weight and height (BMI). Although *GPR* is that which requires more computational means for the simulations, it is the technique selected.

**Figure 9:**
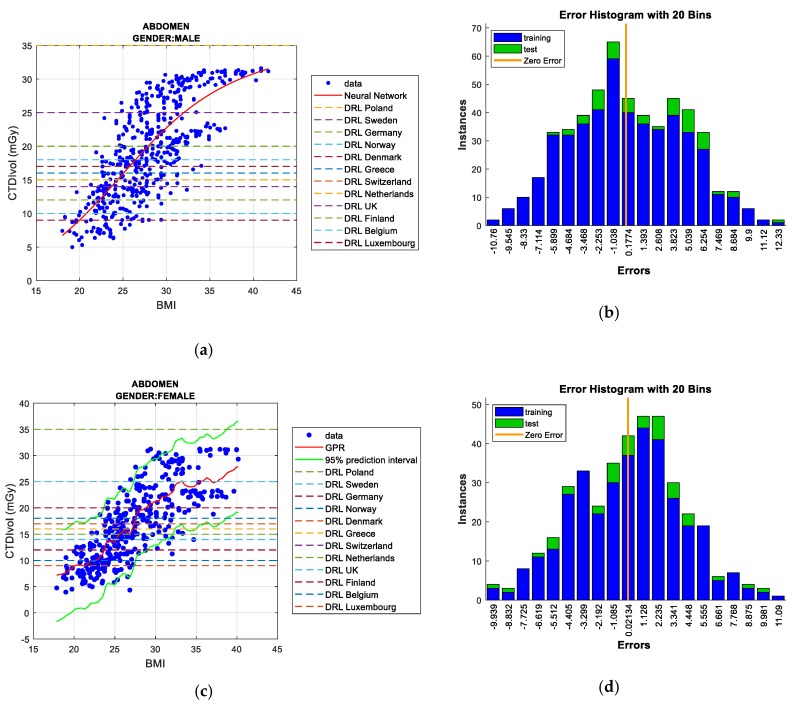
CTDI_VOL_ prediction according to BMI including European DRLs for men’s/women’s abdomen protocol (**left**), together with error histograms (**right**), employing the *Neural Networks* (**a**,**b**) for men and the GPR (**c**,**d**) for women.

For any gender, Figure 9 illustrates the surpassing of the DRL values that were established by diverse European countries when the BMI metric is around the value of 20. *Neural Networks* and *GPR* models exceed all DRLs (excepting the DRL of Poland) for BMI values higher than 30.

### 5.2. Comparison between SSDE and CTDI_VOL_

CTDI_VOL_ is a metric that is provided by the output of the CT and standardized on a reference volume. However, it is not the real dose received by the patient, since it does not consider his/her morphology. That is, a patient can receive a different amount of radiation than the one indicated by the CTDI_VOL_ value, because his/her morphology usually differs from the standard volume. To address this problem, SSDE is a parameter that computes the morphology of the patient from a *scanogram.* Therefore, SSDE is a more reliable measurement of the dose that is received by patients. However, not all current CTs have the ability to collect this information (see Table 1).

Figures analyzing CTDI_VOL_–SSDE metrics give us the required knowledge of the actual dose delivered to the patient from any CT with minimum error. To predict future CTDI_VOL_–SSDE values beforehand implies knowing the dose to radiate for the set patient and protocol. To achieve this, a CTDI_VOL_–SSDE regression study is carried out, employing the ML techniques described in this work. Specifically, we have also eliminated univariant/multivariant outliers from the data that were collected during the years 2015 and 2016 for the five protocols under study, as indicated in Table 2.

As in the previous section, two figures are drawn for each of the cases. The first illustrates the regression curve, while the second shows an error histogram with 20 bars, plotting both training and test errors and observing their differences. The *x*-axis represents the introduced error; that is, the distance between the real data and the predicted value, while the *y*-axis indicates the data number that has an error of specific magnitude. As in the CTDI_VOL_-BMI study, we only highlight the most representative ML technique. The remaining results for each ML technique can be found in the “Results involving ML, Protocols, and dose metrics” section in the Supporting Information for description.

In this study, note that there is no separation between men and women, because the SSDE parameter is focused on the morphology/shape of the patient and, therefore, obviates the need to identify the patient as a man or woman.

#### 5.2.1. Skull Protocol

**Figure 10:**
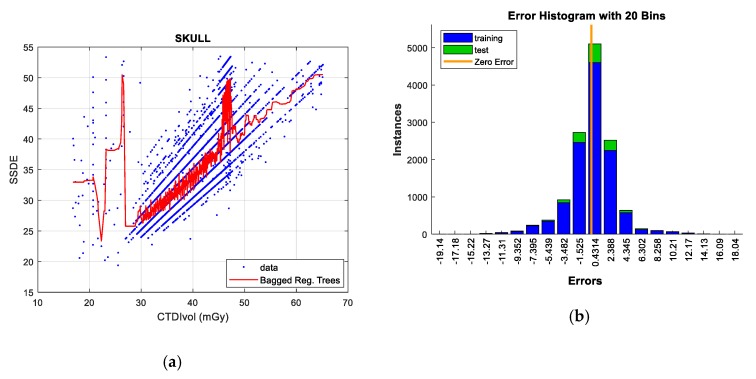
Size-Specific Dose Estimate (SSDE) prediction according to CTDI_VOL_ for skull protocol (**left**), together with error histograms (**right**), employing the ‘*bagged*’ *regression trees* technique (**a**,**b**).

As shown in Figure 10, this protocol is characterized by its high data dispersion. However, a certain linear correlation between CTDI_VOL_ and SSDE is observed (R^2^ = 0.193).

In the Skull protocol, while considering the whole population, the *bagged regression trees* and *GPR* techniques obtain the least error in test data and training data, respectively (as illustrated in Table 14).

The outliers were eliminated using the following criterion in order to reduce error. Firstly, applying the univariate technique, values that were greater than Q3 + 1.5 *RIC and values less than Q1 − 1.5 *RIC were left out of this evaluation. In this regard, the high dispersion of the data is a factor to consider. Secondly, additional samples belonging to areas with low data density, and those showing a significant influence on the error were also removed. Under these considerations, the linear correlation (R^2^ = 0.283) and effectiveness of the prediction improve in comparison with the processing of raw data.

When the outliers are suppressed, the RMSE is reduced by up to 47%, reaching the best results with the aforementioned techniques. Under these conditions, the *bagged regression trees* technique predicts better than the rest of the models. *Neural Networks* and *GPR* also offer an acceptable performance, while *SVR* and *linear regression* do not achieve good data adjustment. These facts are also corroborated in their corresponding error histograms. Finally, the *GPR* and *Neural Networks* models result in more processing and computation cost than the rest of the techniques, as in the case of the BMI-CTDI_VOL_ study.

#### 5.2.2. Thorax, Abdomen, and Pelvis Protocol

There is a high linear correlation between CTDI_VOL_ and SSDE (R^2^ = 0.914), which indicates a notable data adjustment with a very low error, independent of the analyzed technique.

*GPR* and *Neural Networks* attain the least error (when we compute all of the data) for test data and training data, respectively (see Table 15).

Some outliers were removed from the raw data following the same procedure described in the ’Skull’ protocol, but with one exception: to eliminate data employing the univariate method, the Q3 + 3 *RIC and Q1 − 3 *RIC were selected as threshold values in order to achieve a better result in the adjustment. Following this rule, the error was significantly reduced, further improving the linear correlation (R^2^ = 0.958), and therefore, the effectiveness of the prediction. It should be considered that this procedure would be carried out in the rest of the protocols for the CTDI_VOL_-SSDE prediction.

**Figure 11:**
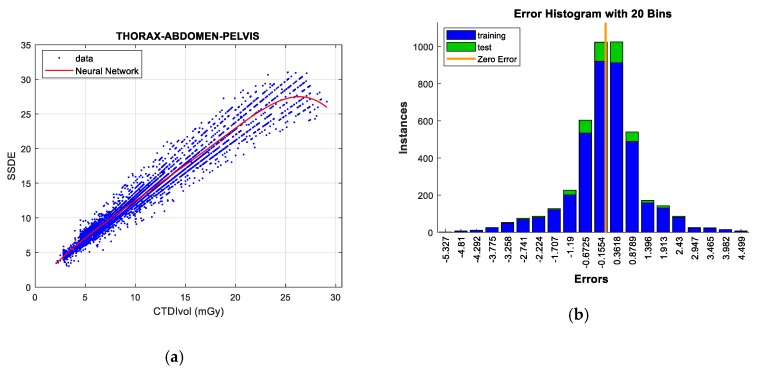
SSDE prediction according to CTDI_VOL_ for Thorax, Abdomen, & Pelvis protocol (**left**), together with error histograms (**right**), employing the *Neural Networks* technique (**a**,**b**).

By removing outliers (Figure 11) we can reduce the RMSE value by up to 38% in *Neural Networks*, making it the best predictive technique for this protocol. *Gaussian processes* also obtains remarkable performance, but at the expense of computation concerns. On the contrary, *SVR* and *linear regression* are penalized in this protocol, having the highest errors.

#### 5.2.3. Abdomen and Pelvis Protocol

As in the previous protocol, there is a high linear correlation between CTDI_VOL_ and SSDE (R^2^ = 0.704). When 100% of the data are trained, *bagged regression trees* provides the best result, as shown in Table 16. However, this is not the case for the test data, which are adjusted by *Gaussian processes* and *linear regression*.

When outliers are removed using univariant and multivariant methods, the correlation coefficient grows to a value of 0.894, which improves the linear correlation between CTDI_VOL_ and SSDE.

With this scenario, two techniques stand out for prediction tasks. *Bagged Regression trees* obtains the least error in training data, while *Gaussian processes* reduces the test error up to 55% in comparison with the remaining techniques. As in previous studies, the computation time of this technique is much longer than that of the rest of the models.

**Figure 12:**
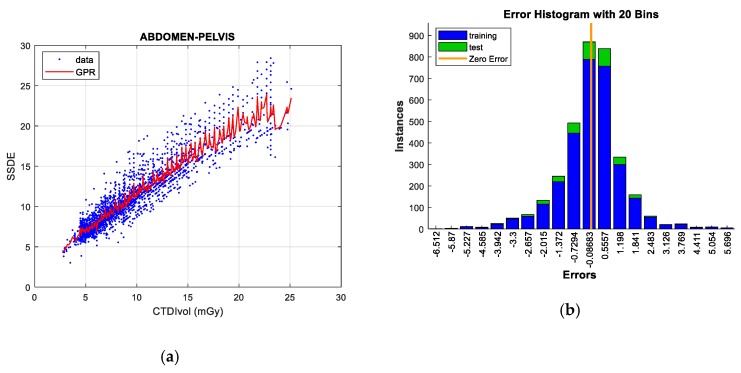
SSDE prediction according to CTDIvol for Abdomen & Pelvis protocol (**left**), together with error histograms (**right**), employing the *GPR* technique (**a**,**b**).

Finally, note that *linear regression*, *SVR,* and *Neural Networks* do not achieve good performance in data adjustment. *GPR* regression figures are grouped in Figure 12.

#### 5.2.4. Thorax Protocol

An appreciable linear correlation between CTDI_VOL_ and SSDE is observed in the thorax protocol and according to Table 17 (R^2^ = 0.572). The best result for the training data is reached with the model *bagged regression trees* (all of the data are computed). In contrast, the techniques *Gaussian processes* and *Neural Networks* are those that offer a better adjustment of the test data.

By eliminating atypical or unrepresentative data, the correlation coefficient increases up to a value of 0.907, which implies a substantial enhancement in the linear correlation between CTDI_VOL_ and SSDE.

**Figure 13:**
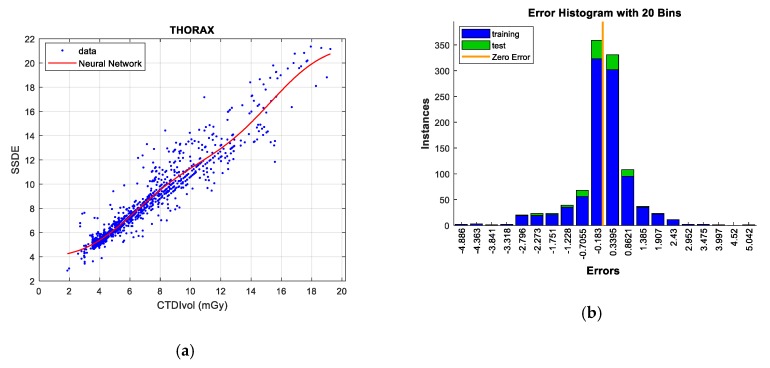
SSDE prediction according to CTDI_VOL_ for Thorax protocol (**left**), together with error histograms (**right**), employing the *Neural Networks* technique (**a**,**b**).

When training the samples without outliers, all of the techniques perform well, appropriately adjusting data in addition to significantly reducing the RMSE value. We want to emphasize, as in other occasions, the efficiency of *Gaussian processes* and *Neural Networks*, since they reduce the error to less than one unit, being, therefore, the most remarkable predictive techniques for this protocol. Similar to the previous scenarios, the *GPR* model stands out in terms of computation requirements; this is the reason why *Neural Networks* is the technique selected for this protocol. The graphs for this analytical technique and protocol are found in Figure 13.

#### 5.2.5. Abdomen Protocol

Regarding the Abdomen protocol, there is also an excellent linear correlation between CTDI_VOL_ and SSDE (R^2^ = 0.858). *Gaussian Process Regression* is the technique that best minimizes the RMSE in both training data and test data when 100% of the samples are analyzed. On the contrary, the worst performance is reached with the *SVR* and *linear regression* models.

**Figure 14:**
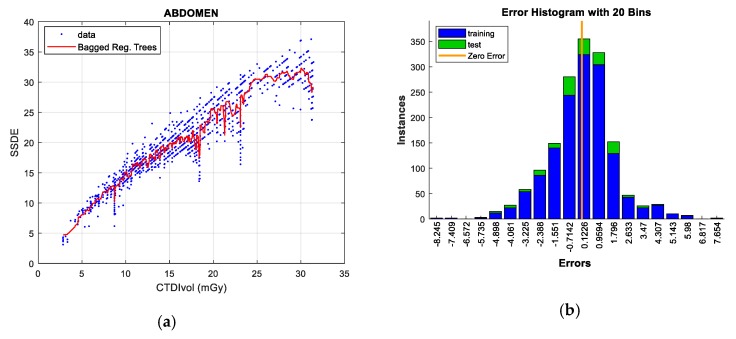
SSDE prediction according to CTDI_VOL_ for Abdomen protocol (**left**), together with error histograms (**right**), the ‘*bagged*’ *regression trees* technique (**a**,**b**).

This protocol presents very few atypical data, although of great magnitude, as can be observed in Table 18. Once they are removed, the RMSE value is significantly reduced (around 16.5%) and the *bagged regression trees* technique obtains the best predictions for the CTDI_VO_L-SSDE pair, followed by the *GPR* technique (however, as in the previous scenarios, its computational cost is the highest). On the other hand, *linear regression* and *SVR* exhibit the worst performance. Figure 14 shows the prediction and error graphs for this technique and protocol.

## 6. Discussion

The body mass index (BMI) is a metric that depends on weight and height, and it is therefore intrinsically related to the size of the patient. If the body is smaller than the volume of the standardized phantom (16/32 cm), the dose absorbed by the patient will be greater. In the same way, a larger body will receive less radiation.

Consequently, it is necessary to adapt the dose radiated by the CT to the size of the patient to comply with the recommended dose in a determined protocol and obtain a comprehensible image for the radiologist. Under these circumstances, we demonstrate an increase in the CTDI_VOL_ metric as BMI grows, which is consistent with: (i) the previous explanation, and (ii) the results extracted from the work [6]. In addition, there is a linear correlation between both figures of merit, which is emphasized if the outliers are removed.

The relationship between BMI and DRL for the different protocols should be highlighted. Firstly, figures for the ’Skull’ protocol illustrate that BMI-CTDI_VOL_ does not exceed the dose of any of the European reference levels included in this study. This is due to the fact that the size of the head does not affect the variance in the body mass index in the same way as the rest of the body. Secondly, in the protocols that are related to ‘Thorax, Abdomen, Pelvis’ and ‘Abdomen, Pelvis’, the regression curve usually remains below most of the DRLs. They only exceed specific DRLs when the body mass index rises above the value of 30, as is the case with obese patients. Thirdly, in the ‘Thorax’ protocol, the results show that a few DRL values are exceeded in the case of a BMI metric higher than 25. However, in usual operation conditions, the regression curve remains below most DRLs for most of the BMI values.

Concerning the regression curves for each protocol, they provide very valuable information to the radiologist to establish the appropriate dose value for radiating the patient. Thanks to this set of ML tools, the radiologist knows the radiation thresholds that must not be exceeded beforehand. However, as observed in Figure 8 and Figure 9, these thresholds can be surpassed in specific cases (for instance, when the calculation of the BMI value for a patient is high), and only with the most restrictive DRLs.

In contrast, in the ‘Abdomen’ protocol, we observe that the regression curves surpass some DRLs of European countries for BMI values that were included in normal weight ranges. In this case, the reduction of the radiated dose to the patient below the value indicated by the ML tool is a decision that depends on the clinical judgment of the radiologist, since he/she must be able to analyze and discern possible lesions in the CT images.

Regarding the CTDI_VOL_-SSDE predictions, we notice a high linear correlation between both of the variables. This means that a good SSDE prediction is achieved with extremely low error in most of the protocols. This prediction is clearly important, as it is possible to know with appropriate enough accuracy the dose that a patient will receive, without compromising the diagnosis, and while taking into account (i) his/her morphology and (ii) the output radiation value of the CT. Using this study, the radiologist can avoid situations in which a patient receives a higher dose than that required, simply by carrying out an appropriate adjustment to the CT parameters.

In relation to the RMSE metric, it is calculated from the study that was carried out for the different ML techniques. Under this study, an evident decrease of the RMSE is obtained when eliminating outliers, only reducing the error by more than 50% in some cases by discarding less than 5% of atypical data. This result also has a twofold meaning. On the one hand, a high RMSE value is attained for the atypical data, in comparison with these same samples when they are trained without these atypical data. On the other hand, greater error is observed (independently of having outliers or not) in the predictions of the Skull protocol when particularizing the protocols studied here. This is due to the fact that the data are more dispersed than in the rest of the protocols and cannot be adjusted in the same range.

The following factors have been considered and analyzed to select the appropriate ML regression technique: RMSE, overfitting, and computational complexity. Table 19 summarizes this study, highlighting the techniques that provide a better adjustment to the data; that is, focusing on *Neural Networks*, *regression trees,* and *Gaussian processes*. These will be discussed below.

The *bagged regression trees* technique provides a quick interpretation of the results due to its simplicity. In addition to its low computational cost, it also performs the best adjustment of the training data in most cases, obtaining the lowest RMSE values. However, *bagged regression trees* shows a high discrepancy when comparing the training results with their corresponding test data, which indicates the existence of a certain overfitting or lack of generality. Additionally, the figures of error histograms corroborates the overfitting phenomenon; big test bars (which include a greater number of data) point to higher error than their respective training data. On the other hand, this technique does not draw a curve with smooth transition, but it undergoes quick variations due to the overfitting. This means that two values near to the predictor variable result in different responses. For example, two patients with similar BMI values are radiated with different doses. Furthermore, this variability can cause peaks in the graph that exceed the DRL values in specific points.

*Neural Networks* is a powerful tool that, with appropriate data selection for the training tasks, allows for us to achieve good test data adjustment. Furthermore, this technique is able to enhance the results that were obtained by other techniques, although not for all the protocols. Its main drawbacks lie in: (i) the optimal selection of parameters, such as the number of layers and neurons, (ii) the high variability in the training results, and (iii) the unpredictability in the values of initialization of the weights, entailing different results in each execution of the network. This means that the model has to be repeatedly trained for each configuration = to select the iteration that offers the best performance. Once selected, it is necessary to store the value of the network weights to replicate the attained results. The execution time of this technique depends, to a great extent, on the number of layers and neurons used in the multilayer architecture.

In many of the protocols under study, the ML technique that presents the best performance is *Gaussian Process Regression* (*GPR*), due to the low RMSE obtained and the slight differences between the predictions of training data and test data. Moreover, since this is a probabilistic model, it is easy to calculate confidence intervals, which are of interest when establishing the thresholds of radiated doses to patients. The disadvantage is its algorithmic complexity, which implies longer processing and execution time, but this is fully acceptable for current computers.

Finally, once the ML curves are obtained for each protocol/gender, the medical staff must proceed, as follows:

(i) Regarding the patient’s disease, the staff selects a protocol/gender.
(ii) Table 4, Table 5, Table 6, Table 7, Table 8, Table 9, Table 10, Table 11, Table 12, Table 13, Table 14, Table 15, Table 16, Table 17 and Table 18 provide the best RMSE result in Test for the selected protocol/gender, and, therefore, the most appropriate ML technique.
(iii) The medical staff will go to the corresponding Figure defined for the dupla protocol and ML technique, and according to the patient’s morphology/size, they will take a BMI (or SSDE) value as input to obtain the new value of CTDI_VOL_.
(iv) In the future, the goal is for this new value of CTDI_VOL_ to be configured into the CT. To achieve this, X-ray technicians (or automated software) should tune input magnitudes, such as pitch, scan length, amperage, and kilovoltage.

## 7. Conclusions

In this paper, we contribute a novel methodology based on Machine Learning Techniques to estimate and predict the dose that is received by a patient in a CT test. To achieve this goal, the figures BMI and CTDI_VOL_, in a first stage, and CTDI_VOL_ and SSDE, in a second one, are studied and analyzed for five standardized protocols.

We obtain regression curves employing ML techniques regarding CTDI_VOL_-BMI_,_ and once the outliers are removed from a dataset composed of the data from over fifty thousand doses belonging to real patients. They draw the future dose to radiate and remain below most of the European DRLs for all of the protocols analyzed, except in Abdomen, where our predictions exceed the DRLs of restricted countries for normal BMI values. According to CTDI_VOL_–SSDE prediction, similar results were attained, with techniques, such as *Gaussian processes*, *Neural Networks*, and *Regression trees* showing an appreciable adjustment with the source of data computed, implying a high correlation coefficient and a very small RMSE; even less than one unit in some protocols.

As a result, our proposed predictive method provides a reliable and powerful tool for planning the dose to deliver to the appropriate patient. Those in the medical field will have useful information when deciding to adjust the dose or not, which minimizes the impact of the radiation in treated patients.

## Figures and Tables

**Table 1:** Technical information on each computed axial tomography (CT) used in this work.

Manufacturer	Model	# Slices	Enabled SSDE	Hospital	# CT
GE	LightSpeed VCT	64	YES	A	CT1
TOSHIBA	Aquilion	16	YES	A	CT2
SIEMENS	Somaton Def. AS	64	YES	B	CT3
SIEMENS	Somaton D. AS+	128	YES	B	CT4
GE	LightSpeed VCT	64	YES	C	CT5
SIEMENS	Somaton Emotion	16	NO	C	CT6
SIEMENS	Somaton Emotion	16	NO	D	CT7
SIEMENS	Somaton Def. AS	64	NO	D	CT8
PHILIPS	Brilliance	6	NO	E	CT9
PHILIPS	Brilliance	16	NO	E	CT10
PHILIPS	Brilliance	16	NO	F	CT11
GE	LightSpeed VCT	64	YES	G	CT12
SIEMENS	Somaton Emotion	16	NO	H	CT13

**Table 2:** Exam distribution by protocol and gender.

Protocol	2015–2016	2017 Men	2017 Women	TOTAL
Head	27,785	1380	1210	30,375
Thorax	3817	769	534	5120
Abdomen	2221	541	407	3169
Abdomen & Pelvis	7518	1459	1495	10,472
Thorax, Abdomen & Pelvis	6288	1808	1339	9435

**Table 3:** Machine Learning (ML) Technique parameters.

***Linear Regression***	- Linear Model (y_i_ = β_0_ + β_1_x_i_,). In Each Protocol/Gender, β_0_ and β_1_ Parameters are Calculated - Type of Fit: Least Squares Fit
***Regression Trees***	- Minimum number of data in non-terminal nodes: 10 - Minimum number of data in terminal nodes: 10 (in ’Skull’ protocol it increases to 20). - Pruning criterion: MSE - Data splitting criterion: MSE - Number of trees in bagging: 50
***Gaussian Process***	- Base function: constant H = 1 (vector of nx1 dimension, with n = number of data). - Kernel function: exponential.
***Support Vector Regression***	- Kernel function: the radial basis function (non-linear case). High σ values involve overfitting. We have selected the range from 2 to 700 for our probes
***Neural Networks***	- Number of hidden layers: 1 - Number of neurons in the hidden layer: 10 in the ’Skull’ protocol, and 5 in the rest of the protocols. - Training function: Bayesian regularization. - Normalization of entries between -1 and 1. - Activation function of neurons in the output layer: linear. - Activation function of neurons in the hidden layer: sigmoid (hyperbolic tangent).

**Table 4:** BMI-CTDI_VOL_ results for men’s Skull protocol.

ML Technique	RMSE 100% of the Sample		RMSE without Outliers (96.4% of the Sample)		RMSE of Outliers	Execution Time (seconds)
	Train	Test	Train	Test		
L. Regression	6.6477	6.6255	5.6307	5.6248	2.4774e + 03	3.519739
Reg. Trees	5.8796	6.5221	5.0302	5.7588	334.8542	2.997568
B.R. Trees	5.7889	6.4314	4.9609	5.6237	337.4054	7.712964
*GPR*	6.3956	6.4565	5.5735	5.6148	337.6461	221.628132
*SVR*	6.5762	6.5937	5.6921	5.7273	362.0229	5.588844
**N. Networks**	**6.4782**	**6.4620**	**5.6038**	**5.6090**	**350.0536**	**25.188203**

**Table 5:** BMI-CTDI_VOL_ results for women’s Skull protocol.

ML Technique	RMSE 100% of the Sample		RMSE without Outliers (96.4% of the Sample)		RMSE of Outliers	Execution Time (seconds)
	Train	Test	Train	Test		
L. Regression	5.6609	5.6433	4.5959	4.6015	5.3330e + 03	15.052260
Reg. Trees	5.1083	5.7975	4.0426	4.6288	299.4519	8.581264
B.R. Trees	5.0274	5.6543	3.9596	4.5134	305.4321	11.127855
*GPR*	5.6008	5.5970	4.5444	4.5997	287.4341	241.964651
*SVR*	5.6552	5.6282	4.5980	4.6413	298.0206	7.459742
**N. Networks**	**5.6618**	**5.6253**	**4.4972**	**4.5007**	**414.3626**	**22.069401**

**Table 6:** BMI-CTDI_VOL_ results for men’s Thorax, Abdomen, & Pelvis protocol.

ML Technique	RMSE 100% of the Sample		RMSE without Outliers (96.4% of the Sample)		RMSE of Outliers	Execution Time (seconds)
	Train	Test	Train	Test		
L. Regression	7.9476	7.3695	2.5205	2.5191	2.3180e+ 03	12.030910
Reg. Trees	6.8448	7.0617	2.2935	2.5824	109.6120	5.921255
B.R. Trees	6.6967	6.8954	2.2436	2.5236	109.4708	10.666126
**GPR**	**6.8744**	**6.7515**	**2.4770**	**2.5012**	**103.3175**	**662.439870**
*SVR*	7.5910	6.8664	2.6011	2.6087	127.5686	8.584487
N. Networks	7.4479	6.7736	2.4934	2.5101	111.8788	37.235825

**Table 7:** BMI-CTDI_VOL_ results for women’s Thorax, Abdomen & Pelvis protocol.

ML Technique	RMSE 100% of the Sample		RMSE without Outliers (96.4% of the Sample)		RMSE of Outliers	Execution Time (seconds)
	Train	Test	Train	Test		
L. Regression	5.9616	5.6679	2.7319	2.7267	2.0834e+ 04	11.728024
Reg. Trees	4.9465	5.1369	2.4740	2.8359	102.8771	5.660822
B.R. Trees	4.8739	5.0179	2.4240	2.7422	100.4587	9.794929
*GPR*	5.2814	4.8710	2.6728	2.7110	96.1594	405.771978
*SVR*	5.6819	5.1555	2.8080	2.8172	106.1870	706.658904
**N. Networks**	**5.4569**	**4.9517**	**2.7116**	**2.7100**	**131.6909**	**56.300129**

**Table 8:** BMI-CTDI_VOL_ results for men’s Abdomen & Pelvis protocol.

ML Technique	RMSE 100% of the Sample		RMSE without Outliers (96.4% of the Sample)		RMSE of Outliers	Execution Time (seconds)
	Train	Test	Train	Test		
L. Regression	5.1519	5.1360	3.5519	3.5503	6.7896e + 03	12.091683
Reg. Trees	3.4529	4.0113	2.9921	3.4408	155.5703	5.835483
**B.R. Trees**	**3.3633**	**3.8846**	**2.9314**	**3.3445**	**155.1968**	**10.629959**
*GPR*	3.2541	3.9459	3.2776	3.4362	135.4733	604.821809
*SVR*	4.0721	4.0942	3.5810	3.5962	167.3552	7.908900
N. Networks	3.9910	4.0642	3.4884	3.4976	206.1475	46.168296

**Table 9:** BMI-CTDI_VOL_ results for women’s Abdomen & Pelvis protocol.

ML Technique	RMSE 100% of the Sample		RMSE without Outliers (96.4% of the Sample)		RMSE of Outliers	Execution Time (seconds)
	Train	Test	Train	Test		
L. Regression	5.9153	5.8852	3.8414	3.8354	1.4838e + 04	11.816178
Reg. Trees	4.0523	4.6282	3.4111	3.9066	230.8406	6.023535
B.R. Trees	3.9650	4.5395	3.3391	3.8323	232.3786	10.536164
*GPR*	4.1726	4.4879	3.7311	3.8129	225.2929	367.542711
*SVR*	4.5277	4.5195	3.8213	3.8595	270.7293	7.318787
**N. Networks**	**4.4462**	**4.4748**	**3.8099**	**3.8017**	**211.2679**	**40.271456**

**Table 10:** BMI-CTDI_VOL_ results for men’s Thorax protocol.

ML Technique	RMSE 100% of the Sample		RMSE without Outliers (96.4% of the Sample)		RMSE of Outliers	Execution Time (seconds)
	Train	Test	Train	Test		
L. Regression	6.0020	5.8699	3.8035	3.8021	2.6905e + 03	11.930498
Reg. Trees	4.7992	5.5382	3.4223	4.0329	302.0919	5.887889
B.R. Trees	4.7070	5.2697	3.3366	3.9207	293.1283	9.019784
*GPR*	5.1254	5.0927	3.7388	3.8073	288.1348	72.430923
*SVR*	5.5247	5.3443	4.0014	4.0149	333.5253	4.923137
**N. Networks**	**5.2823**	**5.4493**	**3.7865**	**3.7872**	**288.1060**	**24.706027**

**Table 11:** BMI-CTDI_VOL_ results for women’s Thorax protocol.

ML Technique	RMSE 100% of the Sample		RMSE without Outliers (96.4% of the Sample)		RMSE of Outliers	Execution Time (seconds)
	Train	Test	Train	Test		
L. Regression	5.2020	4.9851	2.5661	2.5648	2.8919e + 03	11.832442
Reg. Trees	3.8554	4.2185	2.2976	2.6749	259.4087	5.809703
B.R. Trees	3.7946	4.0839	2.2430	2.5762	262.3007	8.058130
***GPR***	**3.4868**	**4.0486**	**2.4429**	**2.5375**	**282.2755**	**64.182540**
*SVR*	4.1363	3.8822	2.5565	2.5769	325.4391	4.436024
N. Networks	3.9221	3.6765	2.5403	2.5425	270.0588	37.296176

**Table 12:** BMI-CTDI_VOL_ results for men’s Abdomen protocol.

ML Technique	RMSE 100% of the Sample		RMSE without Outliers (96.4% of the Sample)		RMSE of Outliers	Execution Time (seconds)
	Train	Test	Train	Test		
L. Regression	4.8270	4.8062	4.6266	4.6293	96.9090	11.731780
Reg. Trees	4.1740	4.8179	4.1062	4.7107	42.7575	5.474494
B.R. Trees	4.0679	4.6702	4.0042	4.6016	43.8989	7.996369
*GPR*	4.5152	4.6488	4.4453	4.5822	43.1798	51.105172
*SVR*	4.6965	4.7658	4.6238	4.6620	46.5827	4.095963
**N. Networks**	**4.6376**	**4.6413**	**4.5727**	**4.5790**	**47.0929**	**23.880534**

**Table 13:** BMI-CTDI_VOL_ results for women’s Abdomen protocol.

ML Technique	RMSE 100% of the Sample		RMSE without Outliers (96.4% of the Sample)		RMSE of Outliers	Execution Time (seconds)
	Train	Test	Train	Test		
L. Regression	6.3722	7.8462	4.2790	4.2982	2.5861e + 03	11.457573
Reg. Trees	4.1392	4.7517	3.7614	4.4513	118.2078	5.583748
B.R. Trees	4.0710	4.6160	3.6936	4.3245	116.2066	7.835361
***GPR***	**4.3666**	**4.5081**	**3.9618**	**4.2000**	**114.3182**	**49.429170**
*SVR*	4.5828	4.5606	4.1785	4.2685	163.9891	4.174639
N. Networks	4.5738	4.5695	4.1623	4.2013	108.6456	34.185516

**Table 14:** SSDE-CTDI_VOL_ results for Skull protocol.

ML Technique	RMSE 100% of the Sample		RMSE without Outliers (96.4% of the Sample)		RMSE of Outliers	Execution Time (seconds)
	Train	Test	Train	Test		
L. Regression	7.6581	7.6569	5.7078	5.7080	553.9211	11.195573
Reg. Trees	5.5046	5.7751	2.9325	3.1008	667.3612	6.007195
**B.R. Trees**	**5.4675**	**5.7535**	**2.9057**	**3.0545**	**657.6257**	**26.495838**
*GPR*	5.3103	5.7914	2.8654	3.0610	720.6968	4046.977135
*SVR*	6.5697	6.5967	4.0111	4.0164	681.0965	325.671414
N. Networks	5.7063	5.9609	3.0665	3.1766	2.4302e + 04	1953.304959

**Table 15:** SSDE-CTDI_VOL_ results for Thorax, Abdomen, & Pelvis protocol.

ML Technique	RMSE 100% of the Sample		RMSE without Outliers (96.4% of the Sample)		RMSE of Outliers	Execution Time (seconds)
	Train	Test	Train	Test		
L. Regression	2.1103	2.0521	1.2632	1.2622	153.4590	11.847310
Reg. Trees	2.1502	2.2710	1.0942	1.2590	475.5703	6.646121
B.R. Trees	2.1210	2.1960	1.0766	1.2375	476.6240	16.922205
*GPR*	1.6332	2.0351	1.1517	1.2033	490.9936	1286.947536
*SVR*	2.1161	2.0527	1.2652	1.2642	153.9839	18.901946
**N. Networks**	**1.8830**	**1.9180**	**1.1924**	**1.1959**	**746.5661**	**56.397724**

**Table 16:** SSDE-CTDI_VOL_ results for Abdomen & Pelvis protocol.

ML Technique	RMSE 100% of the Sample		RMSE without Outliers (96.4% of the Sample)		RMSE of Outliers	Execution Time (seconds)
	Train	Test	Train	Test		
L. Regression	3.2286	3.2123	1.5718	1.5722	6.6130	15.878153
Reg. Trees	2.9808	3.3122	1.3419	1.4863	16.8985	6.566288
B.R. Trees	2.9265	3.2423	1.3093	1.4440	16.3447	13.912800
***GPR***	**3.0796**	**3.2103**	**1.3169**	**1.4236**	**44.0821**	**1135.093285**
*SVR*	3.2483	3.2317	1.5757	1.5764	19.7135	17.352789
N. Networks	3.1718	3.2235	1.5453	1.5476	11.7811	98.081114

**Table 17:** SSDE-CTDI_VOL_ results for Thorax protocol.

ML Technique	RMSE 100% of the Sample		RMSE without Outliers (96.4% of the Sample)		RMSE of Outliers	Execution Time (seconds)
	Train	Test	Train	Test		
L. Regression	3.4305	2.8991	1.0152	1.0035	2.0958	11.788787
Reg. Trees	3.2398	3.2159	0.9064	1.0396	19.5033	5.443749
B.R. Trees	3.1717	3.1201	0.8890	1.0152	17.4512	9.093581
*GPR*	3.2415	2.7189	0.9284	0.9954	10.9758	181.903988
*SVR*	3.5216	3.0683	1.0120	1.0075	36.2798	4.656816
**N. Networks**	**3.3230**	**2.6779**	**0.9984**	**0.9945**	**11.5088**	**35.041588**

**Table 18:** SSDE-CTDI_VOL_ results for Abdomen protocol.

ML Technique	RMSE 100% of the Sample		RMSE without Outliers (96,4% of the Sample)		RMSE of Outliers	Execution Time (seconds)
	Train	Test	Train	Test		
L. Regression	2.6192	2.6188	2.5715	2.5715	109.3953	12.025513
Reg. Trees	2.2892	2.4613	1.8449	2.0374	967.4462	6.067233
**B.R. Trees**	**2.2542**	**2.4065**	**1.8219**	**2.0099**	**939.3754**	**9.511561**
*GPR*	1.9424	2.1055	1.9001	2.0340	1.2453e + 03	499.697872
*SVR*	2.7301	2.8274	2.3670	2.3716	1.5475e + 03	7.047249
N. Networks	2.2923	2.3278	2.1810	2.2010	846.7025	22.795797

**Table 19:** Comparison among ML Techniques.

ML Technique	Less RMSE	Less Overfitting	Less Complexity
*Linear regression*	X	V	V
*Regression trees*	X	X	V
*Bagged Regression trees*	V	X	V
*Gaussian Process Regression*	V	V	X
SVM Regression (*SVR*)	X	V	V
*Neural Networks*	V	V	X

## Supplementary Materials

The following are available online at https://www.mdpi.com/1424-8220/19/23/5116/s1, Supplementary File 1: Description of Machine Learning Techniques and Supplementary File 2: Results involving ML, protocols, and dose metrics.
