# Supplementary material for: Machine Learning Techniques Applied to Dose Prediction in Computed Tomography Tests
Source: Sensors (Basel). 2019 Nov 22;19(23):5116. doi: 10.3390/s19235116 (PMC6928694; doi:10.3390/s19235116)
Supplement: Supplementary file 1 [file sensors-19-05116-s001.pdf]

Supplementary File 1: Description of Machine Learning Techniques

Machine learning (ML) algorithms, as a subfield of artificial intelligence (AI), have been providing effective solutions in engineering applications and to scientific problems for many decades. ML methods have the ability to adapt to new conditions and to detect and estimate patterns. Recently, ML has gained well-deserved attention in many fields of engineering and science, mostly due to the development of high-performance visualization applications. Many leading technology companies are heavily investing in AI/ML. Academia is following suit in order to develop more powerful algorithms which will be able to run efficiently on new emerging hardware. In addition to these recent developments, practitioners have found new ways to apply the many existing machine learning algorithms in their respective domains.

Machine Learning involves using self-learning algorithms to derive knowledge from data in order to carry out system predictions. To build models that analyze a large amount of data, ML provides a suitable solution that captures the knowledge present in data and gradually enhances the performance of predictive models. The main goal is to make the best decisions, or to take the best actions based on these predictions. Figure 1 shows a roadmap for building these ML models. Thanks to machine learning, we enjoy robust e-mail spam filters, convenient text and voice recognition software, reliable Web search engines, challenging chess opponents, economic concerns (e.g. analysis of stock market behavior), and hopefully, soon, safe and efficient self-driving cars.

ML is divided into three categories: supervised learning, unsupervised learning, and reinforcement learning. In this work we will focus our attention on supervised learning techniques since they allow a model to learn from training data to make predictions about unseen or future data. In supervised learning the input data is defined by labels (such as, for instance, mail labels) or raw data. One of the subcategories of supervised learning is regression analysis, which addresses the prediction of continuous results from labels/raw data. Given a set of variables x, named predictors, and their corresponding response variables y, we can fit a curve graph (the simplest is a straight line) to these data that minimizes the distance between the sample points and the fitted linear/non-linear graph. Observing the nature of the data to analyze in this work, the set unsupervised learning and regression analysis is adjusted to the requirements, allowing us to predict the continuous outcomes of target variables.

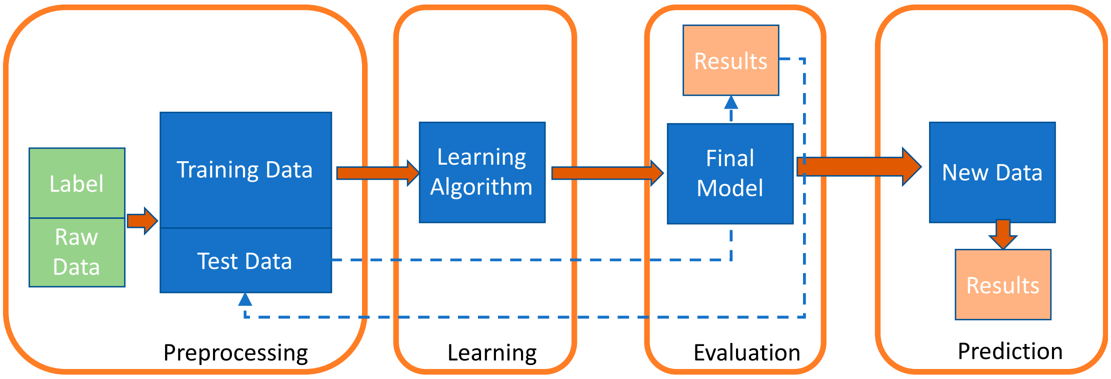

Figure S1. ML Model design

In regression analysis techniques, the scientific literature presents different approaches which are useful in the massive analysis of data (Big Data). Furthermore, these techniques help in the forecasting of future doses to be received by patients, which is a distinctive objective of this work.

Regarding regression analysis models, we will concentrate our efforts on specifying (data selection), accommodating (eliminating outliers and anomalous points), and analyzing our large amount of CT exam data by using the following models:

1. *Linear regression*. This technique consists of finding a line that fits a data set following a certain criterion. The most common criterion, which will also be employed in this work, is least squares adjustment [1].

2. *Decision Tree Learning*. This scheme breaks down our data by making decisions based on asking a series of questions. In particular, in the training phase, the decision tree model learns questions used to stamp class labels on the samples. As a tree model, the process starts at the root of the tree and splits the data along its branches. The splitting procedure is repeated at each child node up to the leaves (of the tree). This means that the samples of each node belong to the same class. Note that error is minimized if the tree is deep, but it can lead to overfitting. Thus, the usual procedure is to prune the tree, restricting its maximum depth. A better way to improve the results of the *Decision Tree Learning* algorithm is to employ a technique called *Bagged Decision Tree,* which reduces the variance of a decision tree.

3. *Bagged Decision Tree*. In this technique, multiple *regression trees* are constructed. In particular, several subsets of data are created from training samples, for each collection of them to be later used to train their own decision trees. The average derived from these different decision trees provides a more robust solution than a single decision tree. The use of several trees also reduces overfitting.

4. *Artificial Neural Networks* [2]. Our focus will be on analyzing data for the training phase with a technique called *Bayesian regularization* [3]. Figure 2 shows the scheme of blocks accomplished for this work. This algorithm allows us to perform binary classification, and we will use the *Levenberg-Marquardt optimization* [4] to learn the weight coefficients of the model (in each iteration of the training phase, the coefficients are updated). Furthermore, it is possible to obtain the optimal weights employing cost functions such as those called *Sum of Squared Errors* (SSE). Finally, $y^{(i)}$ is the target of a particular sample $x^{(i)}$, and $a^{(i)}$ is the activation of the neuron, which is a linear function. We define the activation function φ (·) as φ (z) = z = a. In this regard, the net input function is a linear combination of the weights that connect the input to the output layer: z=$\sum w_j x_j = w^T x$. To find more result states, including predicted values, the solution involves connecting multiple single neurons to a *multi-layer feedforward neural network*. This particular type of network is also called a *multi-layer perceptron (MLP)*, which consists of three layers: the input layer, the hidden layer, and the output layer. The units in the hidden layer are fully connected to the input layer, and the output layer is fully linked to the hidden layer, respectively. Both techniques (*Bayesian regularization* and *Levenberg-Marquardt* optimization), together with an infrastructure MLP achieve an optimal model capable of generalizing the mathematical problem thanks to the minimization of a combination of weights and errors. This algorithm allows overfitting to be reduced at the cost of longer execution time.

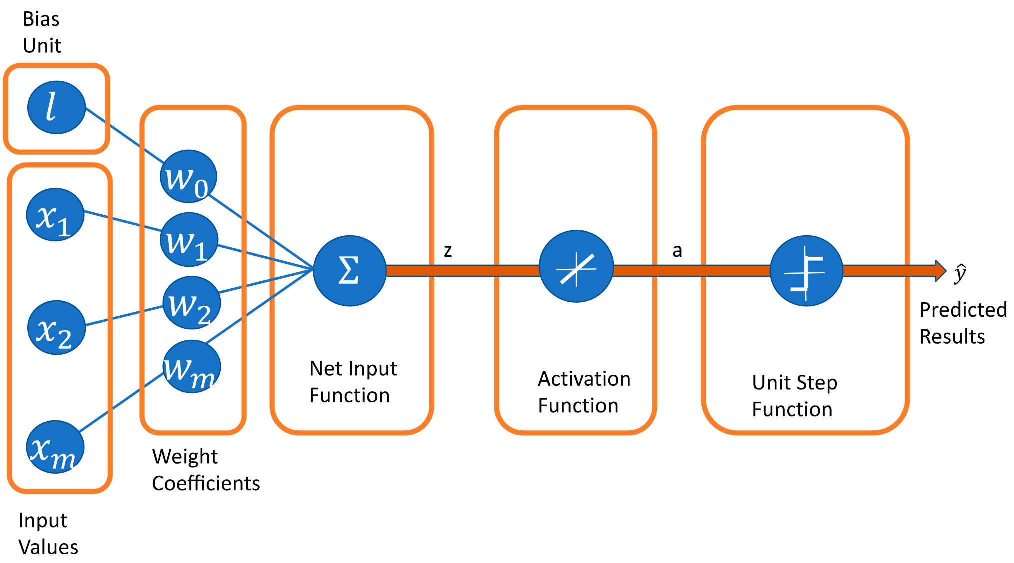

Figure S2. *Artificial Neural Networks* scheme employed for this work

5. *Gaussian Process Regression* [5]. Parametric regression methods, for instance linear/logistic regression, generate a line or a curve in the graph of inputs and outputs, replacing the training data. So, once the regression weights have been obtained, the original training data may be eliminated from the graph. On the other hand, non-parametric regression methods may retain the initial training data (also called latent variables) to be used as a significant element in generating a regressor function. To this end, test data is compared to the training data points; each output value of the test point is estimated via the distance of the test data input to the training data input. It is notable that non-parametric regression considers that data points with similar input values will be close in output space. Mathematical expressions include the covariance function formed of latent variables, which reflects the smoothness of the response.

   Covariance and *mean functions* were used in conjunction with a *Gaussian likelihood* for prediction with the following equations (all them have been derived in [4]):

$$f_*|X, y, X_* \backsim N(\overline{f}_*, cov(f_*)), \quad (1)$$

   Where $\boldsymbol{f}_*$ is a posterior distribution, X is a matrix of training inputs, y is a vector of training target, $\boldsymbol{X}_*$ is a matrix of test inputs, $\overline{\boldsymbol{f}}_*$ is the posterior mean, and $\boldsymbol{cov}(\boldsymbol{f}_*)$ is the posterior covariance.

$$\overline{f}_* = K(X_*, X)[K(X, X) + \sigma_n^2 I]^{-1} \quad (2)$$

$$cov(f_*) = K(X_*, X_*) - K(X_*, X)[K(X, X) + \sigma_n^2 I]^{-1} K(X, X_*), \quad (3)$$

   Where K indicates a covariance matrix, and $\boldsymbol{\sigma_n^2}$ is a noise variance term.

   On the other hand, $\boldsymbol{N}(\overline{\boldsymbol{f}}_*, \boldsymbol{cov}(\boldsymbol{f}_*))$ also can be defined as:

$$N(yh|H\beta + f, \sigma_n^2 I) \quad (4)$$

   Where H is a set of base functions which transform the original vector x into a new one h(x), and $\boldsymbol{\beta}$ is the coefficient vector of the base function, yh is the result of the traditional *linear*

regression $= \boldsymbol{x^T\beta} + \boldsymbol{\varepsilon}$, being $\boldsymbol{\varepsilon}$ the error calculated as $\boldsymbol{\varepsilon} \sim \boldsymbol{N}\,(\boldsymbol{0}, \boldsymbol{\sigma_n^2})$, and f($x_i$) are real values for each x_i.

Therefore, to accomplish suitable predictions, it is necessary to obtain the $\boldsymbol{\beta}$ coefficients belong to the base function, the noise variance $\boldsymbol{\sigma_n^2}$ and the selected kernel hyperparameters to calculate the covariance matrix. To estimate these parameters, we employ the work [6] to maximize $\boldsymbol{f_*|X,y,X_*}$ in function of $\boldsymbol{\beta}, \boldsymbol{\sigma_n^2}$ and the kernel hyperparameters. This prediction method requires a high computational cost in order of O(kn3), where *k* is the number of evaluations to fulfill the maximization problem and *n* is the number of data.

In this work, we employ the following exponential function as a kernel function:

$$k_{exp}(x,x') = \sigma_f^2 \exp\left(-\sqrt{\sum_{k=1}^{d} \frac{(x_{i,k} - x_{j,k})^2}{l_k^2}} \right) \qquad (5)$$

Where the parameter $\boldsymbol{\sigma_f^2}$ is the standard deviation, while l_k is the scalar dimension for each predictor, and x and x' are two near values.

6. *Support Vector Regression* (*SVR*) [7]. In this case, we consider the following training data$\{(\boldsymbol{x_i},\boldsymbol{y_i}), \ldots\ldots, (\boldsymbol{x_l},\boldsymbol{y_l})\}$, where $\boldsymbol{x_i} \subset \boldsymbol{R^n}$; $\boldsymbol{y_i} \subset \boldsymbol{R}$ indicate the input space of the sample and its corresponding target value, respectively, and *l* denotes the size of the training data. Our objective is to find a function $\boldsymbol{f(x)}$ that has, at most, ε deviation from the obtained targets $\boldsymbol{y_i}$ for all the training data and at the same time, is as flat as possible. In other words, we do not care about errors because they are less than ε, that is, any deviation which is higher than ε will be allowed. Additionally, to find a function $\boldsymbol{f(x)}$ that returns the best fit, the results must avoid senseless predictions.

   Regarding the relationship between $\boldsymbol{x}$ and $\boldsymbol{y}$, it is approximately linear, which means that the model is represented as: $\boldsymbol{f(x)} = \langle \boldsymbol{w},\boldsymbol{x}\rangle + \boldsymbol{b}$; $\boldsymbol{w} \subset \boldsymbol{R^n}$; $\boldsymbol{b} \subset \boldsymbol{R}$ (*w* represents coefficients, while the *b* term is an intercept). Therefore, this problem can be formulated as a convex optimization problem:

$$minimize\ \frac{1}{2}\|w\|^2$$

$$subject\ to \begin{cases} y_i - \langle w, x_i\rangle - b \le \varepsilon \\ \langle w, x_i\rangle + b - y_i \le \varepsilon \end{cases}$$

   This optimization problem could be feasible or not, or simply a non-linear case. All of them are analyzed in the following Tables 1 and 2:

Table S1. ***SVR* linear case**

Feasible solution: $\boldsymbol{f(x)}$ exists since it approximates all pairs $(\boldsymbol{x_i},\boldsymbol{y_i})$ with ε precision	*Non-feasible solution.* Soft Margin functions adapted to *SVR* by [9], introducing slack variables $\boldsymbol{\xi_i}, \boldsymbol{\xi_i^*}$ to deal with unfeasible constrains
$\boldsymbol{minimize\ \frac{1}{2}\|w\|^2}$	$\boldsymbol{minimize\ \frac{1}{2}\|w\|^2 + C\sum_{i=1}^{l} \xi_i + \xi_i^*}$

$$subject\ to \begin{cases} y_i - \langle w, x_i \rangle - b \le \varepsilon \\ \langle w, x_i \rangle + b - y_i \le \varepsilon \end{cases}$$	$$subject\ to \begin{cases} y_i - \langle w, x_i \rangle - b \le \varepsilon + \xi_i \\ \langle w, x_i \rangle + b - y_i \le \varepsilon + \xi_i^* \\ \xi_i, \xi_i^* \ge 0 \end{cases}$$ The constant $C > 0$ determines the trade-off between the flatness (this means that one seeks a small w value) of f and the amount up to which deviations larger than ε are tolerated.
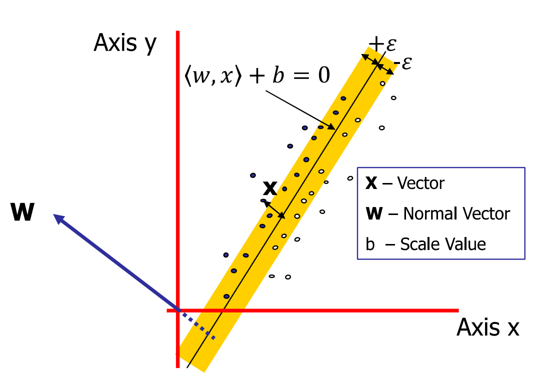	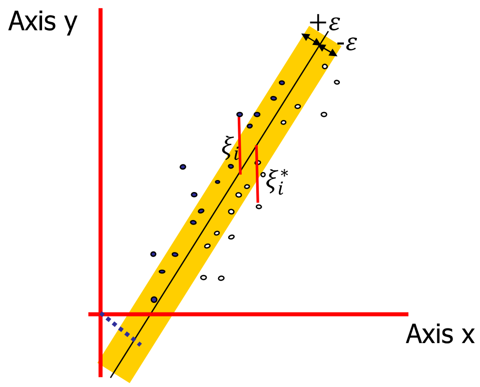

Table S2. *SVR* non-linear case

**Non-linear case**	
Primal	Dual
$$minimize\ \frac{1}{2}\|w\|^2 + C\sum_{i=1}^{l} \xi_i + \xi_i^*$$ $$subject\ to \begin{cases} y_i - \langle w, \varphi(x_i) \rangle - b \le \varepsilon + \xi_i \\ \langle w, \varphi(x_i) \rangle + b - y_i \le \varepsilon + \xi_i^* \\ \xi_i, \xi_i^* \ge 0 \end{cases}$$	$$max \left\{ \frac{1}{2} \sum_{i=1}^{l} (\alpha_i - \alpha_i^*)(\alpha_j - \alpha_j^*) \langle \varphi(x_i), \varphi(x_j) \rangle - \varepsilon \sum_{i=1}^{l} (\alpha_i + \alpha_i^*) + \sum_{i=1}^{l} y_i (\alpha_i - \alpha_i^*) \right.$$ $$s.t. \sum_{i=1}^{l} (\alpha_i - \alpha_i^*) = 0\ ; 0 \le \alpha_i, \alpha_i^* \le C$$ $\langle \varphi(x_i), \varphi(x_j) \rangle = K(x_i, y_i)$ is a Kernel function (e.g. a common kernel used for this model is the called radial basis function: $K(x_i, y_i) = e^{-\frac{\|xi - yi\|^2}{2\sigma^2}}$

References

1. Huaan Fan. *Theory of errors and least squares adjustment*. Royal Institute of Technology (KTH). 2010
2. Sebastian, R.; Vahid, M. *Python Machine Learning*; 2017; Packt Publishing; United Kingdom; ISBN-13 9781787125933.
3. Burden, F.; Winkler, D. Bayesian regularization of neural networks. *Methods Mol. Biol.* **2008**, 23–42, doi: 10.1007/978-1-60327-101-1_3.
4. Levenberg-Marquardt algorithm. Available online: https://en.wikipedia.org/wiki/Levenberg%E2%80%93Marquardt_algorithm (accessed on 28 April 2019)
5. Caywood Matthew S.; Roberts Daniel M.; Colombe Jeffrey B.; Greenwald Hal S.; Weiland Monica Z. Gaussian Process Regression for Predictive but Interpretable Machine Learning Models: An Example of Predicting Mental Workload across Tasks. *Front. Hum. Neurosci*. **2017**, *10*, 647.
6. Rasmussen, C. E; Williams, C. K. I. *Gaussian processes* for Machine Learning. The MIT Press: Cambridge, MA, USA, 2005.
7. Cortes, C; Vapnik, V. Support vector networks. *Mach. Learn*. **1995**, *20,* 273–297.
8. Smola, A.J.; Schölkopf, B. *Statistics and Computing;* John Wiley & Sons Ltd.: Chichester, UK, 2004.

Supplementary File 2: Results involving ML, protocols, and dose metrics

1. Comparison between CTDIvol and BMI

1.1. Men's Skull Protocol

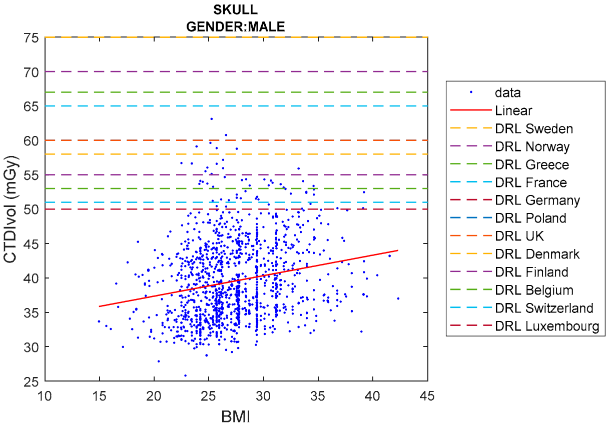

(a)

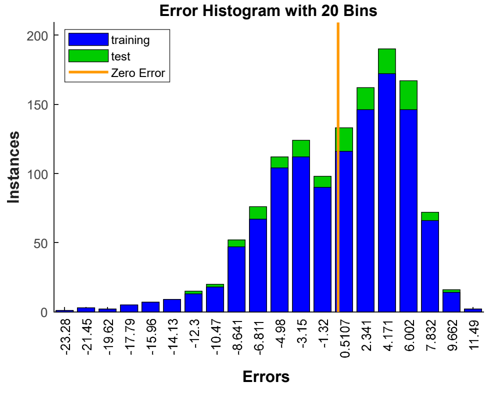

(b)

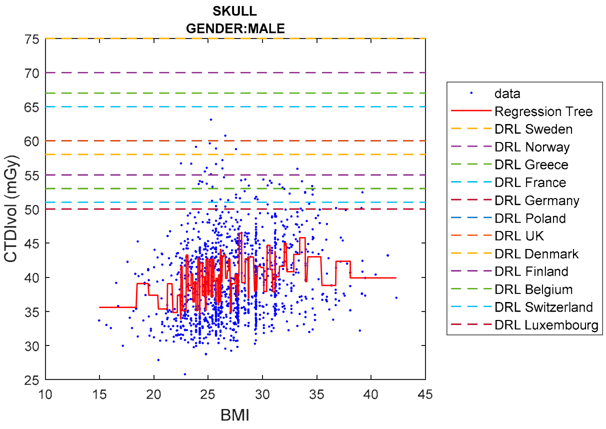

(c)

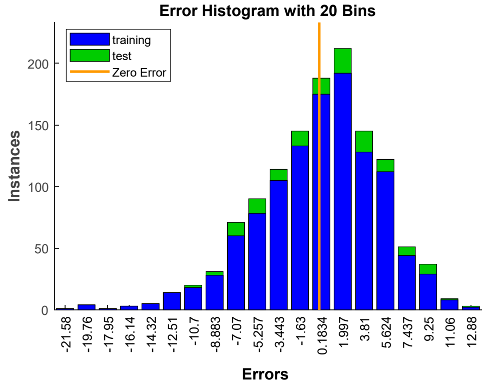

(d)

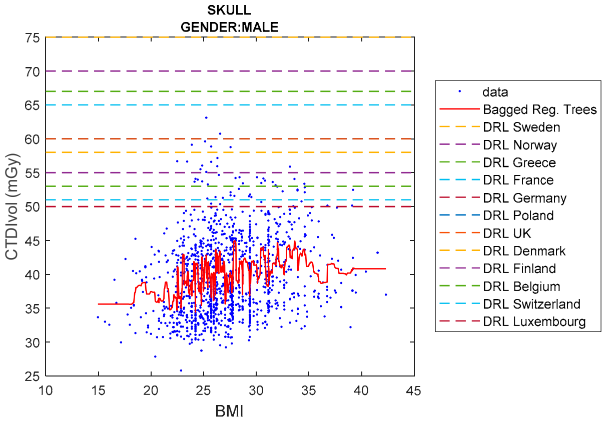

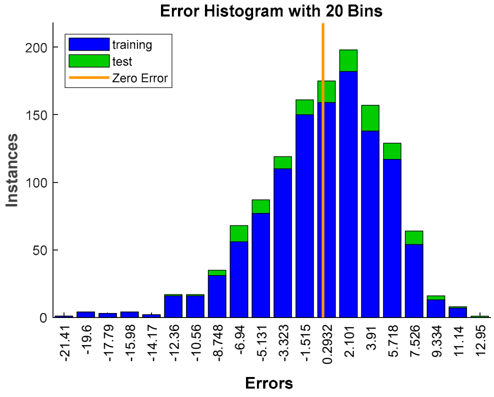

(e)

(f)

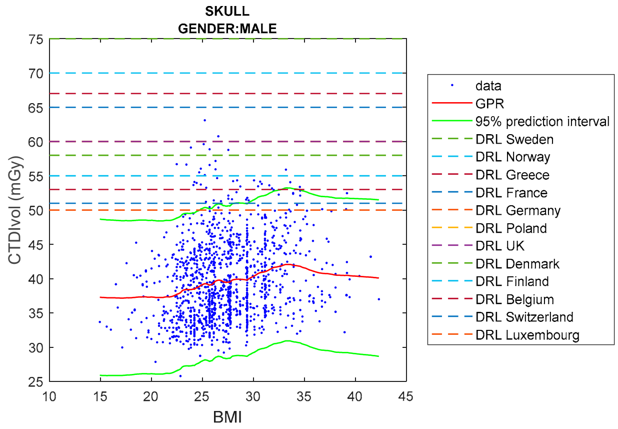

(g)

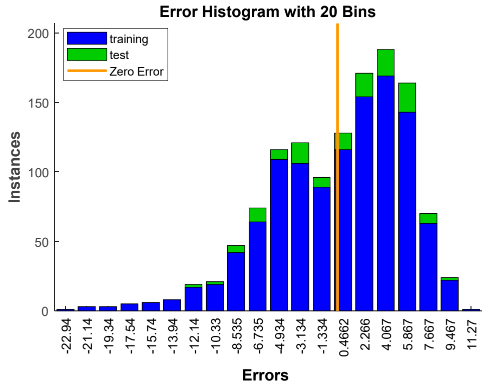

(h)

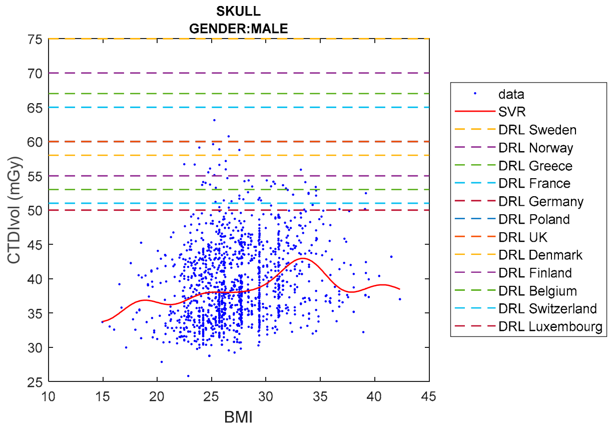

(i)

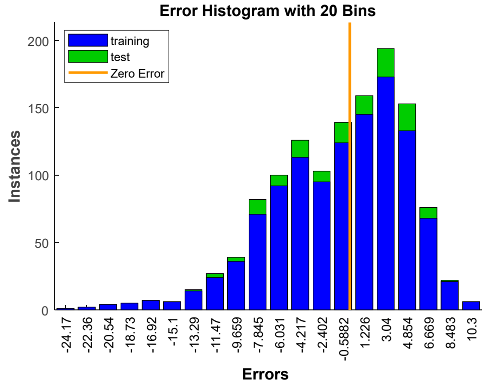

(j)

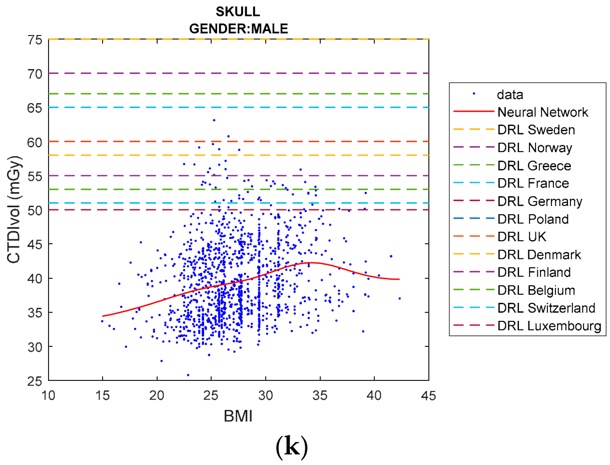

(k)

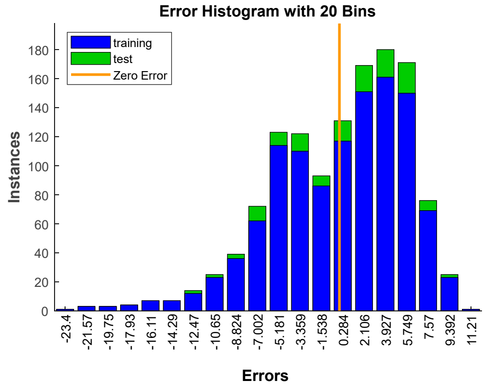

(l)

Figure S3. CTDIVOL prediction according to BMI including European DRLs for men's skull protocol (left), together with error histograms (right), employing the following ML techniques: *linear regression* (a) and (b), *regression trees* (c) and (d), '*bagged*' *regression trees* (e) and (f), *GPR* (g) and (h), *SVR* (i) and (j), and *neural networks* (k) and (l).

1.2. Women's Skull Protocol

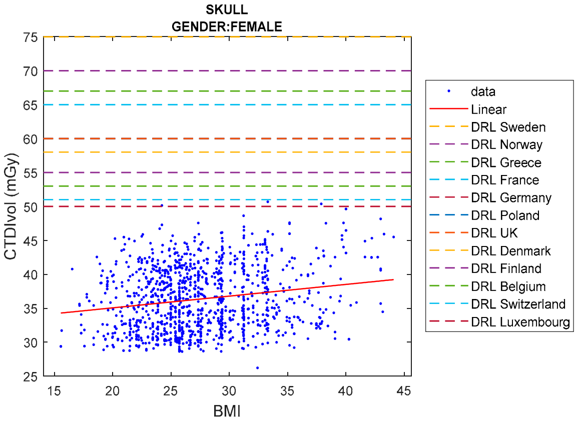

(**a**)

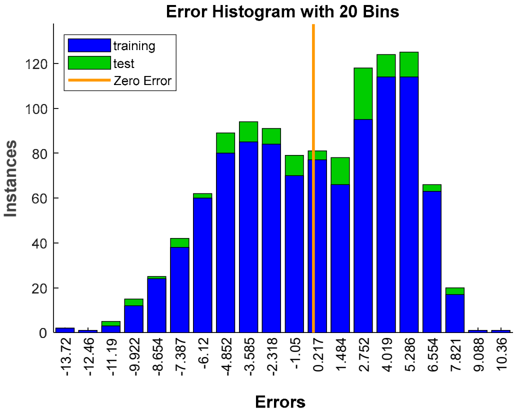

(**b**)

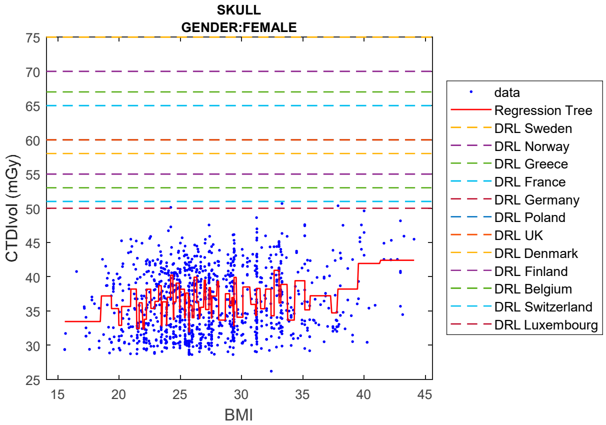

(**c**)

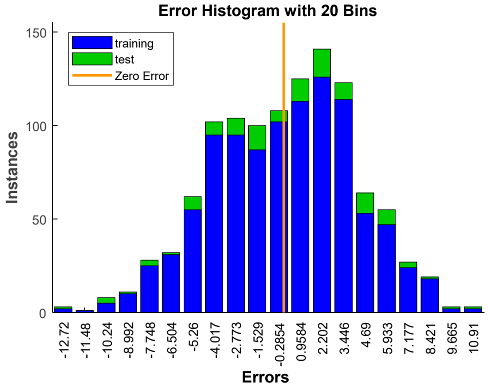

(**d**)

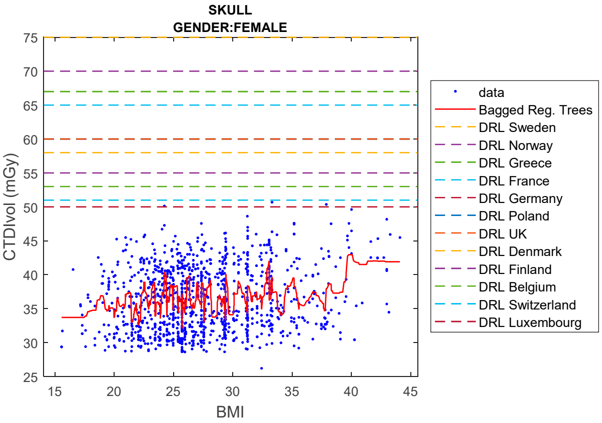

(**e**)

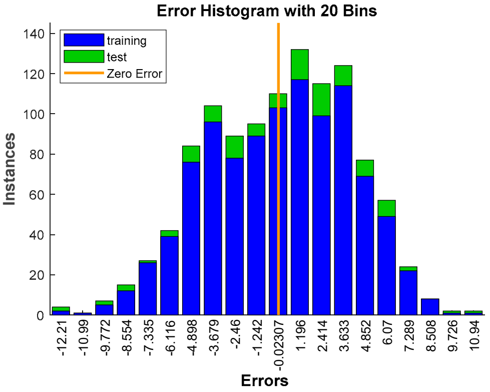

(**f**)

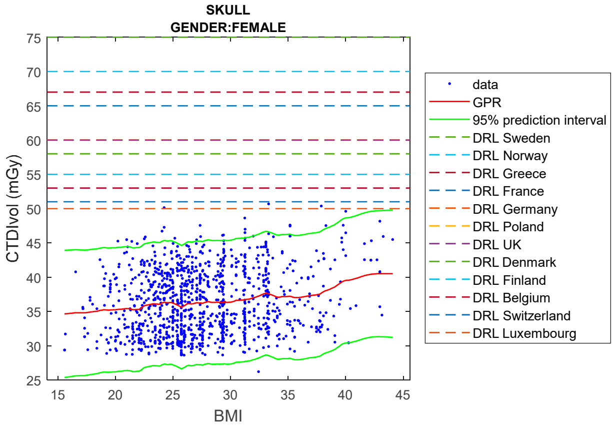

(g)

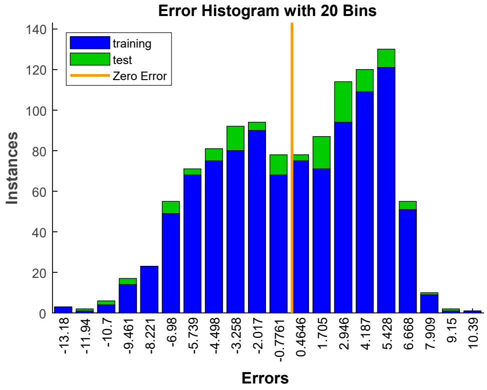

(h)

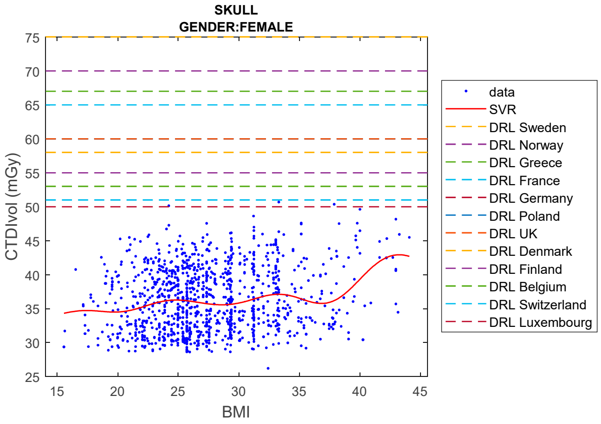

(i)

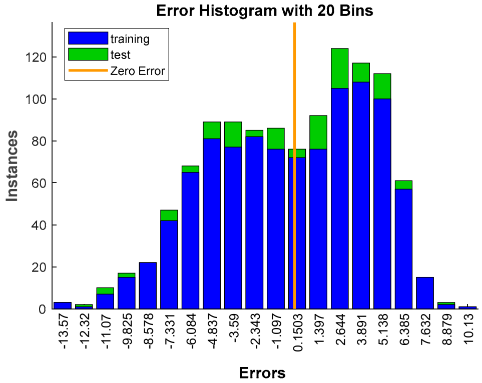

(j)

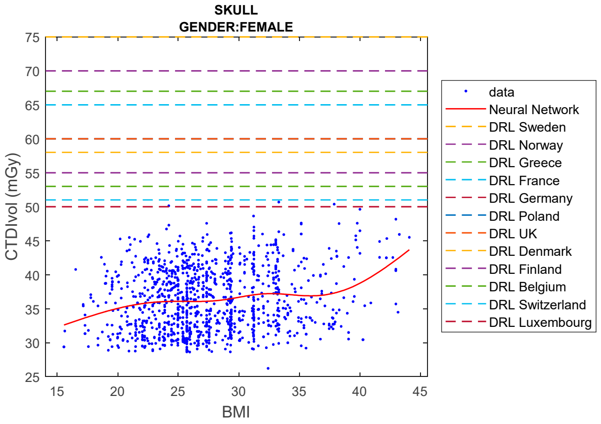

(k)

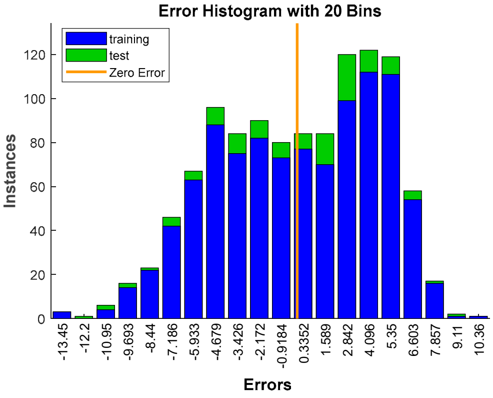

(l)

Figure S4. $CTDI_{VOL}$ prediction according to BMI including European DRLs for women's skull protocol (left), together with error histograms (right), employing the following ML techniques: *linear regression* (a) and (b), *regression trees* (c) and (d), *'bagged' regression trees* (e) and (f), *GPR* (g) and (h), *SVR* (i) and (j) and *neural networks* (k) and (l).

1.3. Men's Thorax, Abdomen and Pelvis Protocol

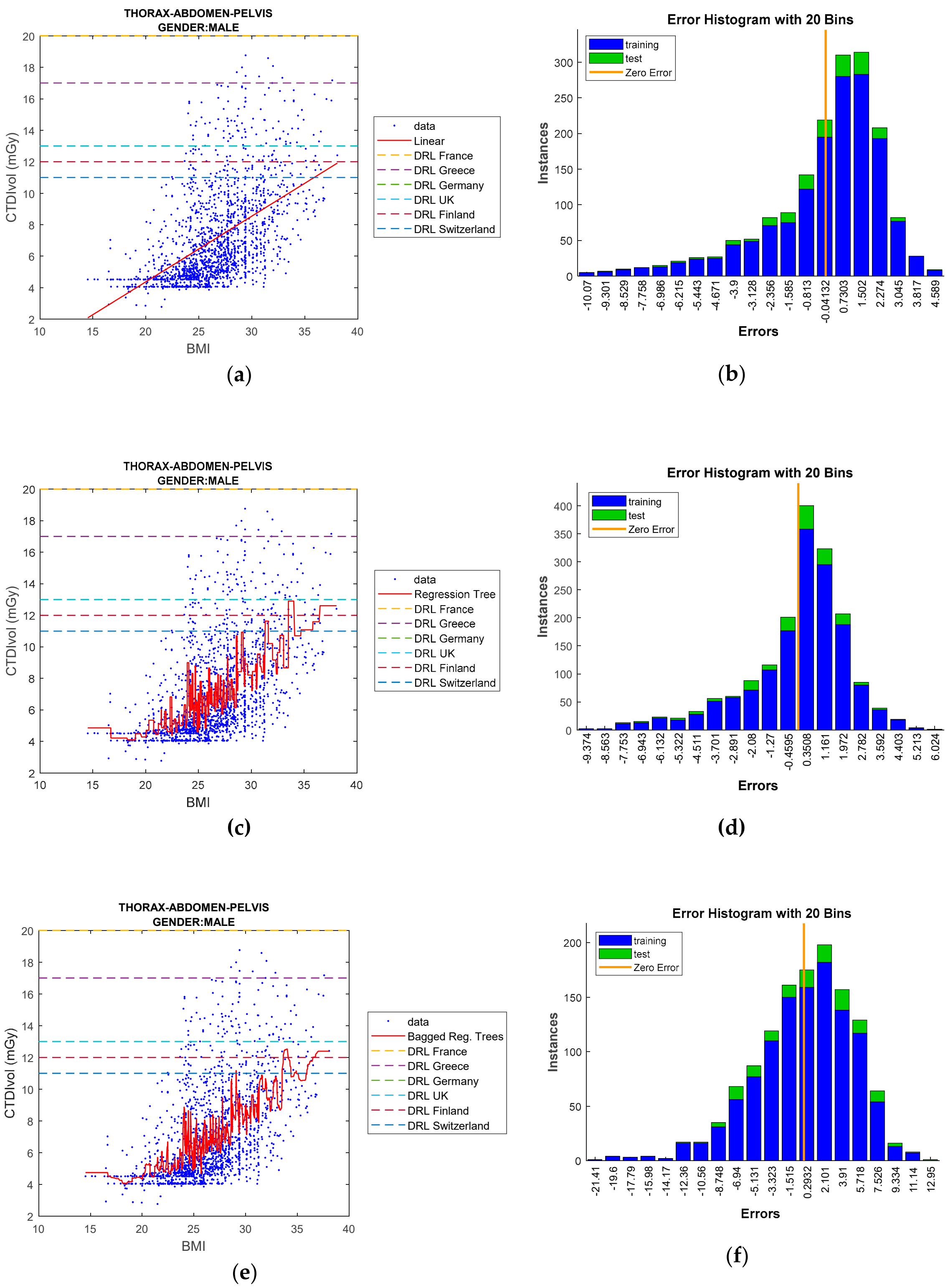

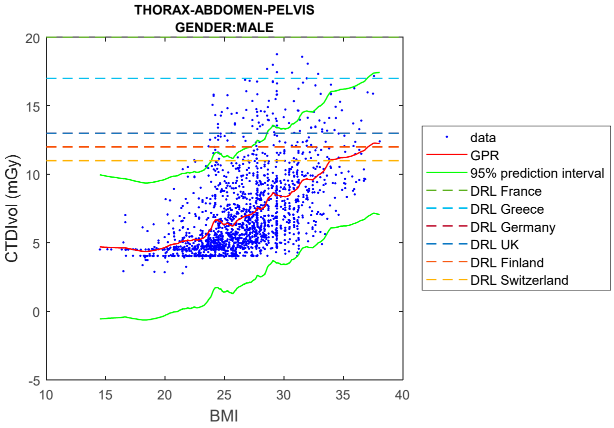

(g)

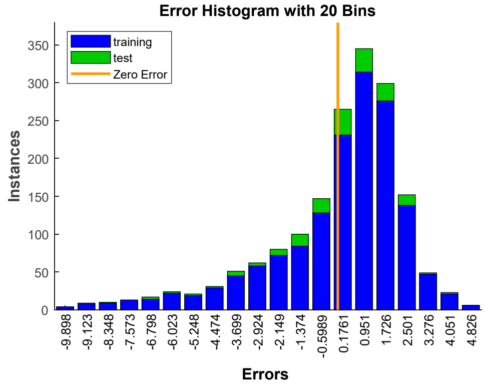

(h)

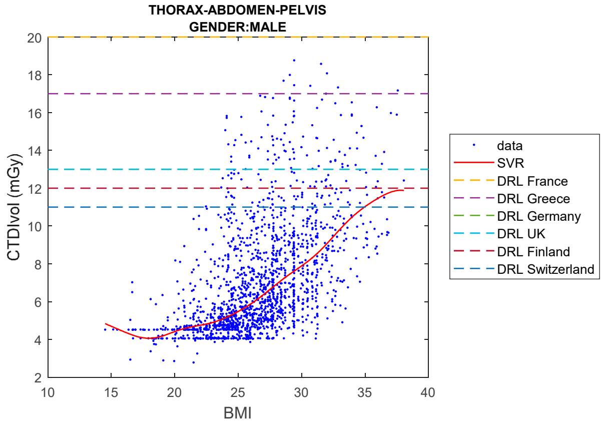

(i)

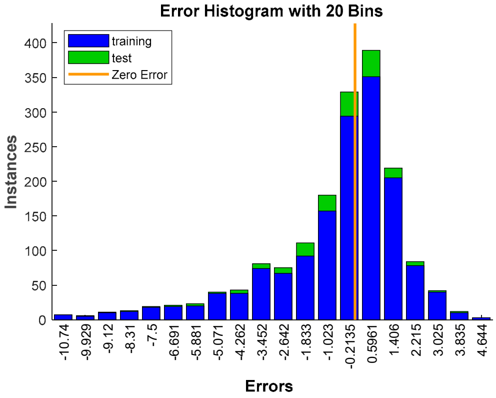

(j)

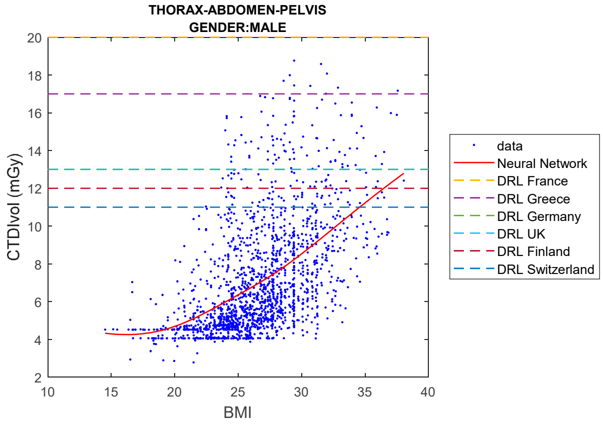

(k)

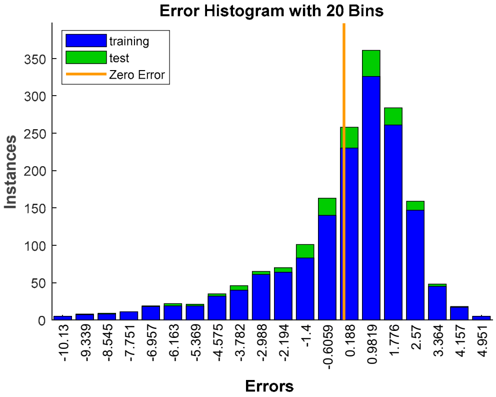

(l)

Figure S5. CTDIvol prediction according to BMI including European DRLs for men's thorax, abdomen & pelvis protocol (left), together with error histograms (right), employing the following ML techniques: *linear regression* (a) and (b), *regression trees* (c) and (d), *'bagged' regression trees* (e) and (f), *GPR* (g) and (h), *SVR* (i) and (j) and *neural networks* (k) and (l).

1.4. Women's Thorax, Abdomen and Pelvis Protocol

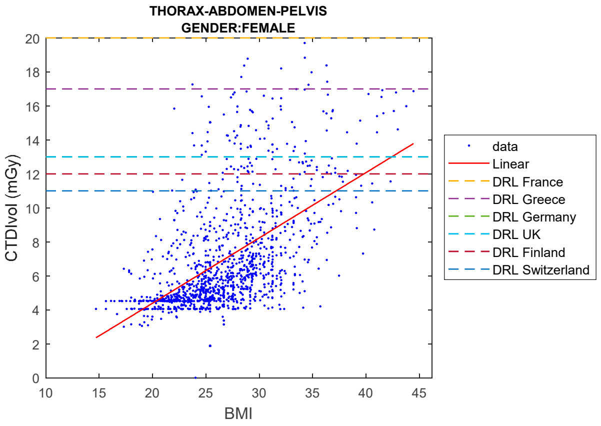

(a)

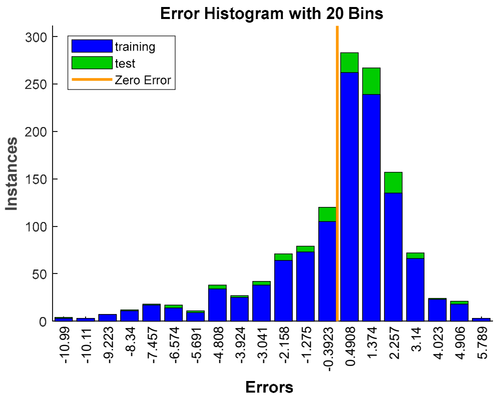

(b)

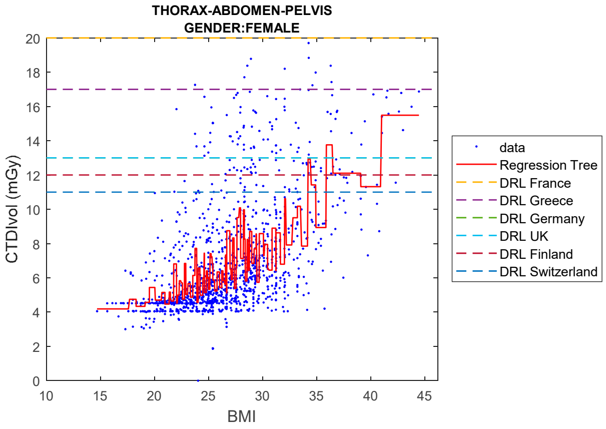

(c)

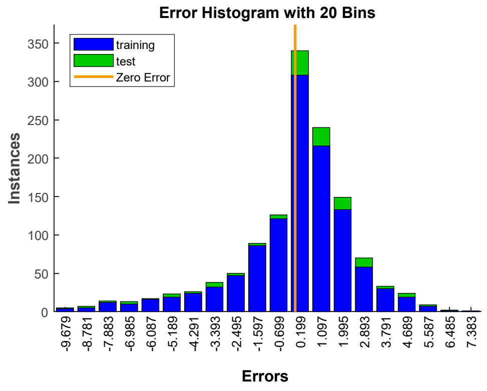

(d)

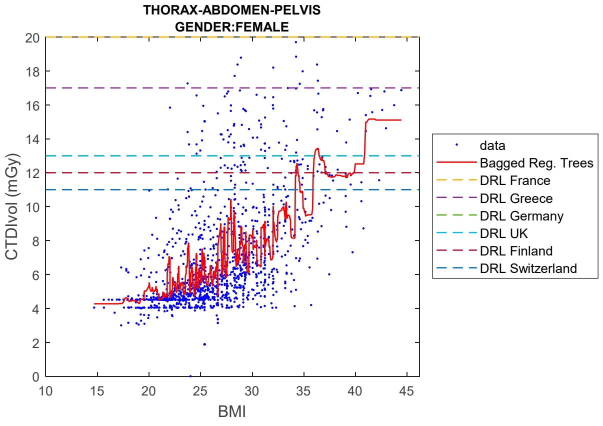

(e)

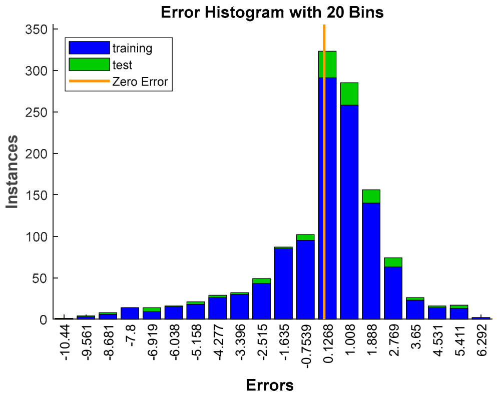

(f)

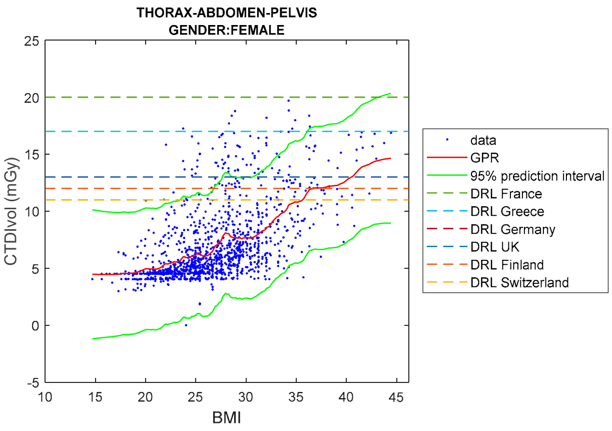

(g)

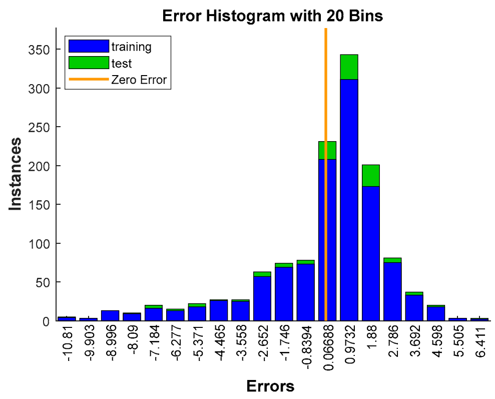

(h)

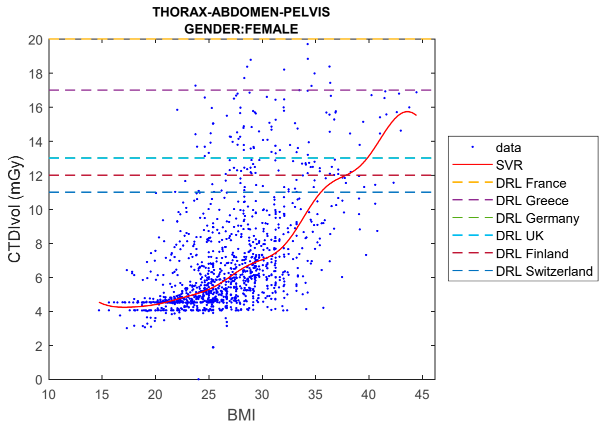

(i)

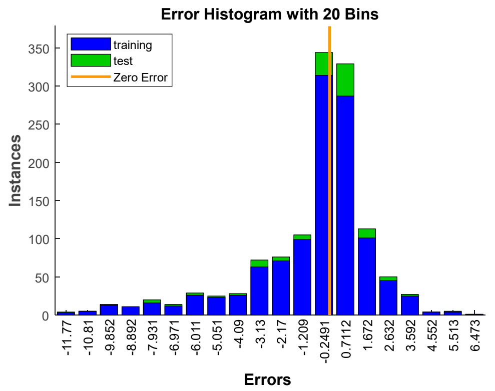

(j)

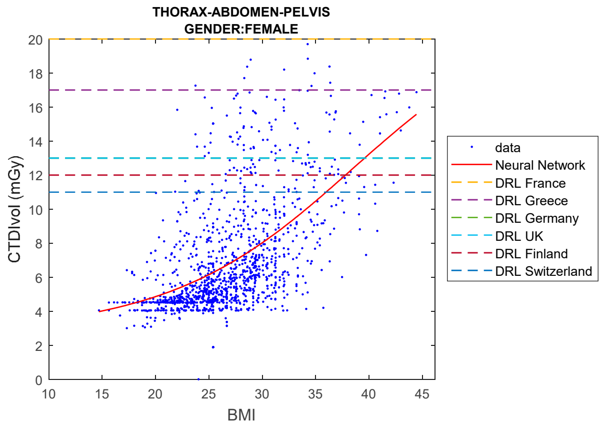

(k)

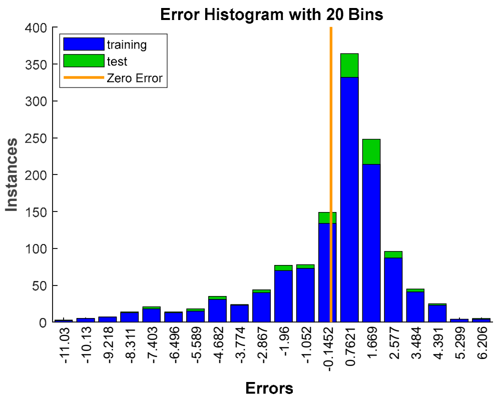

(l)

Figure S6. CTDIvol prediction according to BMI including European DRLs for women's Thorax, Abdomen & Pelvis protocol (left), together with error histograms (right), employing the following ML techniques: *linear regression* (a) and (b), *regression trees* (c) and (d), *'bagged' regression trees* (e) and (f), *GPR* (g) and (h), *SVR* (i) and (j) and *neural networks* (k) and (l) .

1.5. Men's Abdomen and Pelvis Protocol

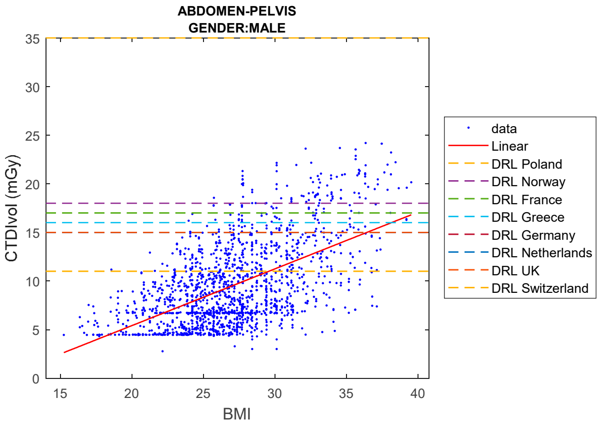

(**a**)

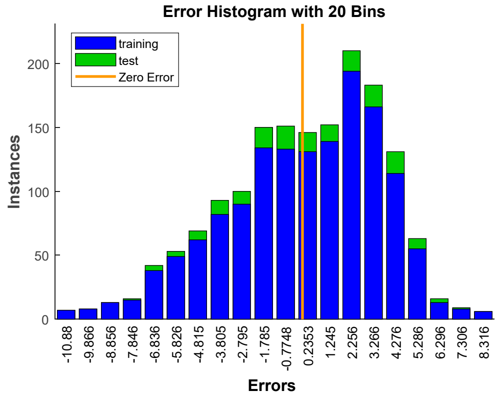

(**b**)

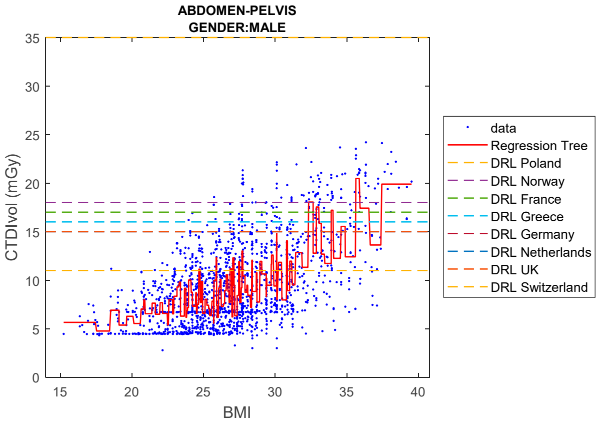

(**c**)

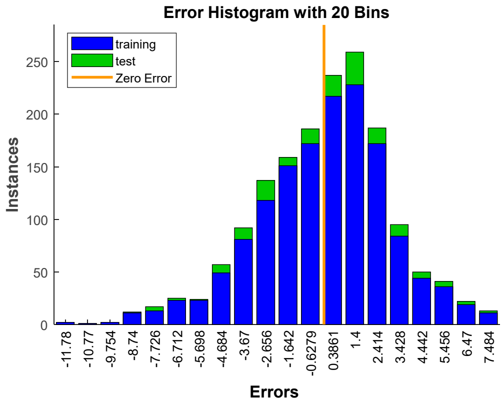

(**d**)

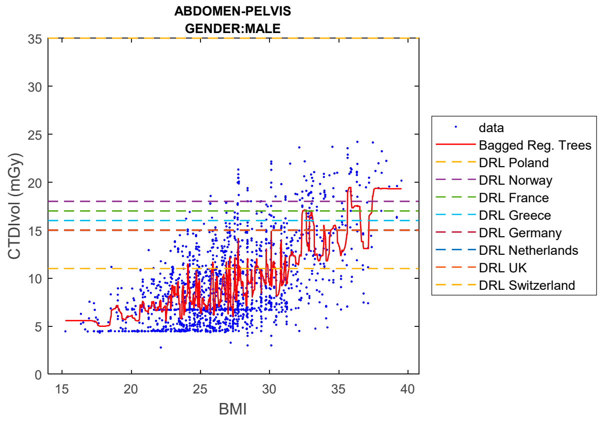

(**e**)

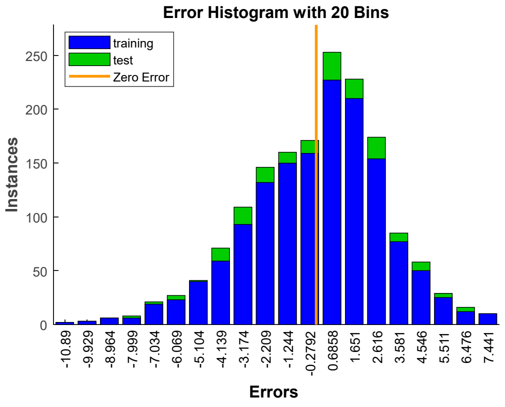

(**f**)

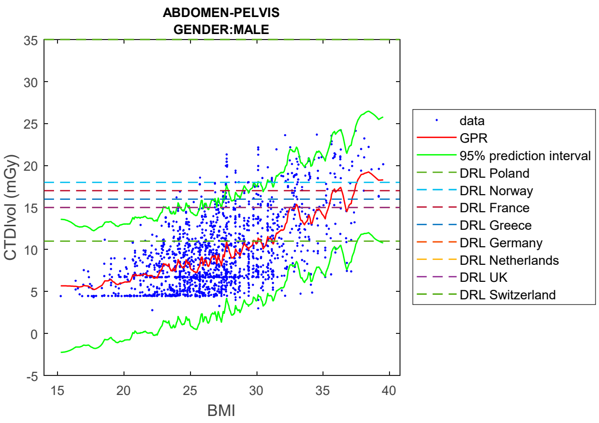

(g)

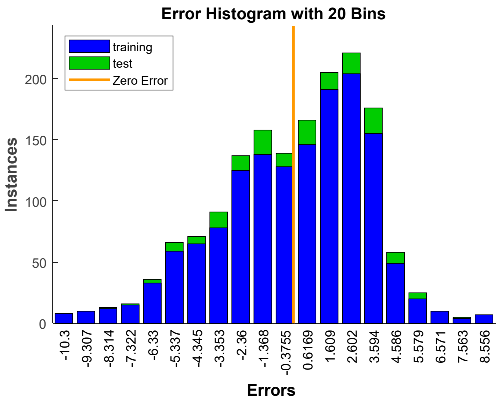

(h)

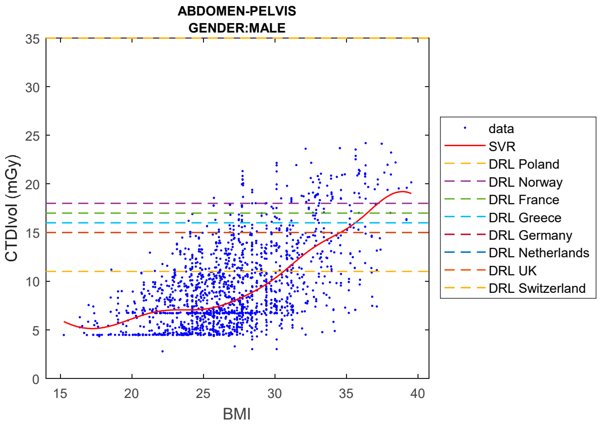

(i)

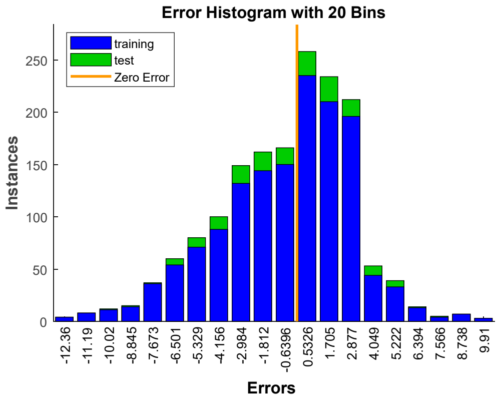

(j)

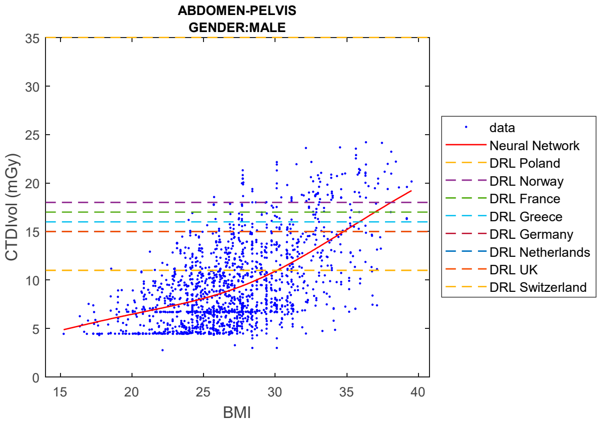

(k)

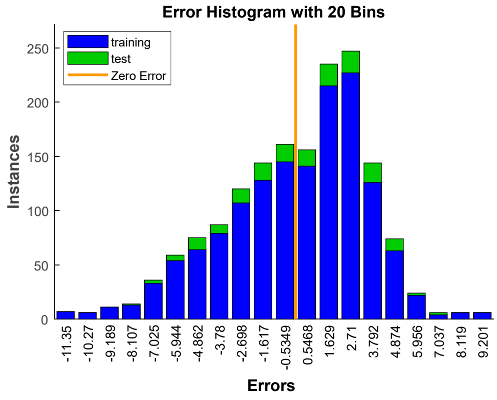

(l)

Figure S7. CTDI$_{\text{VOL}}$ prediction according to BMI including European DRLs for men's abdomen & pelvis protocol (left), together with error histograms (right), employing the following ML techniques: *linear regression* (a) and (b), *regression trees* (c) and (d), '*bagged*' *regression trees* (e) and (f), *GPR* (g) and (h), *SVR* (i) and (j) and *neural networks* (k) and (l).

1.6. Women's Abdomen and Pelvis Protocol

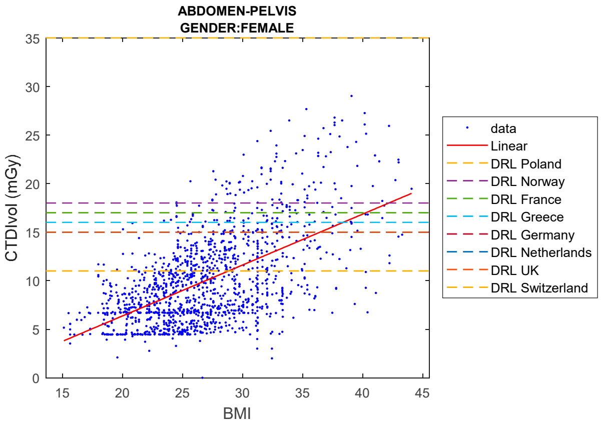

(**a**)

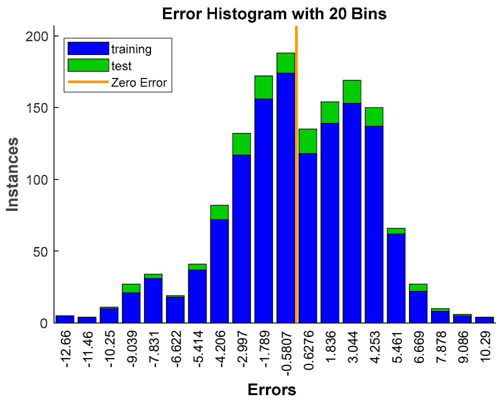

(**b**)

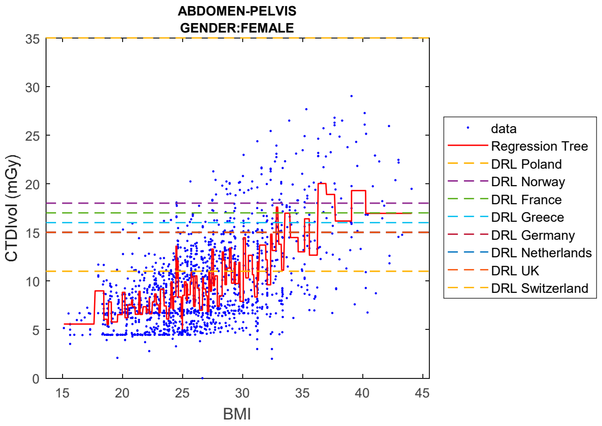

(**c**)

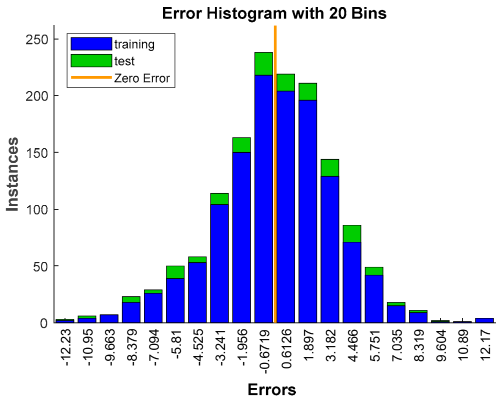

(**d**)

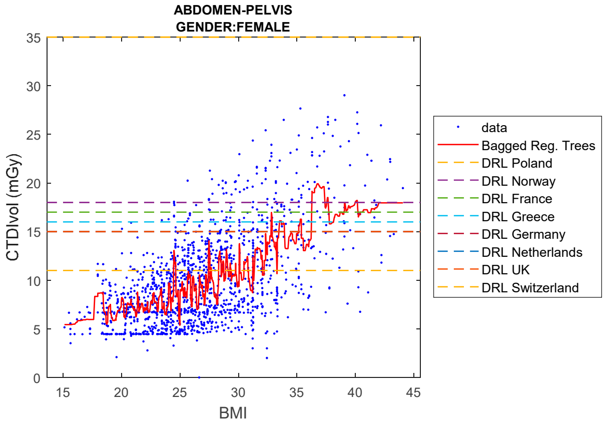

(**e**)

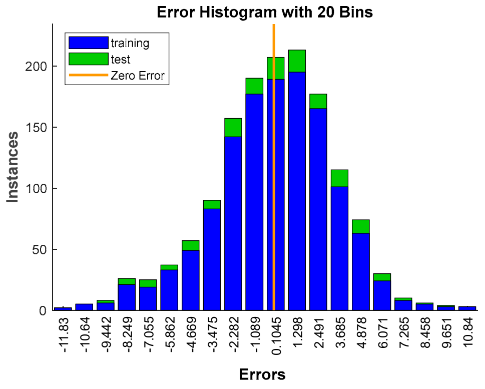

(**f**)

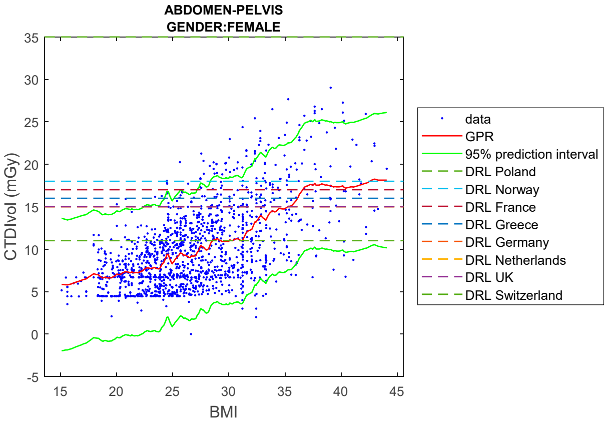

(g)

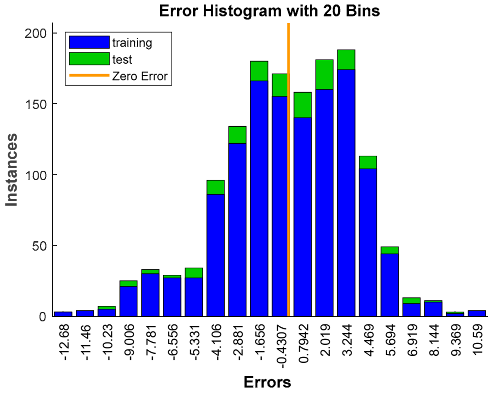

(h)

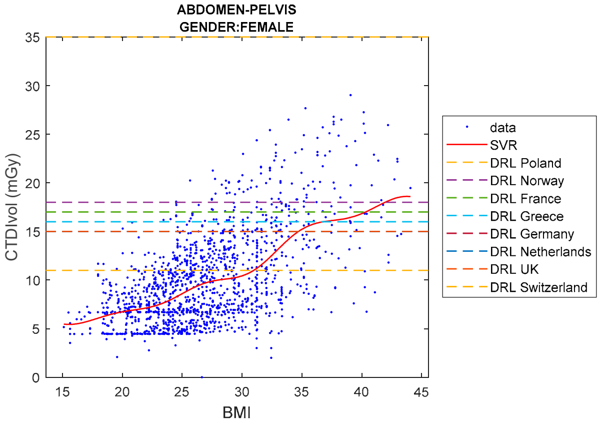

(i)

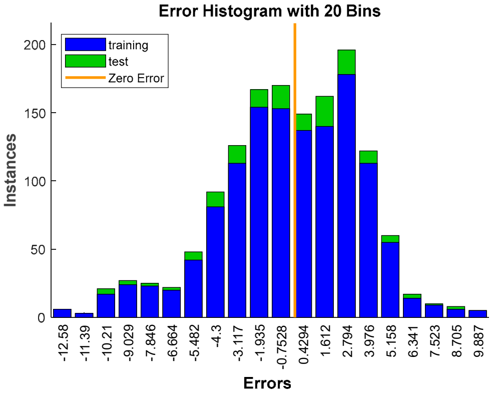

(j)

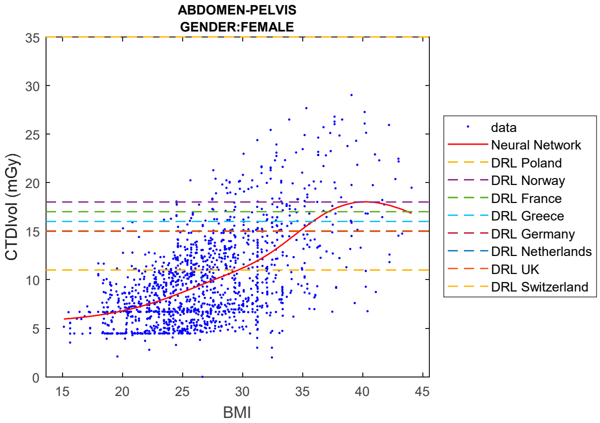

(k)

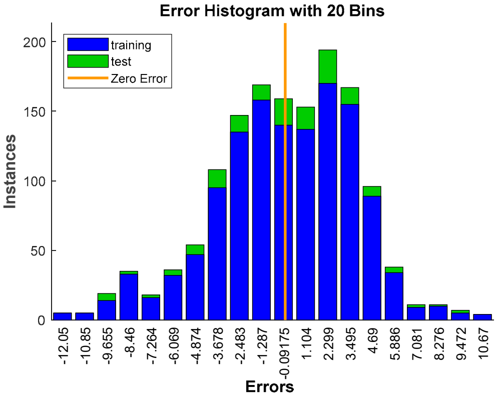

(l)

Figure S8. CTDIvol prediction according to BMI including European DRLs for women's Abdomen & Pelvis protocol (left), together with error histograms (right), employing the following ML techniques: *linear regression* (a) and (b), *regression trees* (c) and (d), '*bagged*' *regression trees* (e) and (f), *GPR* (g) and (h), *SVR* (i) and (j) and *neural networks* (k) and (l).

1.7. Men's Thorax Protocol

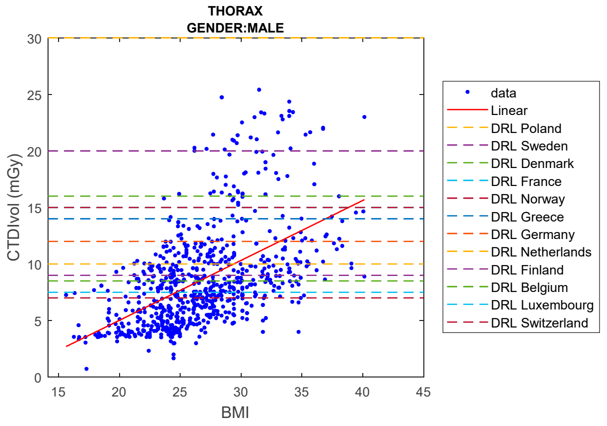

(a)

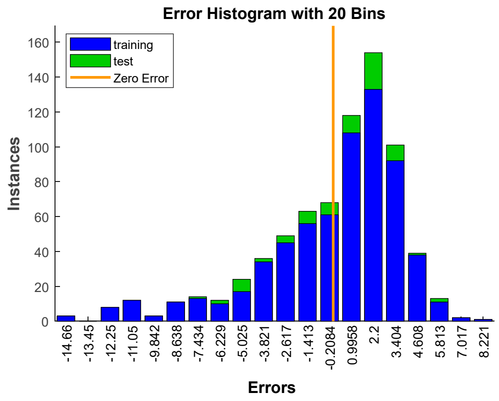

(b)

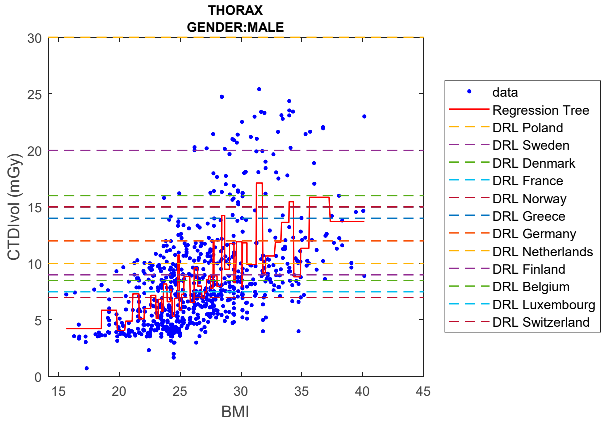

(c)

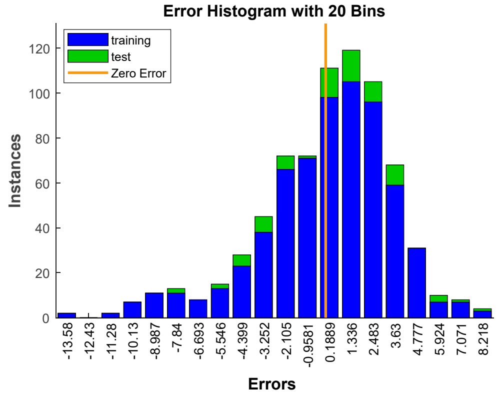

(d)

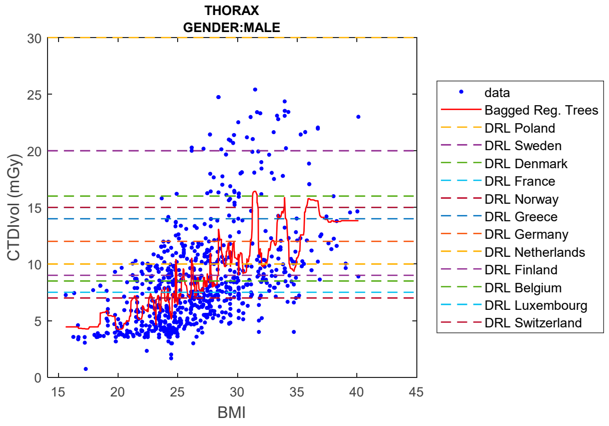

(e)

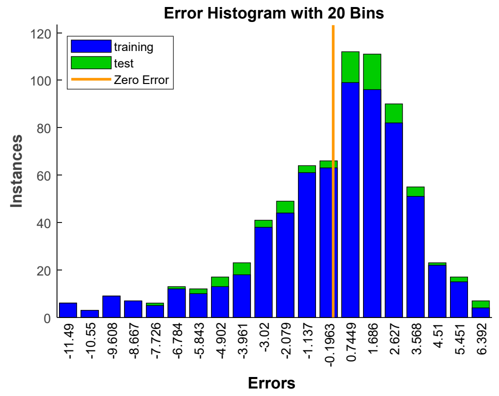

(f)

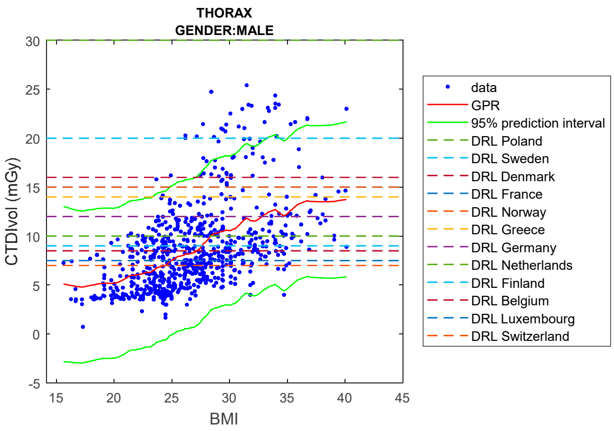

(g)

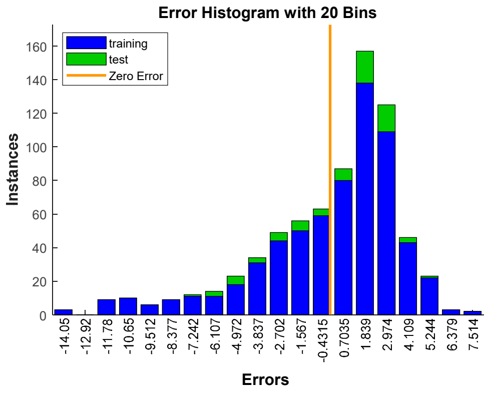

(h)

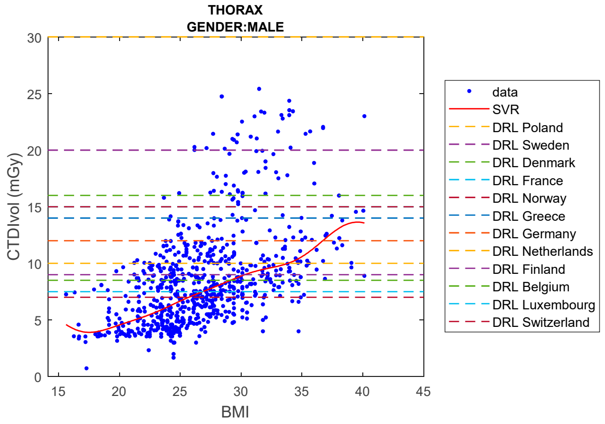

(i)

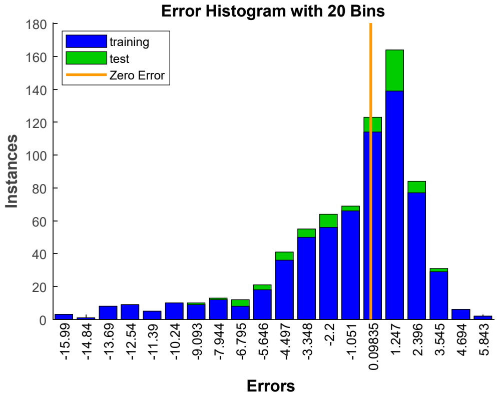

(j)

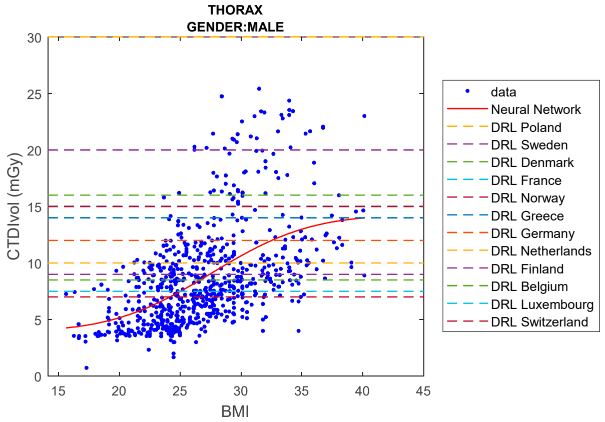

(k)

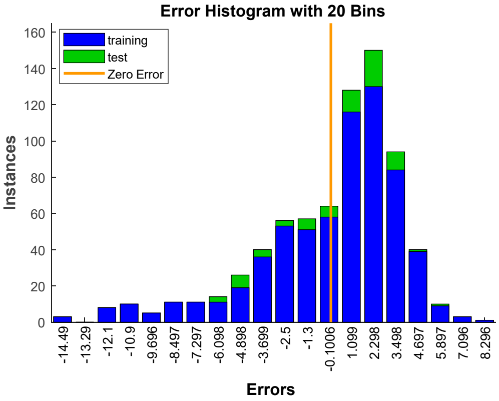

(l)

Figure S9. CTDI$_{VOL}$ prediction according to BMI including European DRLs for men's thorax protocol (left), together with error histograms (right), employing the following ML techniques: *linear regression* (a) and (b), *regression trees* (c) and (d), *'bagged' regression trees* (e) and (f), *GPR* (g) and (h), *SVR* (i) and (j) and *neural networks* (k) and (l).

1.8. Women's Thorax Protocol

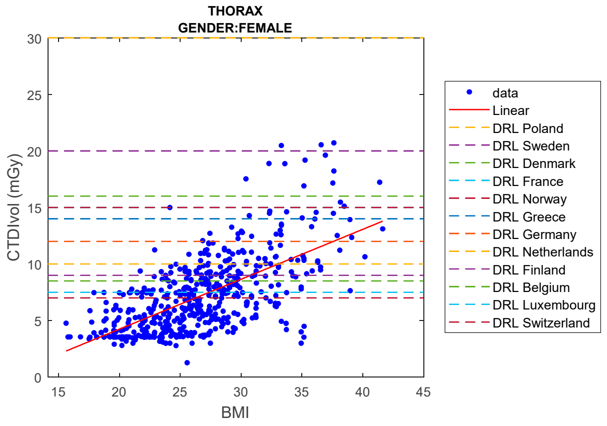

(a)

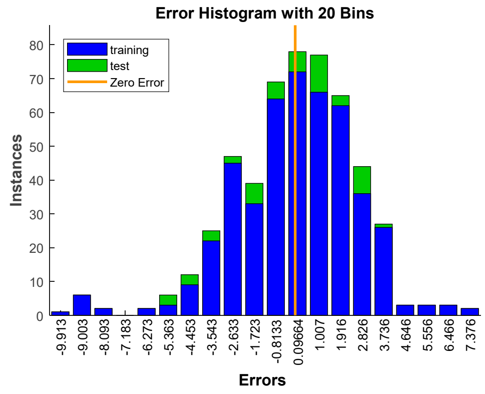

(b)

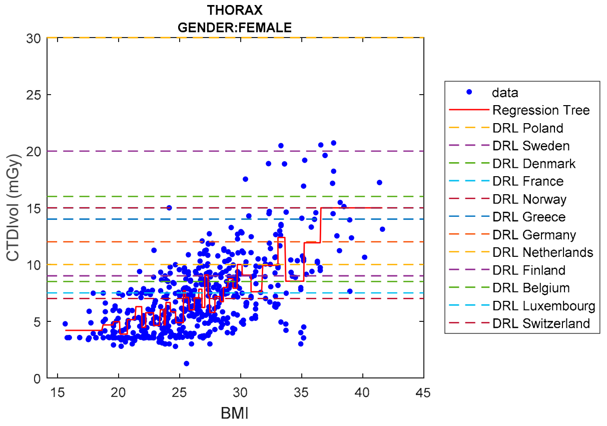

(c)

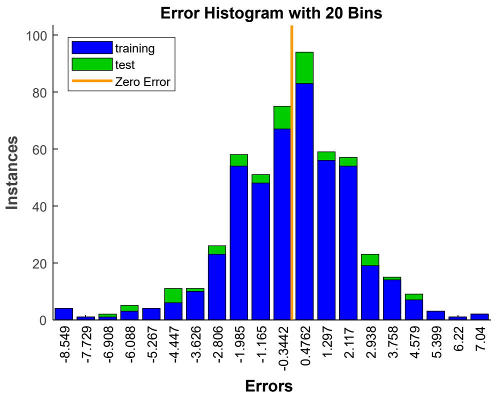

(d)

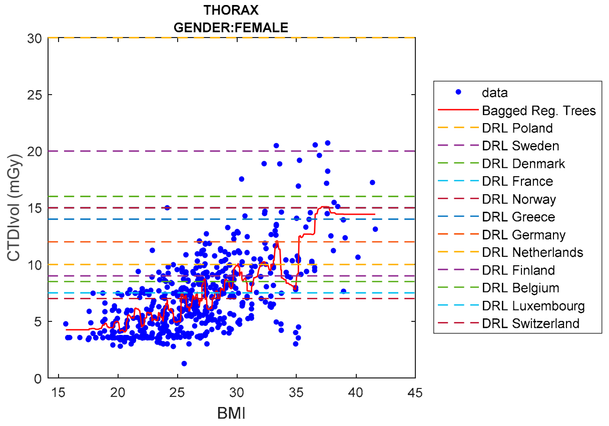

(e)

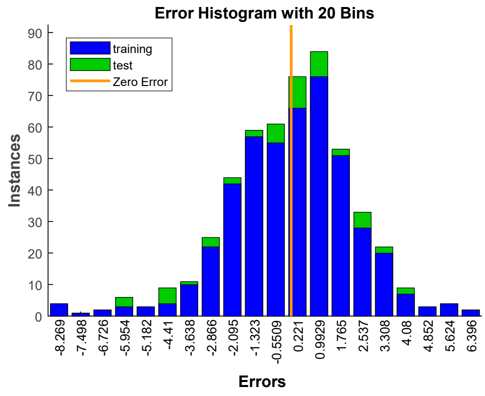

(f)

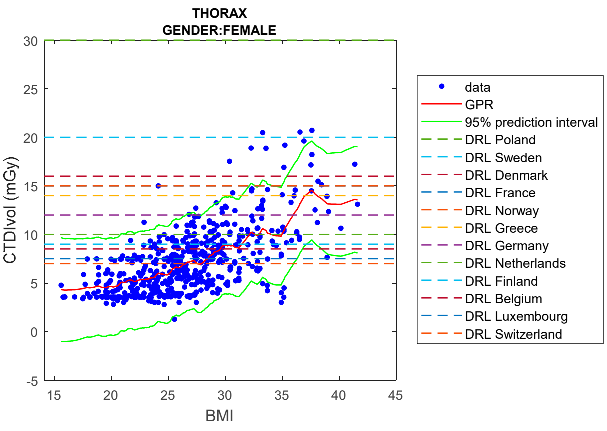

(g)

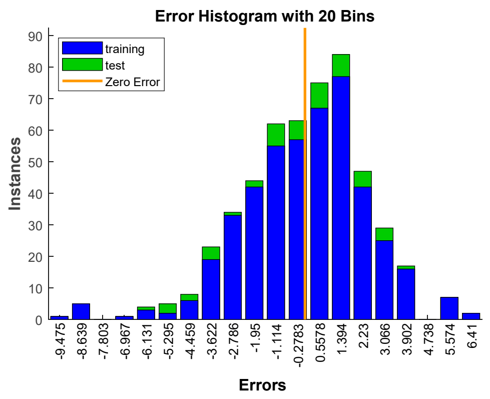

(h)

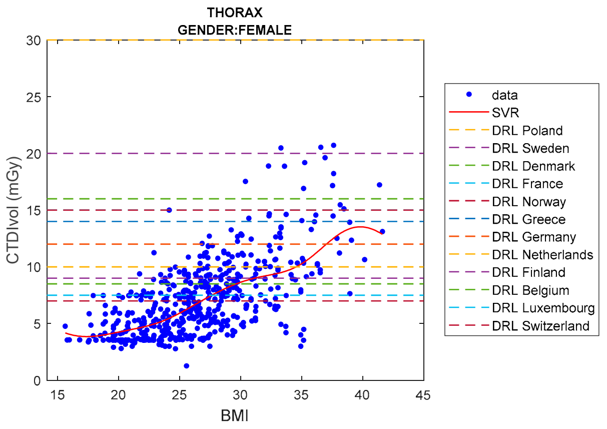

(i)

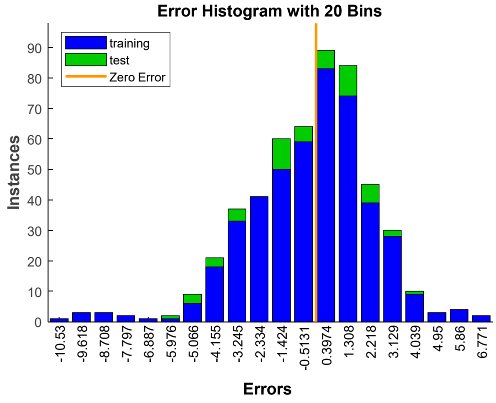

(j)

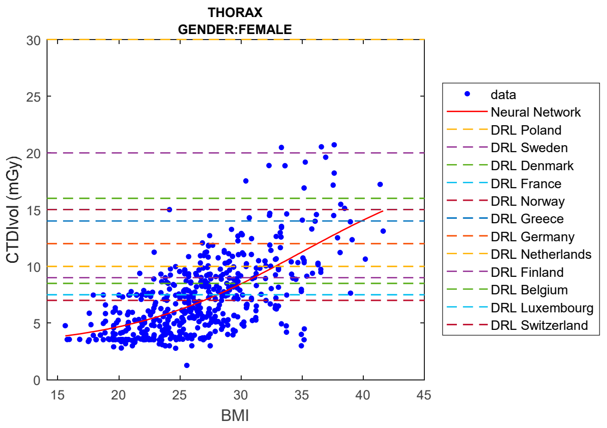

(k)

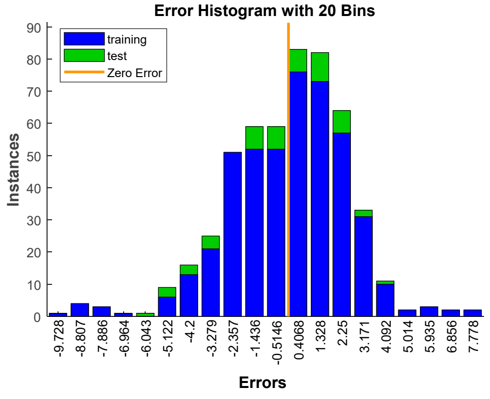

(l)

Figure S10. CTDIvol prediction according to BMI including European DRLs for women's Thorax protocol (left), together with error histograms (right), employing the following ML techniques: *linear regression* (a) and (b), *regression trees* (c) and (d), '*bagged*' *regression trees* (e) and (f), *GPR* (g) and (h), *SVR* (i) and (j) and *neural networks* (k) and (l).

1.9. Men's Abdomen Protocol

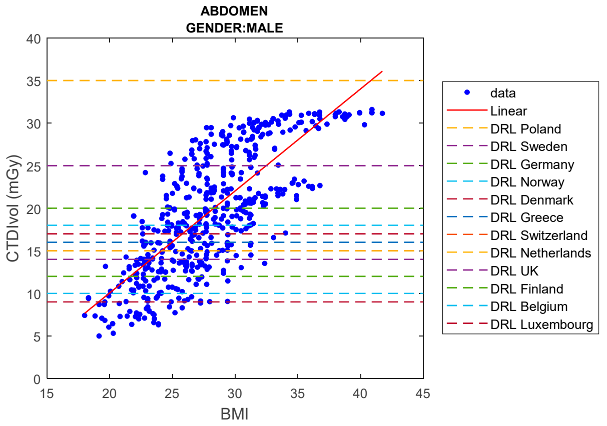

(a)

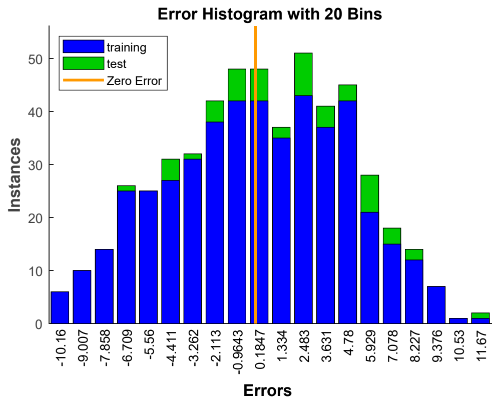

(b)

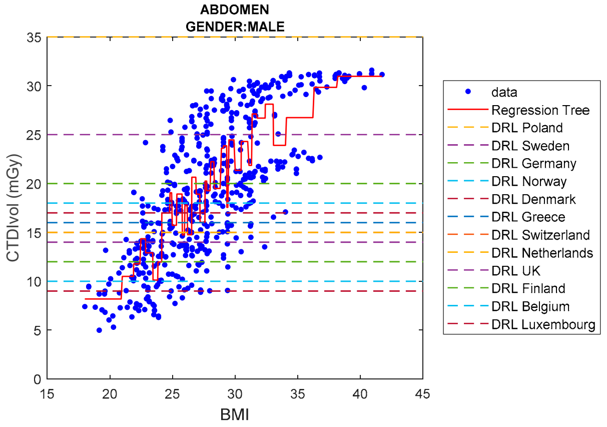

(c)

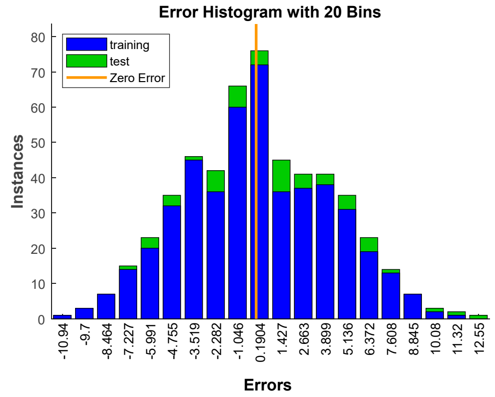

(d)

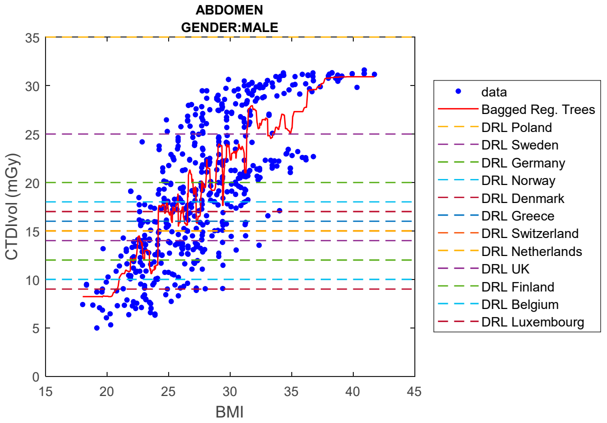

(e)

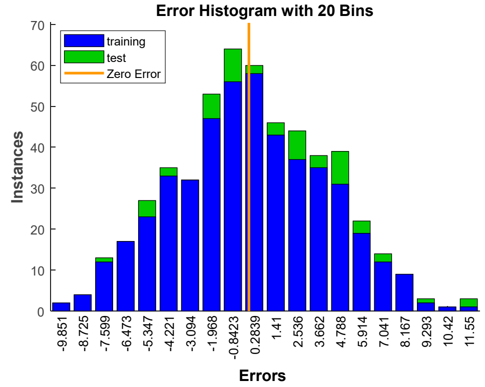

(f)

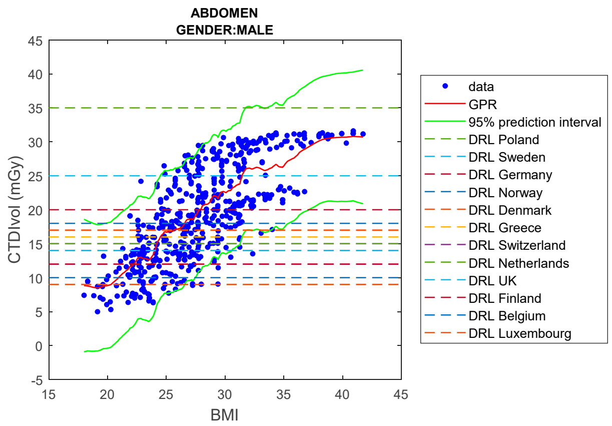

(g)

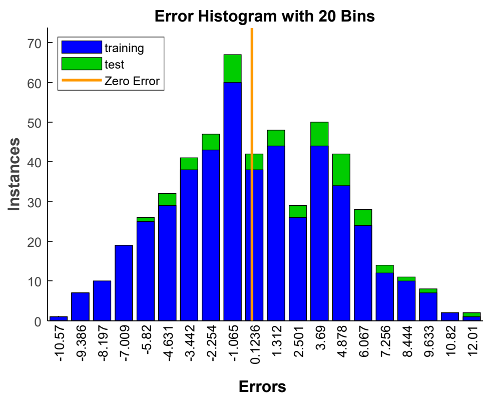

(h)

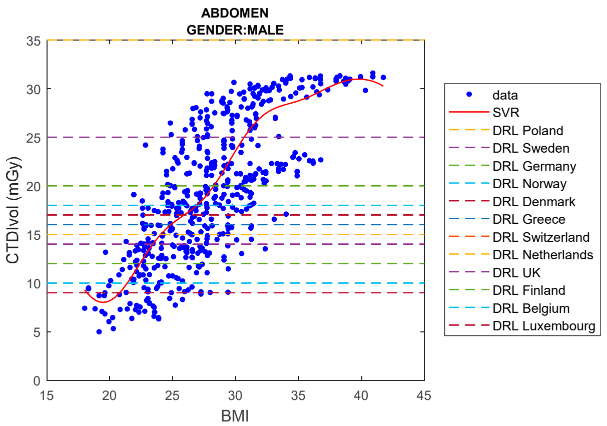

(i)

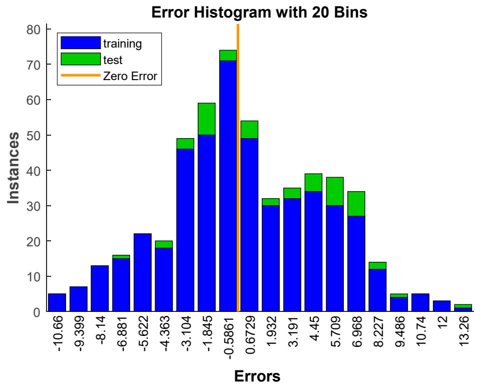

(j)

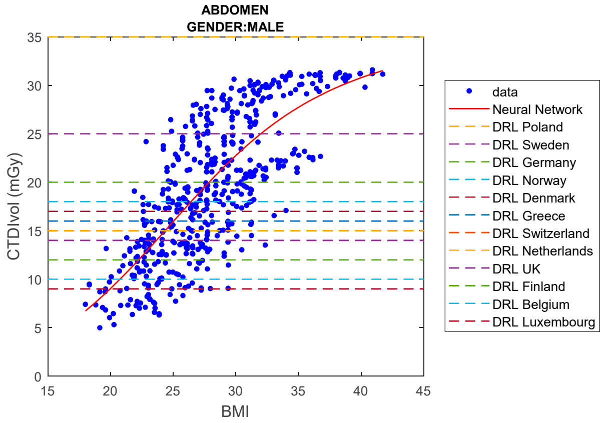

(k)

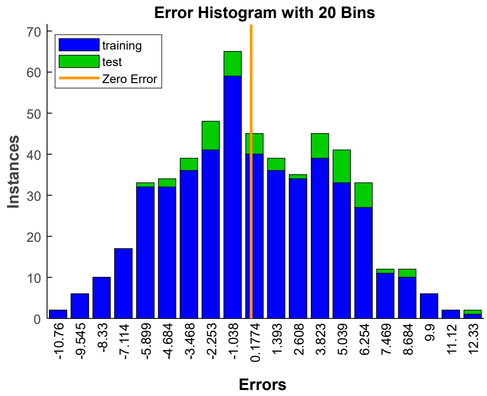

(l)

Figure S11. CTDIvol prediction according to BMI including European DRLs for men's abdomen protocol (left), together with error histograms (right), employing the following ML techniques: *linear regression* (a) and (b), *regression trees* (c) and (d), '*bagged*' *regression trees* (e) and (f), *GPR* (g) and (h), *SVR* (i) and (j) and *neural networks* (k) and (l).

1.10. Women's Abdomen Protocol

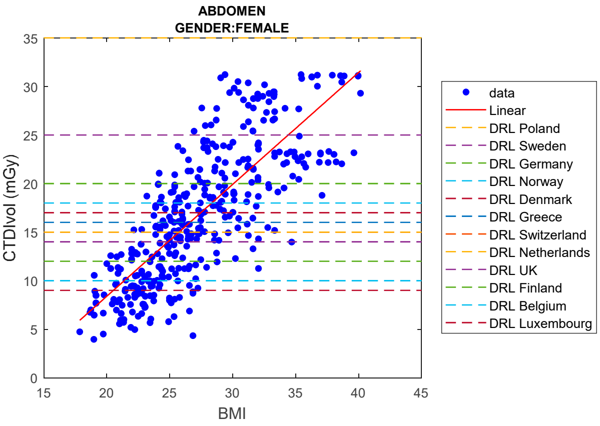

(a)

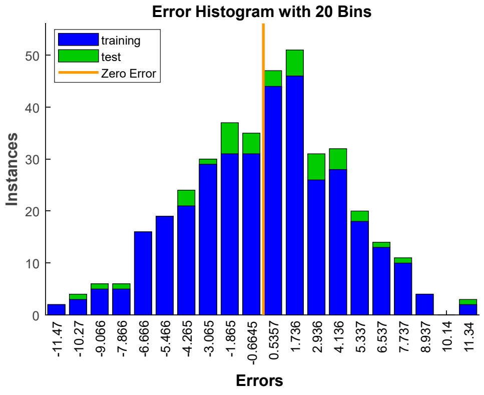

(b)

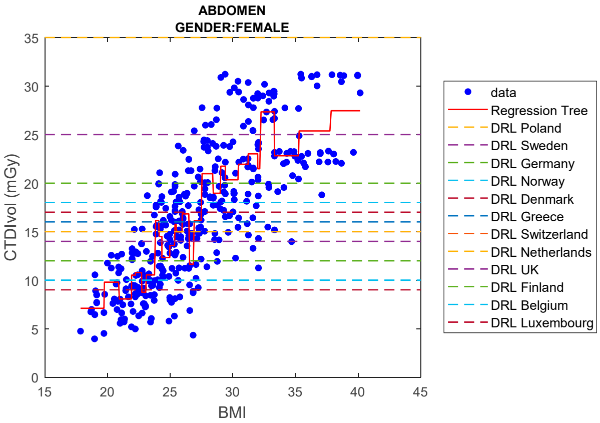

(c)

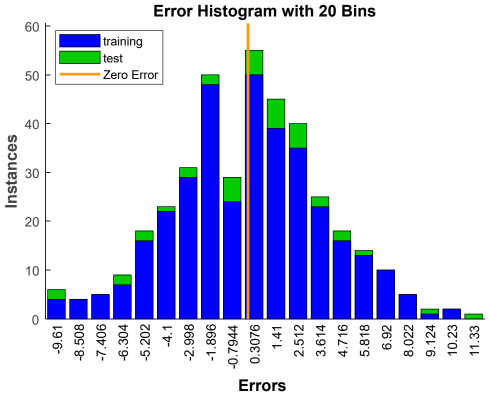

(d)

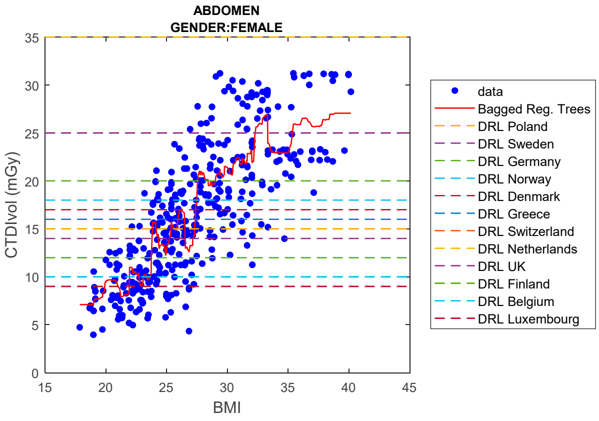

(e)

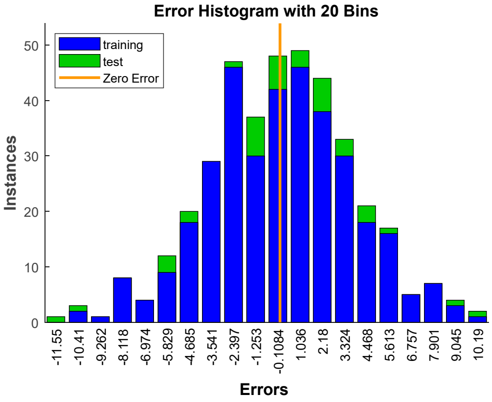

(f)

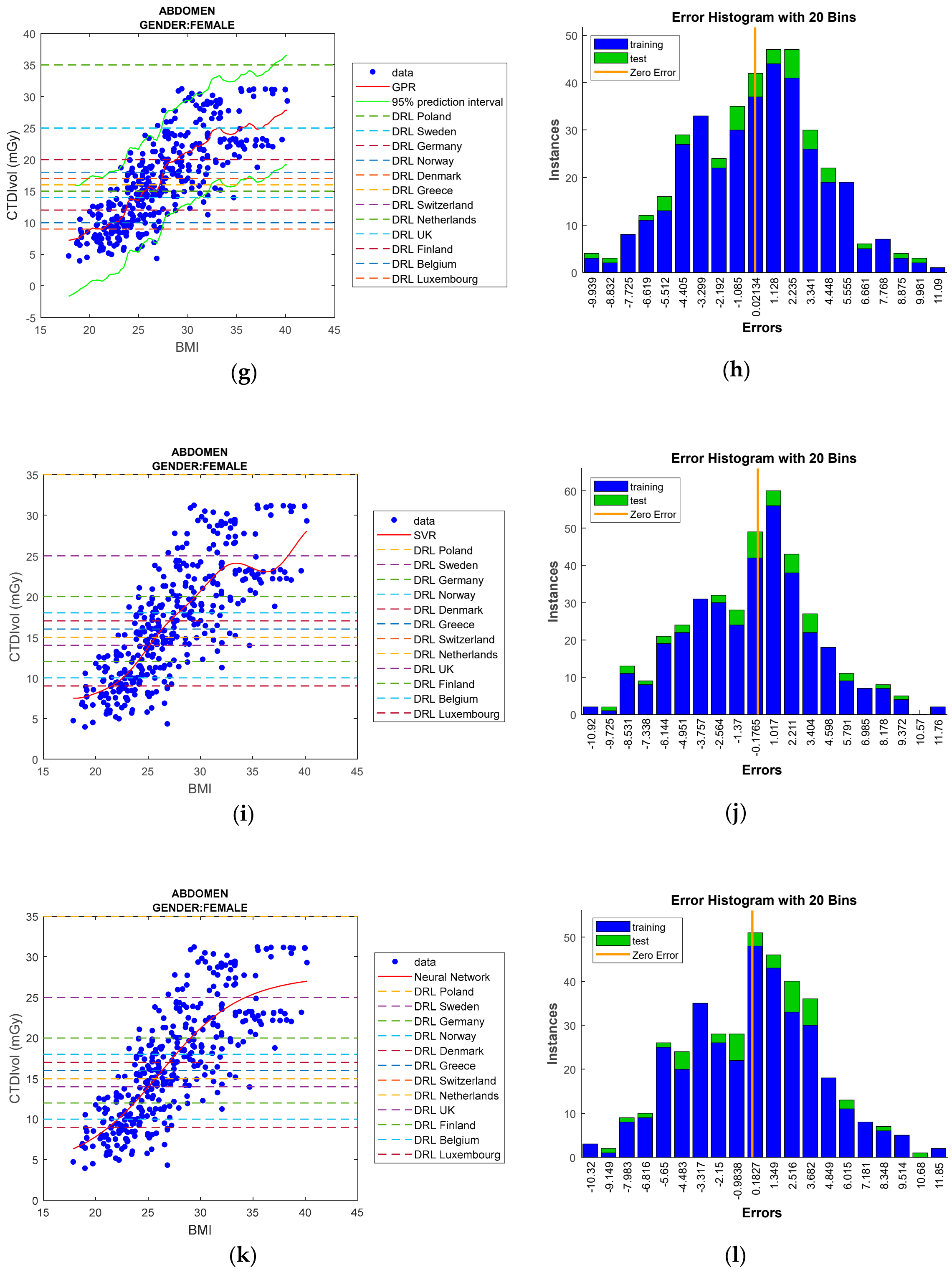

Figure S12. CTDIvol prediction according to BMI including European DRLs for women's Abdomen protocol (left), together with error histograms (right), employing the following ML techniques: *linear regression* (a) and (b), *regression trees* (c) and (d), '*bagged*' *regression trees* (e) and (f), *GPR* (g) and (h), *SVR* (i) and (j) and *neural networks* (k) and (l).

2. Comparison between SSDE and BMI

2.1. Skull Protocol

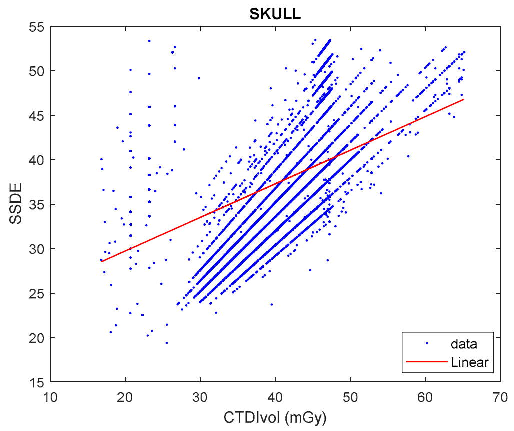

(a)

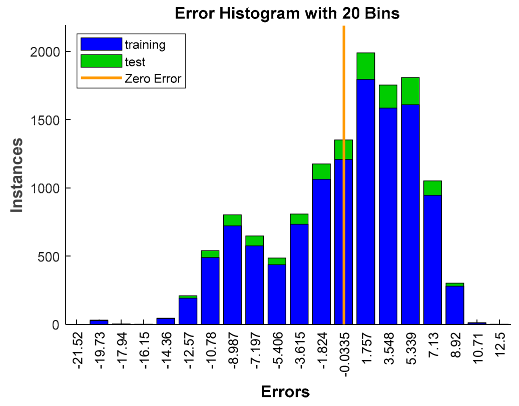

(b)

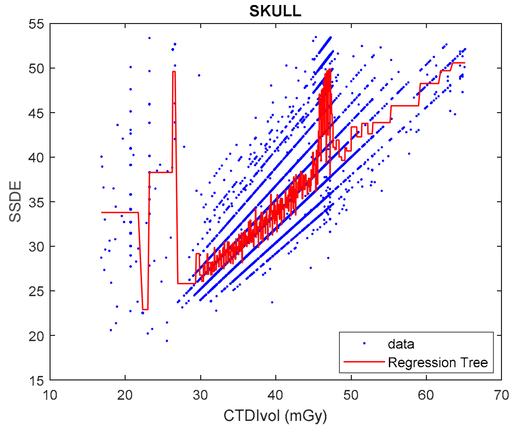

(c)

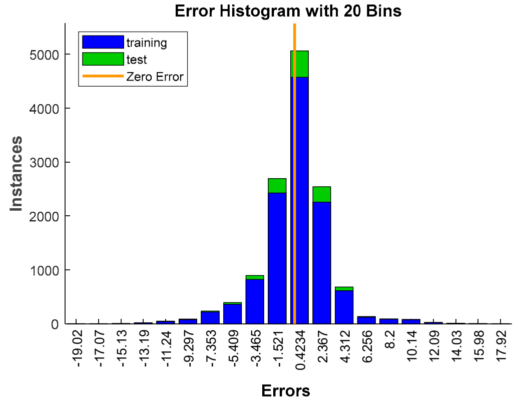

(d)

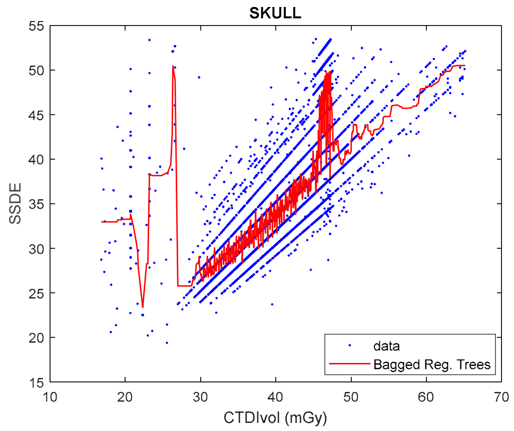

(e)

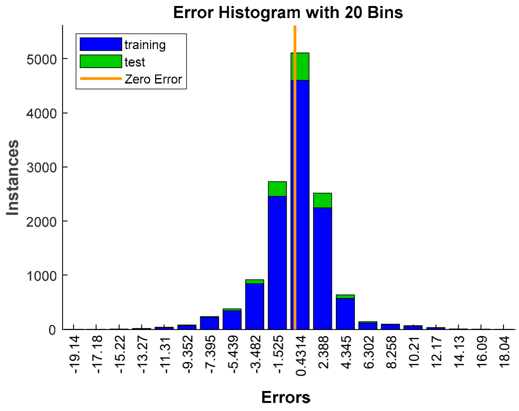

(f)

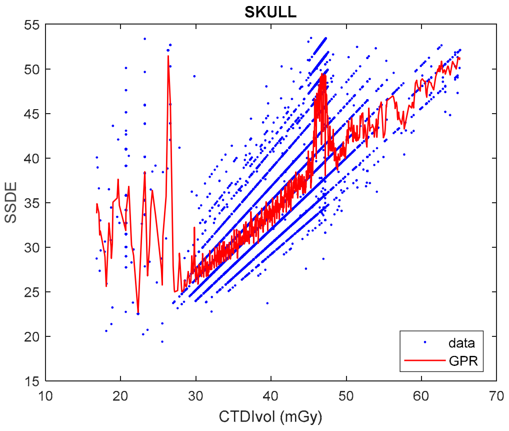

(g)

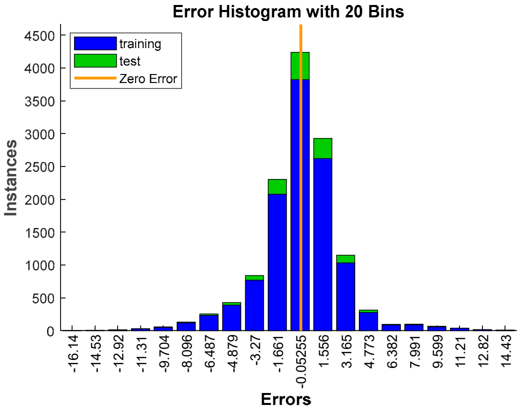

(h)

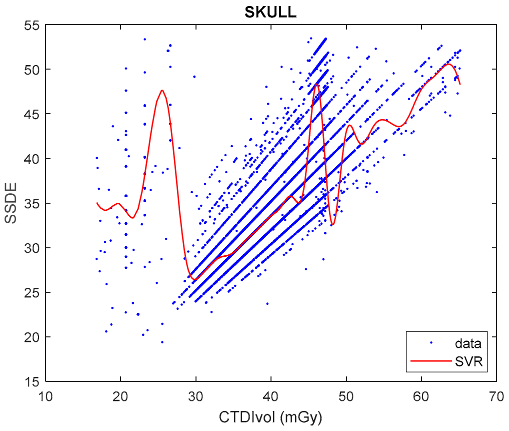

(i)

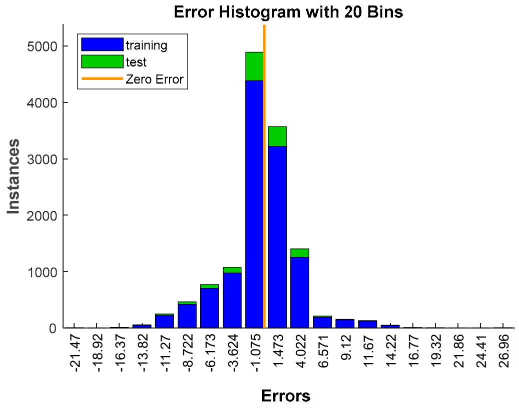

(j)

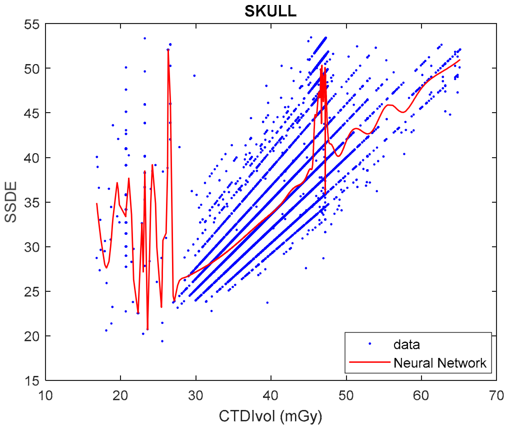

(k)

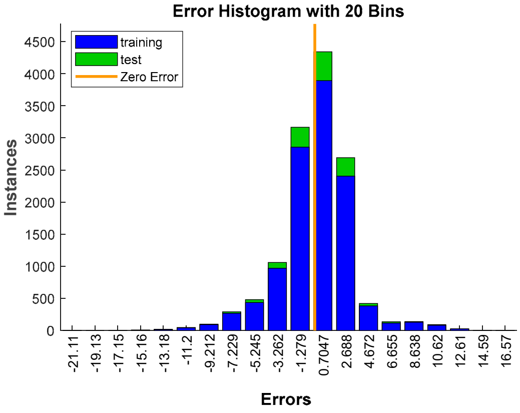

(l)

Figure S13. SSDE prediction according to CTDIVOL for skull protocol (left), together with error histograms (right), employing the following ML techniques: *linear regression* (a) and (b), *regression trees* (c) and (d), ‘*bagged*’ *regression trees* (e) and (f), *GPR* (g) and (h), *SVR* (i) and (j) and *neural networks* (k) and (l).

2.2. Thorax, Abdomen and Pelvis Protocol

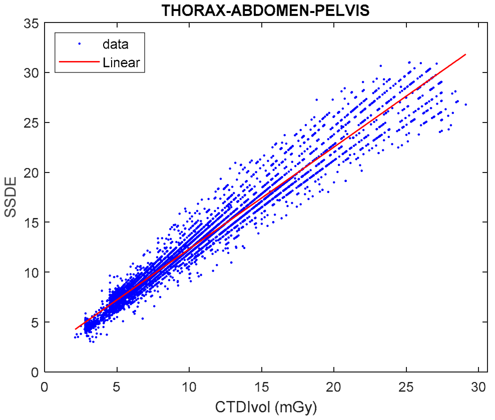

(a)

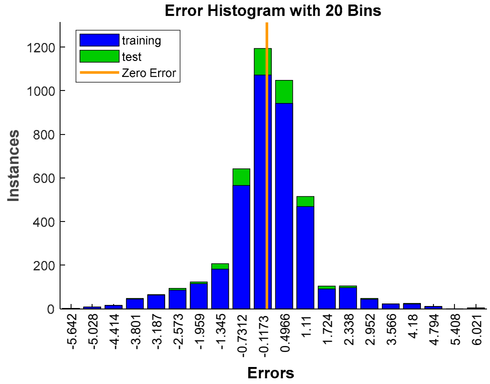

(b)

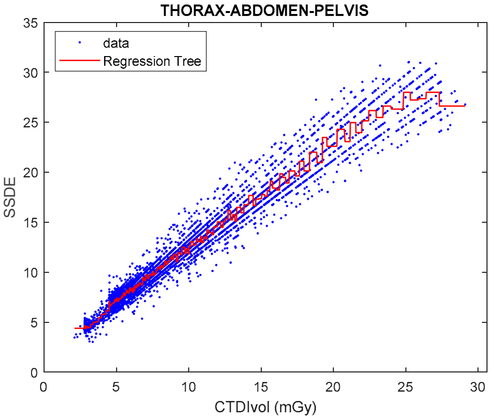

(c)

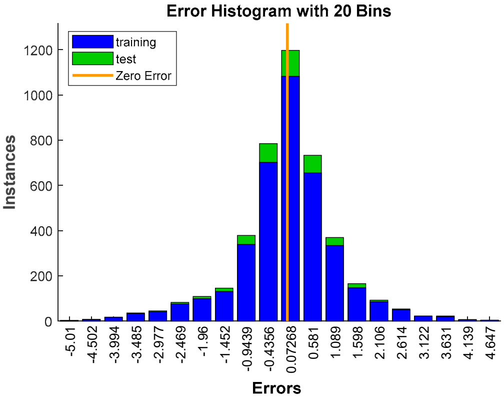

(d)

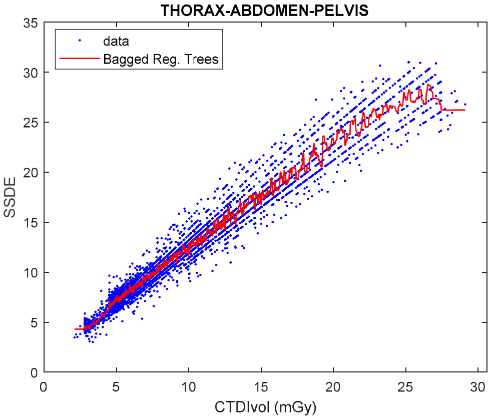

(e)

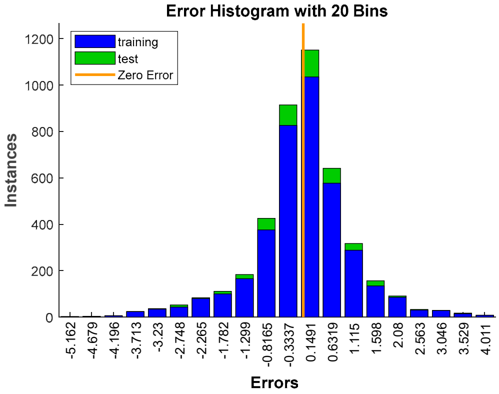

(f)

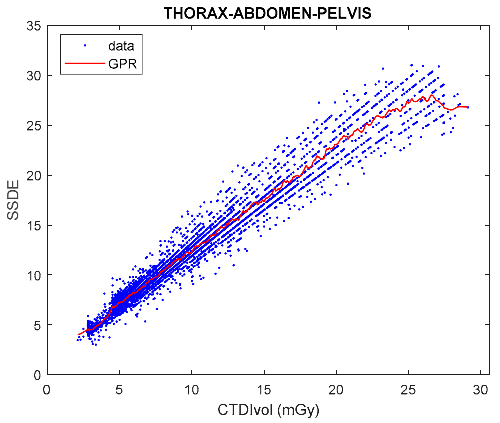

(g)

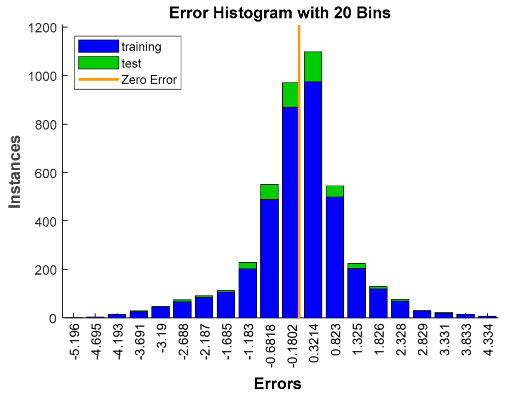

(h)

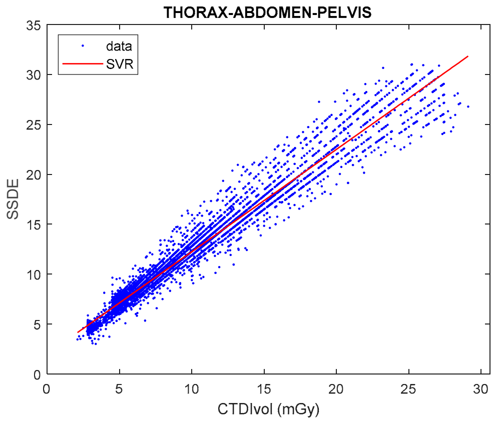

(i)

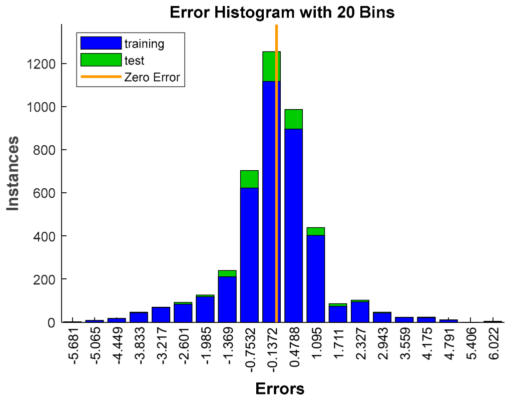

(j)

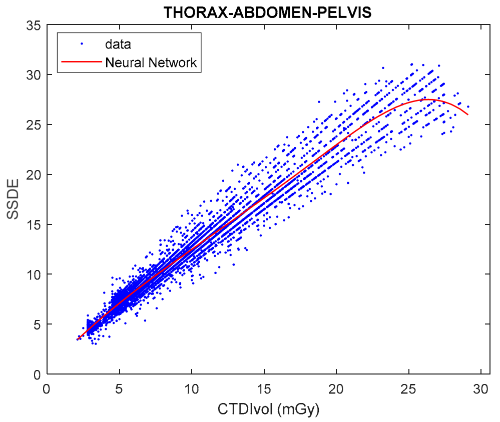

(k)

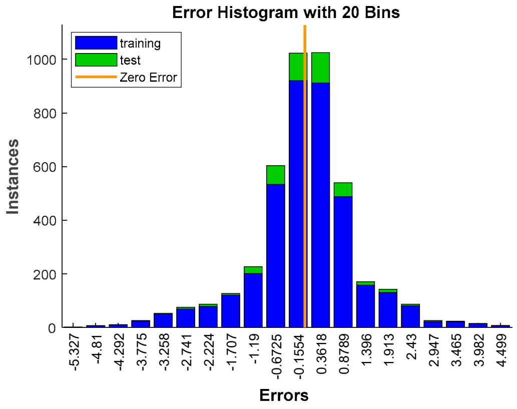

(l)

Figure S14. SSDE prediction according to $CTDI_{VOL}$ for Thorax, Abdomen & Pelvis protocol (left), together with error histograms (right), employing the following ML techniques: *linear regression* (a) and (b), *regression trees* (c) and (d), '*bagged' regression trees* (e) and (f), *GPR* (g) and (h), *SVR* (i) and (j) and *neural networks* (k) and (l).

2.3. Abdomen and Pelvis Protocol

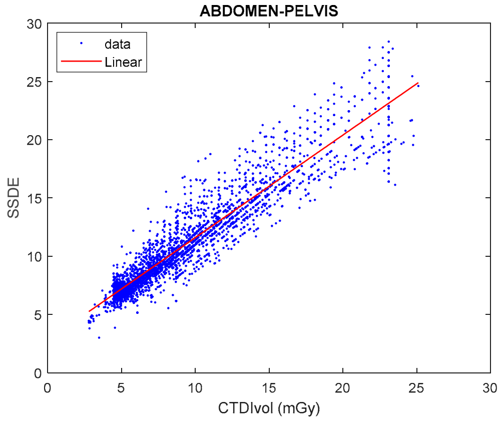

(a)

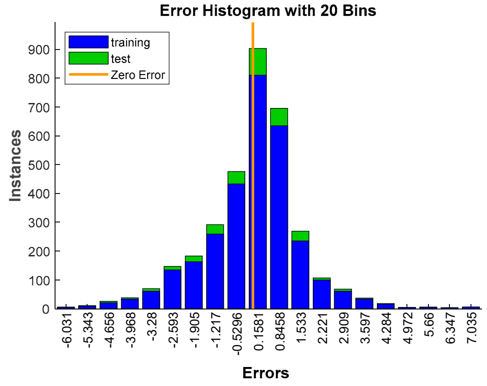

(b)

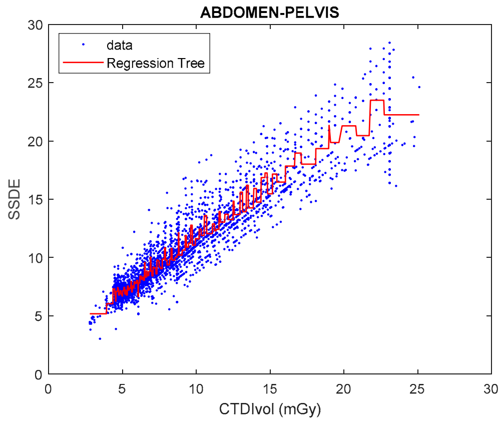

(c)

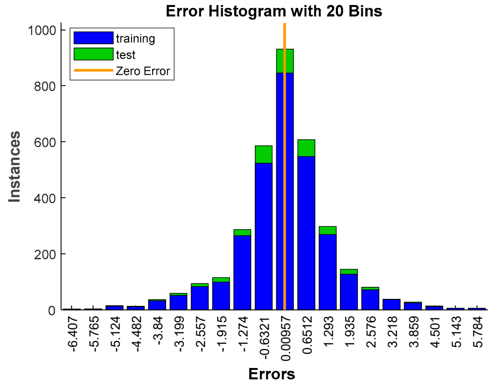

(d)

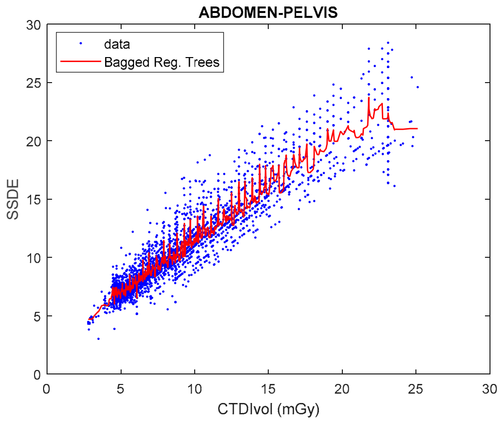

(e)

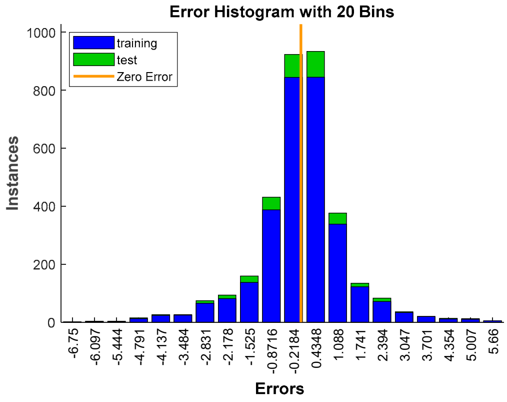

(f)

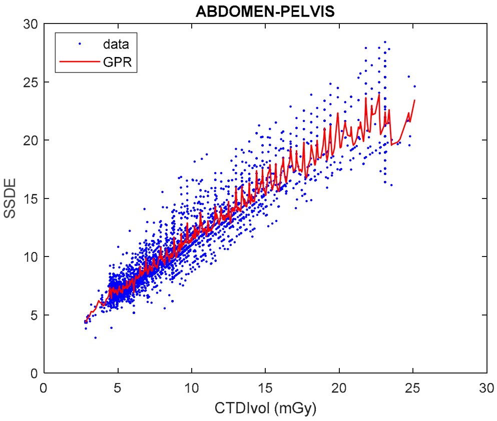

(g)

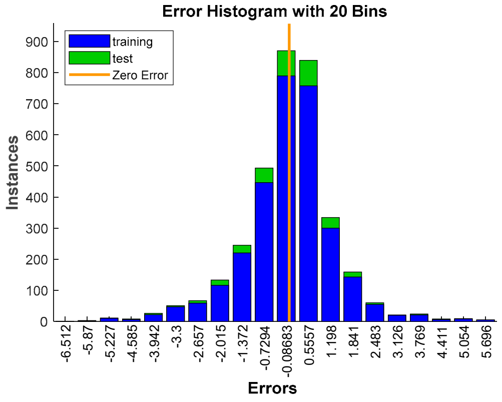

(h)

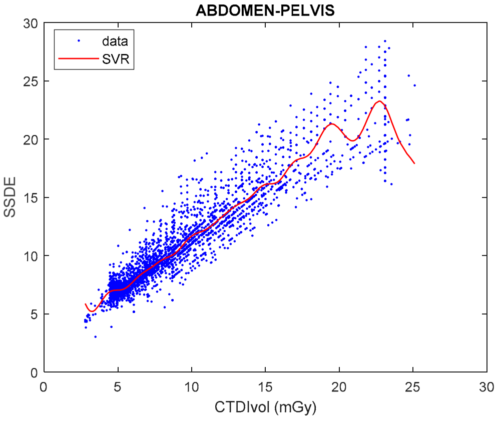

(i)

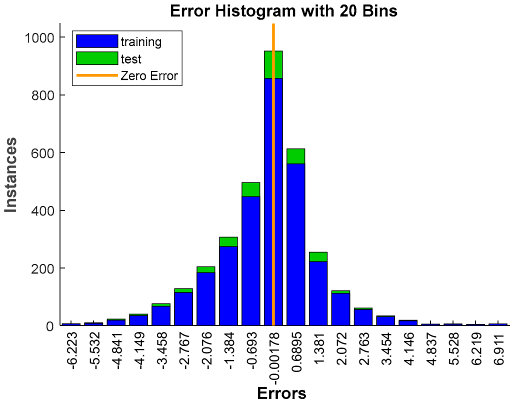

(j)

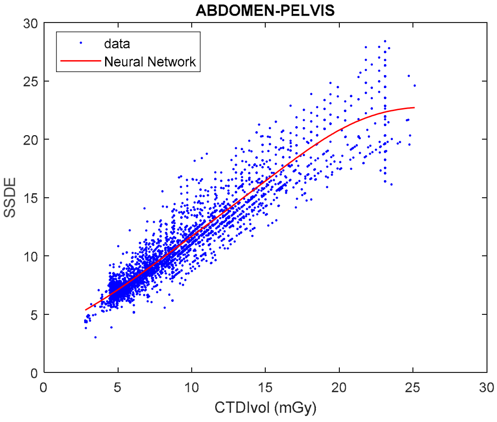

(k)

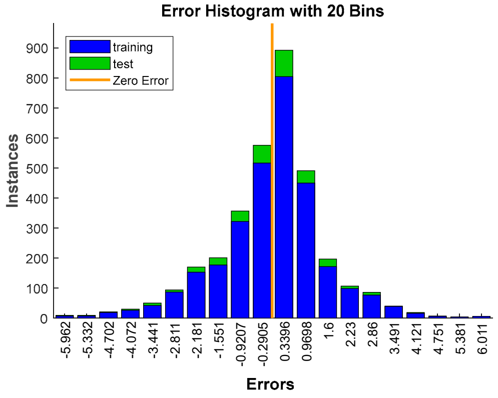

(l)

Figure S15. SSDE prediction according to CTDIvol for Abdomen & Pelvis protocol (left), together with error histograms (right), employing the following ML techniques: *linear regression* (a) and (b), *regression trees* (c) and (d), '*bagged*' *regression trees* (e) and (f), *GPR* (g) and (h), *SVR* (i) and (j) and *neural networks* (k) and (l).

2.4. Thorax Protocol

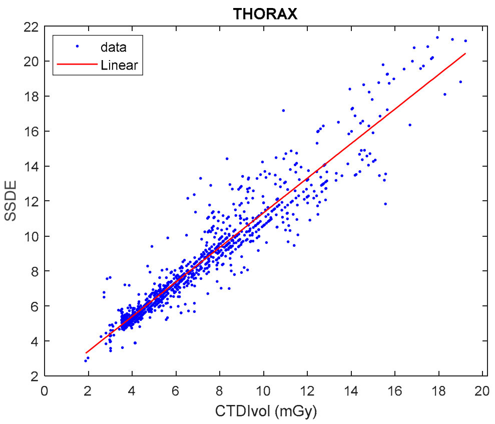

(a)

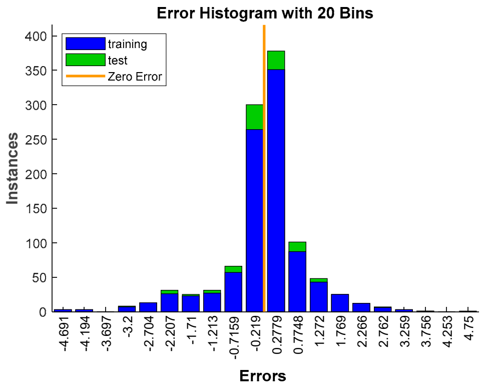

(b)

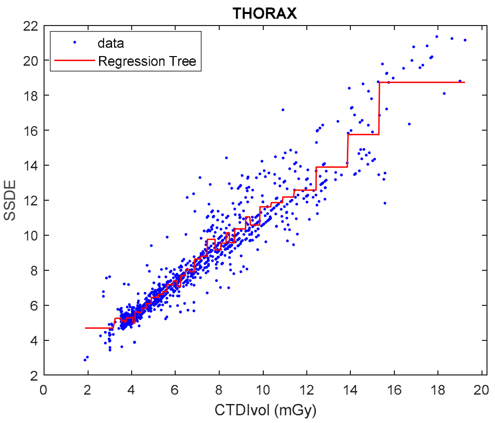

(c)

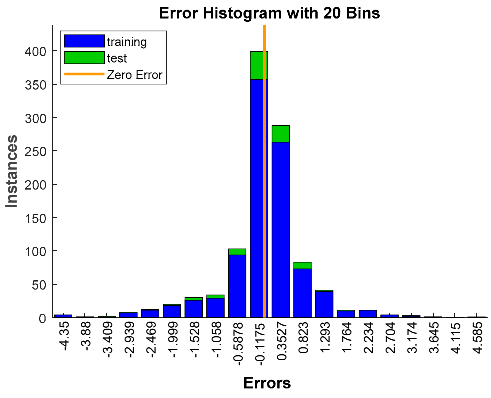

(d)

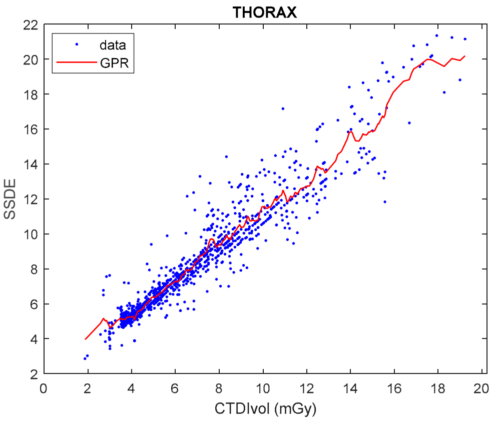

(e)

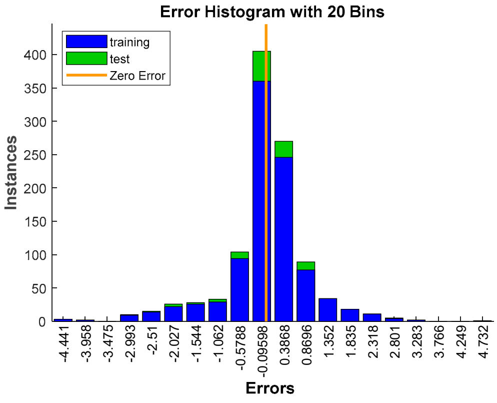

(f)

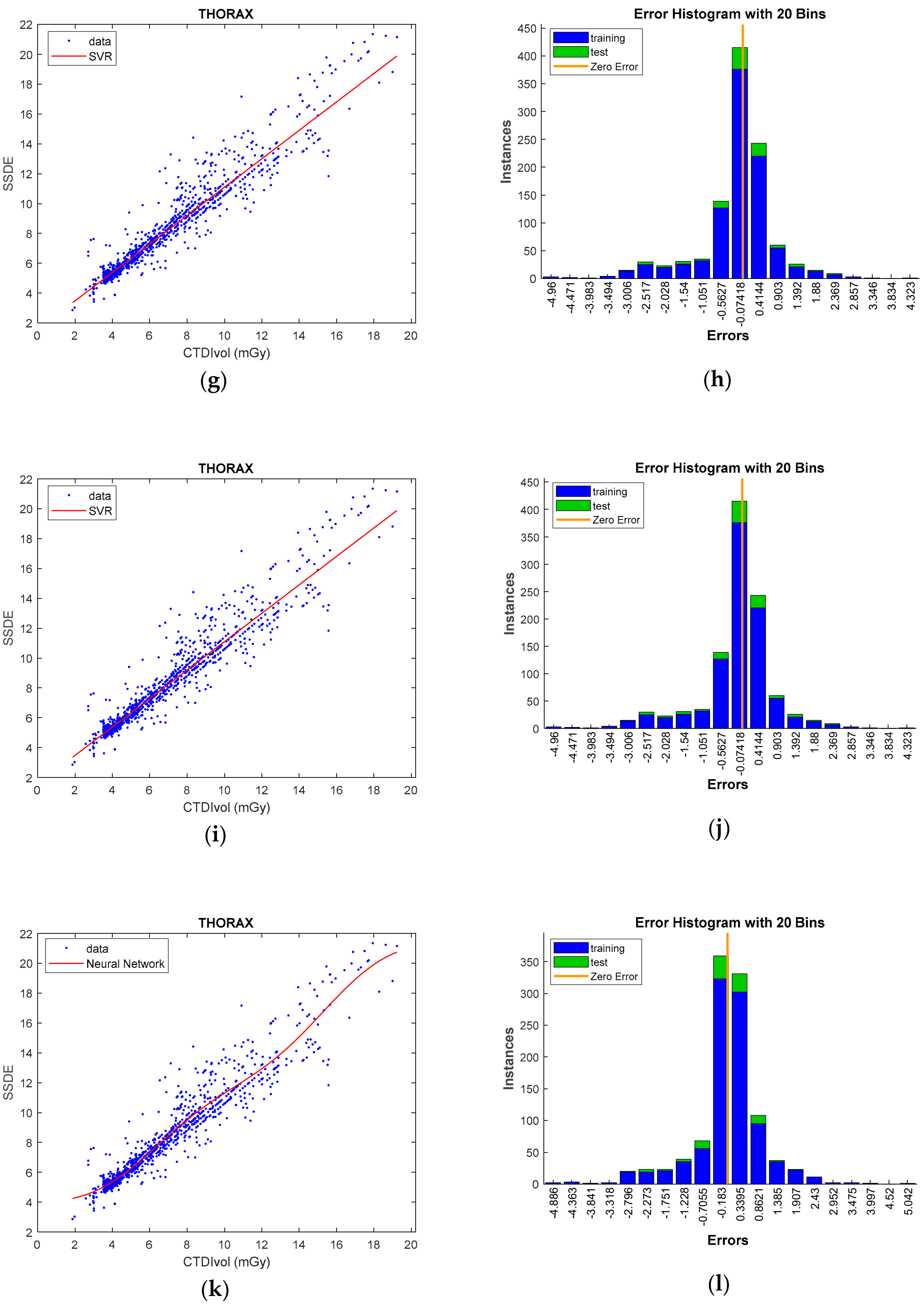

Figure S16. SSDE prediction according to CTDIvol for Thorax protocol (left), together with error histograms (right), employing the following ML techniques: *linear regression* (a) and (b), *regression trees* (c) and (d), '*bagged' regression trees* (e) and (f), *GPR* (g) and (h), *SVR* (i) and (j) and *neural networks* (k) and (l).

2.5. Abdomen Protocol

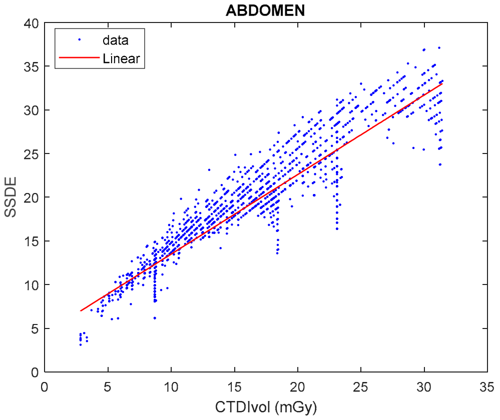

(a)

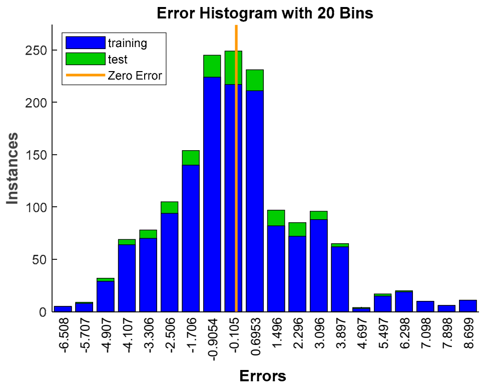

(b)

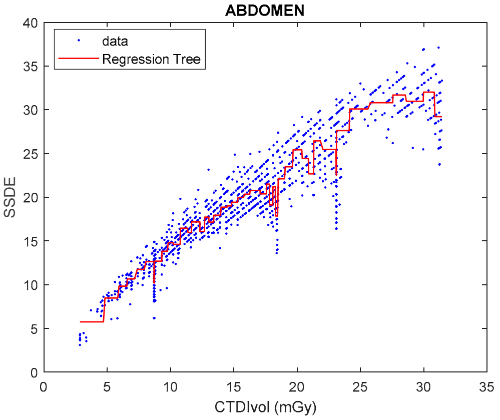

(c)

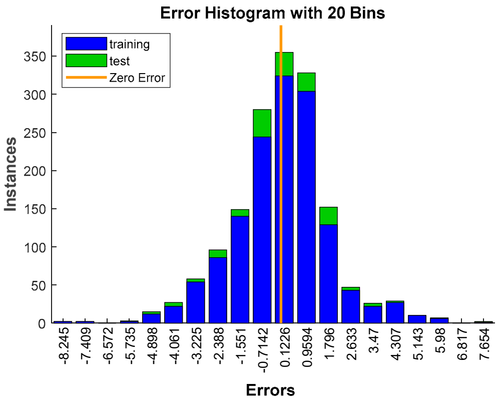

(d)

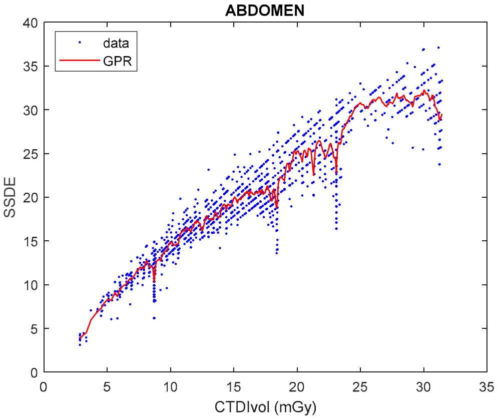

(e)

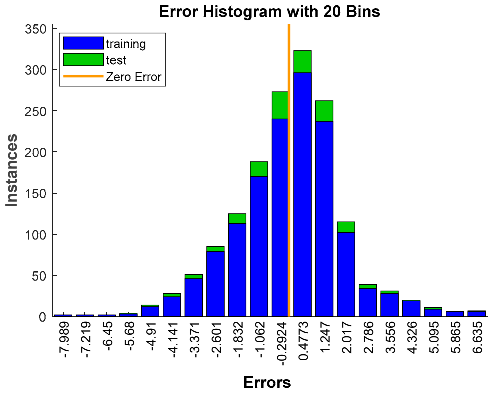

(f)

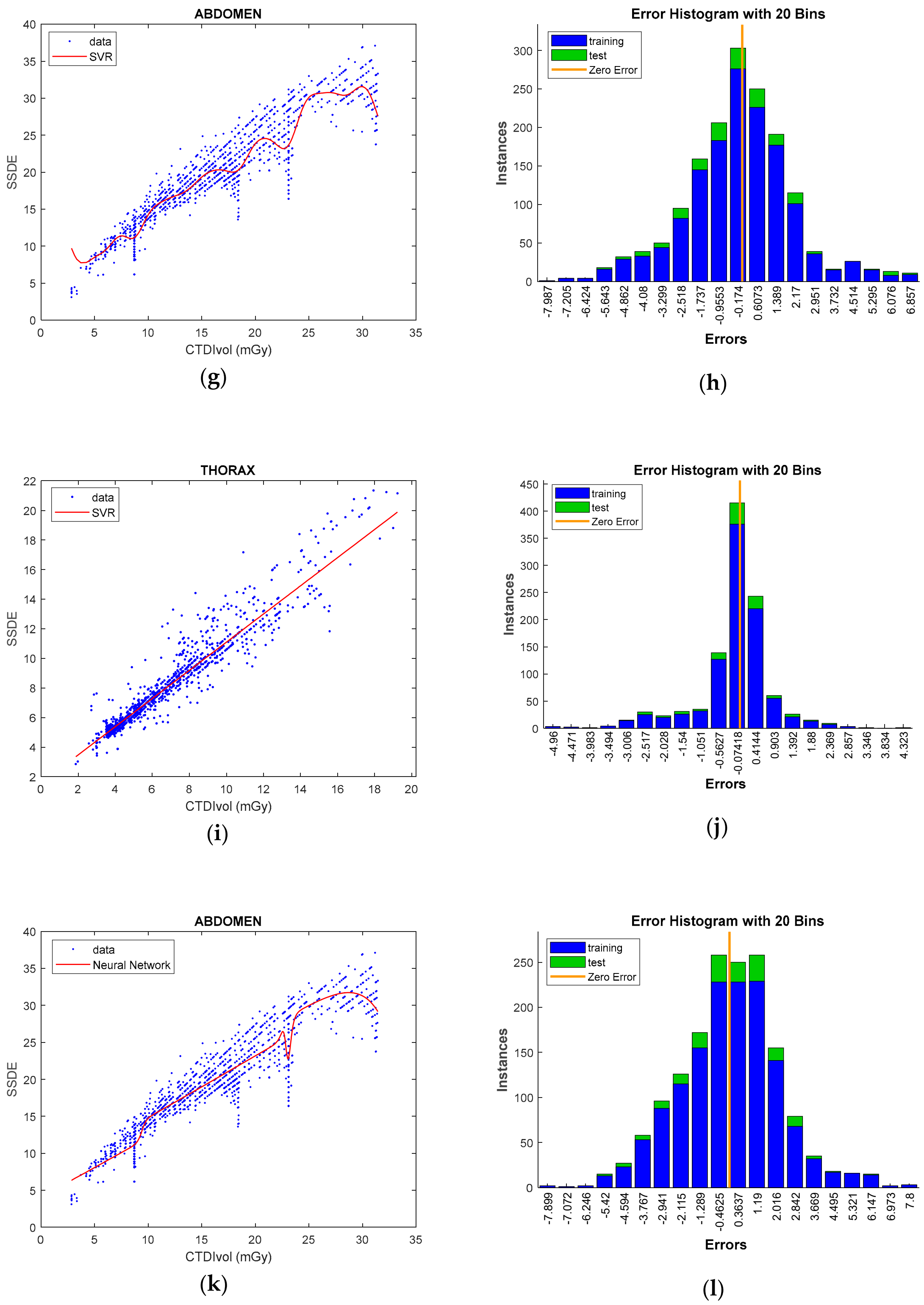

Figure S17. SSDE prediction according to CTDI$_{VOL}$ for Abdomen protocol (left), together with error histograms (right), employing the following ML techniques: *linear regression* (a) and (b), *regression trees* (c) and (d), '*bagged' regression trees* (e) and (f), *GPR* (g) and (h), *SVR* (i) and (j) and *neural networks* (k) and (l).
